# Quality Evaluation of Free-living Validation Studies for the Assessment of 24-Hour Physical Behavior in Adults via Wearables: Systematic Review

**DOI:** 10.2196/36377

**Published:** 2022-06-09

**Authors:** Marco Giurgiu, Irina Timm, Marlissa Becker, Steffen Schmidt, Kathrin Wunsch, Rebecca Nissen, Denis Davidovski, Johannes B J Bussmann, Claudio R Nigg, Markus Reichert, Ulrich W Ebner-Priemer, Alexander Woll, Birte von Haaren-Mack

**Affiliations:** 1 Department of Sports and Sports Science, Karlsruhe Institute of Technology Karlsruhe Germany; 2 Department of Psychiatry and Psychotherapy, Central Institute of Mental Health, Medical Faculty Mannheim, Heidelberg University Mannheim Germany; 3 Unit Physiotherapy, Department of Orthopedics, Erasmus MC, University Medical Center Rotterdam Rotterdam Netherlands; 4 Department of Rehabilitation Medicine, Erasmus MC, University Medical Center Rotterdam Rotterdam Netherlands; 5 Health Science Department, Institute of Sport Science, University of Bern Bern Switzerland; 6 Department of eHealth and Sports Analytics, Faculty of Sport Science, Ruhr-University Bochum Bochum Germany; 7 Department of Health and Social Psychology, Institute of Psychology, German Sport University Cologne Germany

**Keywords:** wearables, validation, sedentary behavior, physical activity, sleep

## Abstract

**Background:**

Wearable technology is a leading fitness trend in the growing commercial industry and an established method for collecting 24-hour physical behavior data in research studies. High-quality free-living validation studies are required to enable both researchers and consumers to make guided decisions on which study to rely on and which device to use. However, reviews focusing on the quality of free-living validation studies in adults are lacking.

**Objective:**

This study aimed to raise researchers’ and consumers’ attention to the quality of published validation protocols while aiming to identify and compare specific consistencies or inconsistencies between protocols. We aimed to provide a comprehensive and historical overview of which wearable devices have been validated for which purpose and whether they show promise for use in further studies.

**Methods:**

Peer-reviewed validation studies from electronic databases, as well as backward and forward citation searches (1970 to July 2021), with the following, required indicators were included: protocol must include real-life conditions, outcome must belong to one dimension of the 24-hour physical behavior construct (intensity, posture or activity type, and biological state), the protocol must include a criterion measure, and study results must be published in English-language journals. The risk of bias was evaluated using the Quality Assessment of Diagnostic Accuracy Studies-2 tool with 9 questions separated into 4 domains (patient selection or study design, index measure, criterion measure, and flow and time).

**Results:**

Of the 13,285 unique search results, 222 (1.67%) articles were included. Most studies (153/237, 64.6%) validated an intensity measure outcome such as energy expenditure. However, only 19.8% (47/237) validated biological state and 15.6% (37/237) validated posture or activity-type outcomes. Across all studies, 163 different wearables were identified. Of these, 58.9% (96/163) were validated only once. ActiGraph GT3X/GT3X+ (36/163, 22.1%), Fitbit Flex (20/163, 12.3%), and ActivPAL (12/163, 7.4%) were used most often in the included studies. The percentage of participants meeting the quality criteria ranged from 38.8% (92/237) to 92.4% (219/237). On the basis of our classification tree to evaluate the overall study quality, 4.6% (11/237) of studies were classified as *low risk*. Furthermore, 16% (38/237) of studies were classified as having *some concerns*, and 72.9% (173/237) of studies were classified as *high risk*.

**Conclusions:**

Overall, free-living validation studies of wearables are characterized by low methodological quality, large variability in design, and focus on intensity. Future research should strongly aim at biological state and posture or activity outcomes and strive for standardized protocols embedded in a validation framework. Standardized protocols for free-living validation embedded in a framework are urgently needed to inform and guide stakeholders (eg, manufacturers, scientists, and consumers) in selecting wearables for self-tracking purposes, applying wearables in health studies, and fostering innovation to achieve improved validity.

## Introduction

### 24-Hour Physical Behavior

Although physical activity (PA) has been commonly assessed using self-report measures in the past decades, device-based measurement of PA research has been on the rise for several years. This method has also developed since its implementation and moved forward from assessing single parameters such as steps and counts, over the assessment of sedentary behavior (SB) and PA in parallel, to an integrated perspective of different movement and nonmovement patterns—the so-called 24-hour activity cycle (24-HAC) [1,2]. Studies have shown that these different parameters independently contribute to health. Hence, the current World Health Organization guidelines [3] encourage adults and older adults to increase the time spent on PA while simultaneously limiting the amount of sedentary time. Positive implications can be expected for overall physical and mental health throughout the life span, including having a healthy sleep pattern to this recommendation.

This apparent shift from investigating a single behavior such as PA to a multi-perspective focus on 24-hour physical behavior (ie, including sleep, SB, and PA) has also been theoretically addressed. Rosenberger et al [1] introduced the 24-HAC model as a new paradigm for PA, and Trembley et al [2] provided a conceptual model of movement-based terminology around the 24-hour cycle. This approach was further extended by the Prospective Physical Activity, Sitting, and Sleep consortium, which suggested a subdivision of the 24-hour physical behavior construct into 3 behaviors by applying different dimensions [4], meaning that each behavior covers aspects of biological (ie, sleep or awake), postural (eg, lying, sitting, and upright), and intensity (eg, light, moderate, and vigorous) dimensions. Therefore, the differentiation among PA, SB, and sleep in terms of the 24-HAC model requires valid and simultaneous assessments of all 3 dimensions (ie, biological state, posture or activity type, and intensity; Multimedia Appendix 1, Table S1 [5-226]) under real-life conditions. Rigorous validation studies performed in a free-living environment are necessary to accurately predict the performance of a device and algorithm under real-life conditions.

### Wearable Technology and Validation

As technical opportunities have evolved rapidly during the past decades, wearables (ie, body-worn devices such as accelerometers, smartwatches, pedometers, or fitness trackers) have become a leading fitness trend, with an estimated US $95 billion industry [227], which is still growing. Moreover, a considerable number of research studies integrated device-based methods to capture physical behavior data, and first discussions have already come up on whether it is *prime time* for wearables with scientific validation to be a global physical behavior surveillance methodology [228,229]. However, the application of wearables in studies that assess health-related questions presents several methodological and practical challenges. For example, strategies for data processing, monitoring protocols, assessment limitations (eg, muscle-strengthening exercises), and quality criteria such as validity need to be taken into account [230].

An important test quality criterion is the concept of validity, which represents a fundamental criterion for evaluating the quality of an instrument, referring to the degree to which it truly measures the construct it targets [231]. Regarding the 24-hour physical behavior cycle, researchers are commonly interested in criterion-referenced validity as their assessed outcome parameters are highly objective [232]. Although (or because of) the number of validation studies has increased over the past years, there is high heterogeneity across published protocols and used measurement methods, which severely limits valid device comparisons [233]. Thus, suggestions for standardized validation procedures have received increasing attention in the scientific community [233-235].

### Standardized Protocols and Validation Framework

There have been several attempts in this direction, as collaborations such as the INTERLIVE network have already started developing standardized protocols to validate consumer wearables for steps [233] and heart rate [236]. Furthermore, Keadle et al [234] introduced a stage process framework of validity to facilitate the development and validation of processing methods for assessing physical behavior using wearables. This framework contains 5 validation phases with increasing levels, starting from device manufacturing and culminating with application in health studies. Validation studies should be implemented following mechanical testing (phase 0) and calibration testing (phase 1). Here, a fixed and semistructured evaluation under laboratory conditions (phase 2) should be applied, followed by an evaluation under real-life conditions (phase 3) [234]. The validation of devices should pass through all these stages before the respective device can be used in health research studies (phase 4). As there is a nonnegligible difference in error rates between laboratory and real-life conditions [233], a wide array of activities of daily living should be captured and compared under real-life conditions. It also needs to be taken into account that participants are instructed to perform specific activities under laboratory conditions, which may result in unnaturally performed activities (eg, the Hawthorne effect) [237]. Hence, the quantification of measurement error is essential to be defined in an unconstrained free-living environment, and wearables’ outcomes should be compared with a reference measure such as video recordings or the doubly labeled water method, depending on the outcome parameter of choice. Overall, the aim should be the realization of standardized validation protocols to be embedded in a framework [233,234], which may have positive implications for all stakeholders such as manufacturers, scientists, and consumers. The results of validation studies are helpful to disabuse consumers and can assist researchers in study design when selecting an appropriate wearable device for the respective question or questions to be answered [238,239].

### Objectives

Although validated devices are a prerequisite for proper research and validation frameworks have been proposed, to the best of our knowledge, no previous review has systematically evaluated the characteristics and quality of free-living validation studies. This review focuses on the following purposes. First, as our main purpose, we aimed to raise researchers’ and consumers’ attention to the quality of published validation protocols while aiming to identify and compare specific consistencies and inconsistencies between validation protocols. To evaluate the quality of the studies, we followed core principles, recommendations, and expert statements [232-234,240] with published quality criteria (eg, study duration, number of included participants, selection of criterion measures, and data synchronization). Second, we aimed to provide a comprehensive and historical overview of which wearable devices have been validated for which purpose and whether they show promise for use in further studies.

## Methods

This study followed the PRISMA (Preferred Reporting Items for Systematic Reviews and Meta-Analyses) reporting guidelines (Multimedia Appendix 1, Table S2 [5-226]) [241].

### Search Strategy and Study Selection

Three separate search strings were combined: terms for validation analyses, 24-hour physical behavior constructs, and wearables. An a priori pilot search was conducted to optimize the final term (Multimedia Appendix 1, Table S3 [5-226]). Publications from 1970 to December 2020 were searched using the following databases: EBSCOhost, IEEE Xplore, PubMed, Scopus, and Web of Science. We reran the search in July 2021 to check for updates and checked the reference lists of included studies for publications that met the inclusion criteria.

All articles were imported to the Citavi library (Citavi 6.8; Swiss Academic Software GmbH). After removing all duplicates, the study selection process included 3 screening phases for eligibility. In the first phase, 2 reviewers (MG and RN) independently screened the titles of the publications. Articles were excluded only if both reviewers categorized them as not eligible for review purposes. In the second phase, 2 reviewers (MG and RN) independently screened and reviewed the abstracts of the publications. Discrepancies in screening were resolved by consulting with a third reviewer (BvHM). Finally, in the third phase, the full texts of the remaining articles were assessed for eligibility by 7 members of the author’s team (MG, RN, DD, KS, SS, IT, and BvHM). Each article was independently screened by at least two reviewers. Discrepancies in screening were resolved through discussion until a consensus was reached. The reviewers were not blinded to the author or journal information.

### Inclusion and Exclusion Criteria

On the basis of the population, intervention, comparison, and outcome principle [242], we included peer-reviewed English-language publications that met the criteria described in the following sections.

#### Population

Participants were adults and older adults aged ≥18 years, regardless of health conditions. Studies that specifically targeted adults and populations of older adults (aged ≥18 years) were excluded.

#### Intervention

Any wearable validation study in which at least one part of the study was conducted under free-living (naturalistic or real-life) conditions (eg, at participants’ homes or schools and without instructions on when to start or stop a particular activity) was included. Studies in which the protocol was conducted under laboratory conditions were excluded.

#### Control or Comparison

We included only studies where a criterion measure was described (eg, observation or wearable devices) and excluded all studies where no criterion measure was described (eg, comparison between 2 devices without indicating a criterion measure).

#### Outcomes

Studies were included in which the wearable outcome or outcomes could be classified into at least one dimension of the 24-hour physical behavior construct (ie, biological state, posture or activity type, or intensity [4]; Multimedia Appendix 1, Table S1 [5-226]). We excluded studies in which the outcome did not distinguish among sleep-wake states; intensities; or posture or activity types such as sleep quality, gait analyses, or heart rate parameters.

### Data Extraction and Synthesis

Data extraction and synthesis were conducted independently by 2 authors (MG, RN, DD, KW, SS, or IT). Discrepancies were discussed until a consensus was reached. The following study details were extracted: author, year, location, population information (sample size, mean age of participants, percentage of females, and ethnicity), measurement period, validated wearable device (wearing position, software, epoch length, and cutoff point or algorithm), dimensions of the 24-hour physical behavior construct (biological state, intensity, and posture or activity type), validated outcome, criterion measure, statistical analyses for validation purposes, conclusion, and funding information. Given the wide range of study protocols in terms of varying conditions (eg, wear location, measurement duration, sample size, statistical analyses, or criterion measure), we conducted a narrative synthesis based on the reported results or conclusions. The data synthesis focused on the purpose (ie, whether the included wearables showed promise for use in further studies). In particular, we classified the studies as moderate to strong validity, mixed results, and poor or weak validity.

### Quality Assessment

The risk of bias for each article was evaluated using the Quality Assessment of Diagnostic Accuracy Studies-2 (QUADAS-2) tool [240]. The tool comprises 4 different domains (ie, patient selection, index measure, criterion measure, and flow and timing). Following the QUADAS-2 guidelines, we selected a set of signaling questions for each domain and added questions modified from the QUADAS-2 background document based on core principles, recommendations, and expert statements for validation studies [232-234,240]. The risk of bias assessment was independently conducted by at least two authors. Discrepancies were discussed until a consensus was reached. The study quality was evaluated at the domain level; that is, if all signaling questions for a domain were answered *yes*, then the risk of bias was deemed to be *low*. If any signaling question was answered *no*, then the risk of bias was deemed to be *high*. The *unclear* category was only used when insufficient data were reported for evaluation. On the basis of domain-level ratings, we created a decision tree to evaluate the overall study quality as *low risk*, *some concerns,* or *high risk* (Multimedia Appendix 1, Figure S1 [5-226]).

## Results

### Overview

**Figure 1:**
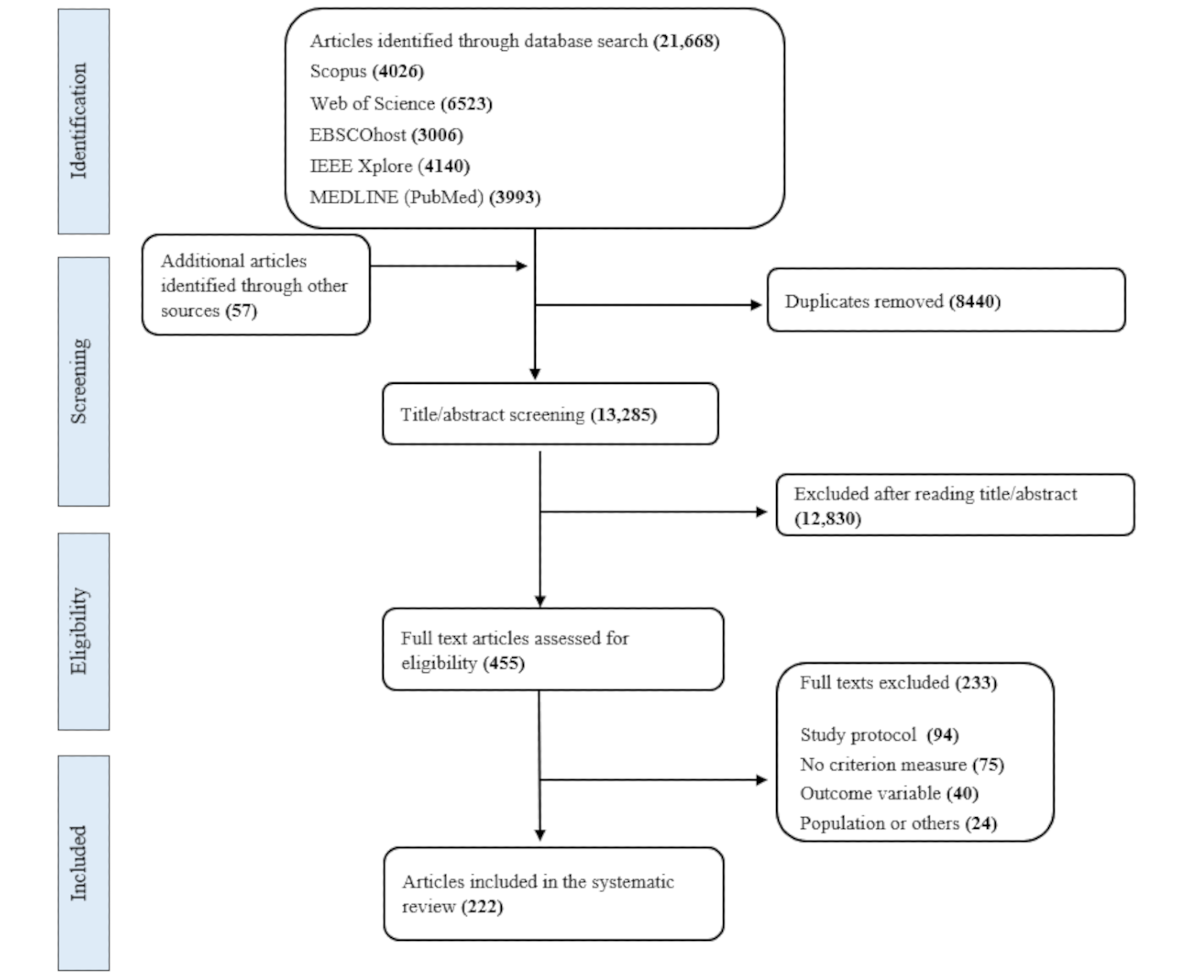
PRISMA (Preferred Reporting Items for Systematic Reviews and Meta-Analyses) flowchart illustrating the literature search and screening process.

The search resulted in 13,285 unique records, with 222 (1.67%) publications being included [5-226] (Figure 1). Most studies (208/222, 93.7%) validated an outcome from one dimension, whereas few (14/222, 6.3%) studies validated outcomes from 2 different dimensions (ie, intensity and posture or activity type or intensity and biological state) at the same time during a study protocol. Only 0.5% (1/222) of studies included outcomes from all the 3 dimensions. In particular, of all the 237 identified outcomes, 153 (64.6%) were classified into the intensity dimension, 38 (16%) were classified into the posture or activity type dimension, and 47 (21.2%) were classified into the biological state dimension.

### Participant and Study Characteristics

Of the included studies, 84.2% (187/222) were published within the past decade (in or after 2011), indicating the increasing use of wearable technologies for physical behavior measurement (Table 1); 93.2% (207/222) were conducted in wealthier high-income countries in North America, Europe, or Australia and Oceania. The number of participants ranged between 1 and 3752, although most studies (113/222, 50.9%) recruited between 20 and 50 participants. The mean age of the participant samples ranged between young (18.0, SD 0.6 years) and older adults (86.4, SD 6.0 years). In most studies, the mean age of the sample was between 18 and 64 years, and the proportion of female participants ranged from 26% to 74% (144/222, 64.8%). Healthy participants were recruited in 79.7% (177/222) of all studies, whereas 20.7% (46/222) of all studies included participants with different physical and mental health restrictions such as cardiometabolic diseases or chronic heart failure in 2.7% (6/222), chronic obstructive pulmonary disease in 2.7% (6/222), stroke in 2.3% (5/222), insomnia in 2.3% (5/222), or intellectual and visual disabilities in 0.9% (2/222). Information about the participants’ ethnicity was reported in 14.9% (33/222) of all studies. The conceptualization of study protocols regarding measurement duration varied between approximately 30 minutes and up to several weeks. The study duration of ≤1 day was predominantly for studies that focused on posture or activity-type outcomes (ie, in 18/36, 50% of the included studies). Most studies (205/222, 92.3%) conducted statistical analyses at the person or study level (eg, correlations, 2-tailed *t* tests, and repeated-measures ANOVA). Some studies (21/222, 9.5%) conducted both person or study-level analyses and epoch-by-epoch comparisons (eg, sensitivity and specificity). Approximately 10.4% (23/222) of studies reported that the manufacturer was involved in the study funding or provided devices for validation purposes. No funding information was reported by 16.2% (36/222) of the studies, whereas the remaining studies (164/222, 73.9%) indicated that funding was independent of manufacturer companies. Detailed data extraction is reported as a supplement (Multimedia Appendix 1, Table S4 [5-226]).

**Table 1:** Summary of data extraction: participant and study characteristics (N=237).

Category			Total, n (%)		Biological state (n=47), n (%)		Posture or activity type (n=37), n (%)		Intensity (n=153), n (%)
**Publication year**									
	Before or in 1999	7 (3)		1 (2.1)		3 (8.1)		3 (2)	
	2000-2010	28 (11.8)		4 (8.5)		3 (8.1)		21 (13.7)	
	After or in 2011	187 (78.9)		42 (89.4)		31 (83.8)		129 (84.3)	
**Study location^a^**									
	Africa	1 (0.4)		—^b^		—		1 (0.7)	
	Asia	17 (7.2)		5 (10.6)		2 (5.4)		10 (6.5)	
	Europe	95 (40.1)		14 (29.8)		21 (57)		69 (45.1)	
	North America	92 (38.8)		21 (44.7)		10 (27)		65 (42.5)	
	Australia or Oceania	16 (6.8)		7 (14.9)		4 (10.8)		7 (4.6)	
**Number of participants**									
	≤19	71 (30)		12 (25.5)		22 (59.5)		40 (26.1)	
	20-50	113 (47.7)		23 (48.9)		13 (35.1)		86 (56.2)	
	≥51	38 (16)		12 (25.5)		2 (5.4)		27 (17.6)	
**Age (years; mean age)^c^**									
	18-64	174 (73.4)		36 (76.6)		28 (75.7)		122 (79.7)	
	≥65	44 (18.6)		10 (21.3)		7 (18.9)		31 (20.3)	
**Sex (female; %)^d^**									
	0-25	33 (13.9)		6 (12.8)		6 (16.2)		22 (14.4)	
	26-74	144 (60.8)		36 (76.6)		22 (59.5)		99 (64.7)	
	75-100	35 (14.8)		5 (10.6)		8 (21.6)		23 (15)	
**Measurement duration (days)^e^**									
	≤1	69 (29.1)		17 (36.2)		19 (51.4)		39 (25.5)	
	2-6	50 (21.1)		15 (31.9)		7 (18.9)		33 (21.6)	
	≥7	101 (42.6)		15 (31.9)		9 (24.3)		80 (52.3)	
**Criterion measure**									
	Doubly labeled water	42 (17.7)		—		—		42 (27.5)	
	Heart telemetry	—		—		—		—	
	Indirect calorimetry	4 (1.7)		—		—		4 (2.6)	
	Observation (direct)	7 (3)		—		4 (10.8)		3 (2)	
	Observation (images)	2 (0.8)		—		2 (5.4)		—	
	Observation (video)	14 (5.9)		—		11 (29.7)		5 (3.3)	
	Polysomnography	24 (10.1)		24 (51.1)		—		—	
	Questionnaire or diary	16 (6.8)		6 (12.8)		4 (10.8)		6 (3.9)	
	Wearable	113 (47.7)		14 (29.8)		16 (43.2)		94 (61.4)	
	EEG^f^ or Zmachine	4 (1.7)		3 (6.4)		—		—	
**Statistical analyses**									
	Epoch-by-epoch	33 (13.9)		13 (27.7)		15 (40.5)		8 (5.2)	
	Person or study level	208 (87.8)		44 (93.6)		28 (75.7)		150 (98)	

^a^One study did not report the study location.

^b^Not available.

^c^A total of 5 studies were not included in the summary statistics because of the lack of age information. One study was counted twice as it included 2 different age groups.

^d^A total of 10 studies was not included in the summary statistics because of the lack of sex information.

^e^A total of 2 studies was not included in the summary statistics because of the lack of measurement duration information.

^f^EEG: electroencephalogram.

### Wearables

We identified 163 different wearables from 82 companies, of which 61 (37.4%) were classified as research-grade devices and 102 (62.6%) were classified as commercial-grade devices. The types of wearables varied across uniaxial, biaxial, or triaxial accelerometers and pedometers. On the basis of our narrative data synthesis, we ranked 26.4% (122/463 of results or conclusions as moderate to strong validity, 48.8% (226/463) as mixed validity, and 25% (115/463) as poor or weak validity (Multimedia Appendix, Table S5 [5-226]). In relation to other types of wearables, triaxial accelerometers were used in 70.5% (167/237) of all included studies. Detailed technical information for each wearable device is available as a supplement (Multimedia Appendix, Table S6 [5-226]). Of the 163 different wearables, 96 (58.9%) were validated only once. ActiGraph GT3X/GT3X+ (36/163, 22.1%), Fitbit Flex (20/163, 12%), and ActivPAL (12/163, 7.4%) were used most often in the included validation studies. Of all the 222 reviewed articles, 78 (35.1%) studies included different types of wearables, and 26 (11.7%) studies included different wearing positions to enable inter- and intradevice comparisons (Table 2). The variation of different sensor models within a study protocol ranged from 1 to 12 different wearables. In particular, 64.9% (144/222) of all studies included one model of wearable, 20.3% (45/222) included 2 different models of wearables, and 14.9% (33/222) included ≥3 different models of wearables. We identified 11 different validated outcomes. Of all reported outcomes, 51.3% (164/320) represented continuous parameters such as steps, energy expenditure, or counts, whereas 48.8% (156/320) represented categorical outcomes such as sleep time, time spent in light PA, or time spent in moderate to vigorous PA. Approximately 18% (40/222) of studies validated 2 different outcomes such as steps and energy expenditure during a study protocol. More than half of the studies (125/237, 52.7%) validated one type of wearable device in a single wearing position. We identified 13 different wearing positions. The wrist and hip or waist positions were used most often for validation purposes. In 50% (111/222) of all studies, the authors provided information about the software application used for data preprocessing.

**Table 2:** Summary of data extraction: wearables (N=237).

Category			Total, n (%)		Biological state (n=47), n (%)		Posture or activity type (n=37), n (%)		Intensity (n=153), n (%)
**Type**									
	Uniaxial accelerometer	44 (10.8)		13 (14.4)		3 (6.4)		29 (9.2)	
	Biaxial accelerometer	28 (6.9)		1 (1.1)		2 (4.3)		26 (8.3)	
	Triaxial accelerometer	286 (70.1)		72 (80)		40 (85.1)		212 (67.5)	
	Pedometer	30 (7.4)		2 (2.2)		—^a^		30 (9.6)	
	Unclear	20 (4.9)		2 (2.2)		2 (4.3)		17 (5.4)	
**Outcome**									
	Sleep time	42 (13.1)		42 (89.4)		—		—	
	Sleep-wake metrics	5 (1.6)		5 (10.6)		—		—	
	Different postures or types	29 (9.1)		—		30 (73.2)		—	
	Sit-to-stand transitions	5 (1.6)		—		5 (12.2)		—	
	Time in sedentary behavior	32 (10)		—		6 (14.6)		26 (11.2)	
	Time in light physical activity	14 (4.4)		—		—		14 (6)	
	Time in moderate to vigorous physical activity	33 (10.3)		—		—		33 (14.2)	
	Time in physical activity	6 (1.9)		—		—		6 (2.6)	
	Energy expenditure	72 (22.5)		—		—		72 (30.9)	
	Steps	75 (23.4)		—		—		75 (32.2)	
	Counts	7 (2.2)		—		—		7 (3)	
**Wear position^b^**									
	Ankle	11 (2.4)		1 (1)		1 (1.6)		9 (2.6)	
	Backpack, pocket, and bra	20 (4.3)		—		2 (3.1)		19 (5.6)	
	Chest	15 (3.3)		1 (1)		3 (4.7)		11 (3.2)	
	Foot	3 (0.7)		1 (1)		—		2 (0.6)	
	Hip and waist	148 (32.2)		7 (7.1)		23 (35.9)		124 (36.4)	
	Leg	3 (0.7)		—		3 (4.7)		—	
	Lower back	12 (2.6)		1 (1)		3 (4.7)		10 (2.9)	
	Neck	3 (0.7)		—		—		3 (0.9)	
	Thigh	28 (6.1)		2 (2)		15 (23.4)		13 (3.8)	
	Torso	1 (0.2)		—		—		1 (0.3)	
	Trunk	3 (0.7)		—		3 (4.7)		—	
	Upper arm	12 (2.6)		1 (1)		1 (1.6)		10 (2.9)	
	Wrist	201 (43.7)		83 (84.7)		10 (15.6)		139 (40.8)	

^a^Not available.

^b^One study did not report any information about the sensor wearing position and one study did not specify the information about the sensor wearing position. If studies included multiple devices or different wearing positions, we counted each device and wearing position separately.

### Study Quality

**Figure 2:**
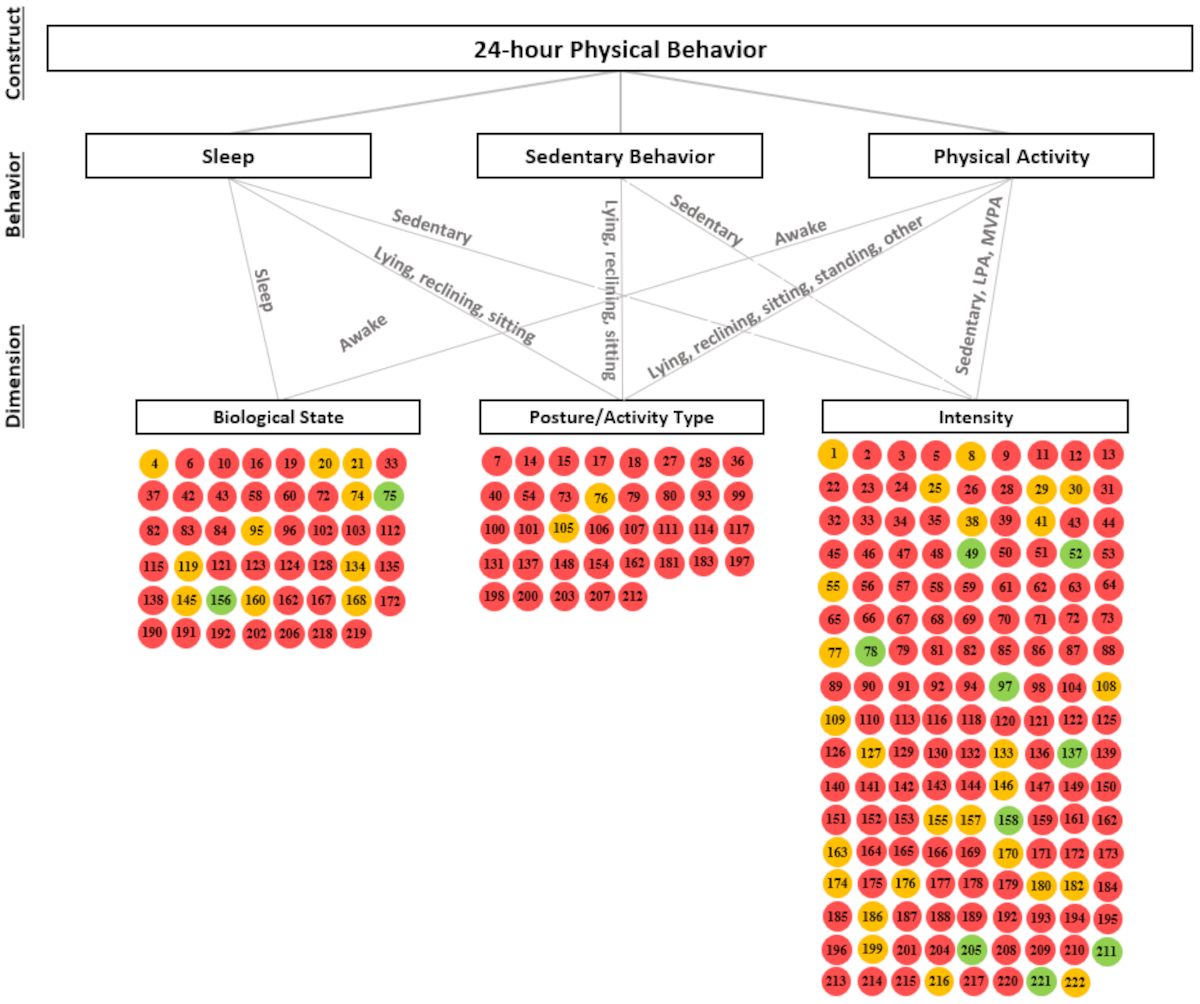
The overall risk of bias classification separated by different dimensions of the 24-hour physical behavior construct. The number within the circles represents the study number, as listed in the full data extraction. Studies with a green circle were evaluated as low risk, the orange circle represents the quality with some concerns, and red circles represent high risk. LPA: light physical activity; MVPA: moderate to vigorous physical activity.

In total, we included 9 signaling questions as quality criteria to evaluate the risk of bias. The percentage of studies that met the criteria ranged from 38.7% (92/238) to 92% (219/238; Table 3). On average, 5.2 (SD 1.41) of 9 questions were answered with *yes* (ie, meeting the criteria). Studies validating a biological state, intensity, or posture or activity type outcome met on average 4.6 (SD 1.23), 5.5 (SD 1.36), and 4.9 (SD 1.51) out of 9 questions with *yes* (ie, no risk of bias), respectively. We evaluated whether the reference standard was the appropriate gold standard as a central criterion for evaluating overall study quality. In 38.4% (91/237) of all studies, the reference standard was equivalent to the suggested criterion measures [234]. Wearables were the most frequently selected reference criterion in 50.5% (112/222) of the studies. On the basis of our classification tree to evaluate the overall study quality (Multimedia Appendix 1, Figure S1 and Table S7 [5-226]), 4.6% (11/237) of studies were classified as *low risk*. Furthermore, 16% (38/237) of studies were classified as having *some concerns*, and 72.9% (173/237) of studies were classified as *high risk*. To provide an overview of the study quality, Figure 2 illustrates the overall study quality on a study level, separated by each dimension of the 24-hour physical behavior construct.

**Table 3:** Criteria for the risk of bias assessment and the percentage of studies meeting these criteria (N=237).

Criteria items		Studies meeting criterion, n (%)			
		Total	Biological state (n=47)	Posture or activity type (n=37)	Intensity (n=153)
**Domain 1: patient selection or study design**					
	Was the study conducted in different free-living settings (eg, work or home)?	174 (91.6)^a^	N/A^b^	28 (75.7)	146 (95.4)
	Did the study take place for at least 2 days?	156 (65.8)	28 (59.6)	16 (43.2)	112 (73.2)
	Did the study provide any information about the inclusion and exclusion criteria of the recruiting process?	165 (69.6)	34 (72.3)	25 (67.6)	106 (69.3)
	Did the study include a sample of at least 20 participants?	163 (68.8)	36 (76.6)	15 (40.5)	112 (73.2)
**Domain 2** **: index measure**					
	Was the algorithm of the validated outcome reported (ie, formula) or at least further information cited?	97 (40.9)	23 (48.9)	18 (48.6)	56 (36.6)
	Did the participants wear the wearable for at least 8 hours per day?	107 (56.3)^a^	N/A	15 (40.5)	92 (60.1)
**Domain 3: criterion measure**					
	Is the selected reference the gold standard?	91 (38.4)	23 (48.9)	14 (37.8)	54 (35.3)
**Domain 4: flow and timing**					
	Did the authors provide any information about data synchronization?	75 (41.4)^c^	24 (51.1)	22 (59.5)	29 (19)
	Were all participants included in the analyses or were any exclusion reasons provided?	218 (92.4)^d^	45 (95.7)	31 (83.8)	142 (92.8)

^a^Only relevant for 190 studies.

^b^N/A: not applicable.

^c^Only relevant for 181 studies.

^d^Only relevant for 236 studies.

## Discussion

### Principal Findings

We evaluated the characteristics and quality of free-living validation studies in which at least one dimension of the 24-hour physical behavior construct (ie, biological state, posture or activity type, and intensity [1,4]) was assessed using wearables and validated against a criterion measure. In summary, the validation of biological state and posture or activity-type outcomes was rare, and almost all of the 163 different types of research- and commercial-grade wearables were validated for only one aspect of the 24-hour physical behavior construct (ie, intensity outcomes). Compared with the selected quality criteria for studies under free-living conditions that are in line with published core principles, recommendations, and expert statements [234-236], most of the reviewed protocols failed to meet the criteria; however, only a few of the evaluated studies were overall ranked with *low risk* of bias or with *some concerns*. Therefore, more high-quality validation studies with adults and older adults under real-life conditions are needed. According to the framework of wearable validation studies [234], the aim of phase 3 studies is to validate device outcomes under real-life conditions against appropriate reference measures.

### Criterion Measure

Our evaluation of the most central category physical behavior *criterion measure* followed Keadle et al [234]; for example, physiological outcomes (eg, activity energy expenditure) are recommended to be validated against indirect calorimetry or doubly labeled water, behavioral criterion measures (eg, step count and postures) are recommended to be validated against video-recorded direct observation [234], and the recommended criterion measure for differentiation between sleep and wake patterns is polysomnography [243,244]. Notably, only 40.9% (91/222) of the reviewed studies used the recommended gold standard. Primarily, research-grade devices served as criterion measures, which is highly critical as there is no evidence that wearables can serve as a basis for validating other wearables and offer a high risk of bias regarding criterion validity [233,245,246].

### Study Duration

Optimally, study protocols take place over a 24-hour period over multiple days, thus covering a wide range of representative habitual activities [233,234]. This recommended criterion was covered by 2 signaling questions. First, we evaluated whether data collection was not restricted to one particular setting (eg, at work or at home), which was met by nearly all the reviewed studies. Second, as it is almost not feasible to collect data over several days for criterion measures such as video recording [233,234], we specified at least 2 days for a *low-risk* classification. Two-thirds of the reviewed studies met this criterion. However, we identified a considerable number of studies (69/222, 31.1%) that collected data over a short period (≤1 day). The risk of bias might have been present as the setting was restricted to a specific environment (eg, at work), thus limiting the ability to capture a wide range of habitual behaviors. Moreover, reactivity is a serious issue that reveals a potential error source when collecting data from wearables. Researchers expected that reactivity would be a time issue, implying that participants may change their behavior at the beginning of the monitoring period but return to a more stable pattern later [247,248]. Similar effects have been observed in sleep laboratories using polysomnographic monitoring [249].

### Study Population

Ideally, the validity of wearables can be generalized to a wide range of diverse samples (eg, age, sex, ethnicity, and health condition) [234,250]. While focusing on adults and older adults (aged ≥18 years), this review revealed that 78.4% (174/222) of the studies included samples between 18 and 64 years of age, whereas there was a lack of studies that included older adults. Critically, most devices (99/163, 60.7%) were validated only once. According to the recommended principle, validation study protocols should include either a variety of cohorts within a single study or a series of studies with different participant characteristics [233,234,251]. For example, we could only identify 20.3% (45/222) of studies that included samples with restricted health conditions. As a practical implication, a given wearable device might be valid for healthy adults and older adults but not for those with health restrictions [232]. A solution might be to recruit a larger sample size, which would enable a higher intersubject variability, or to conduct a series of validation studies with varying participant characteristics. Optimally, sample size calculations ensure adequate power for validation purposes [233,252]. Finally, although challenging because of data protection guidelines, we recommend reporting information about ethnicity (reported in 32/222, 14.4% of studies) whenever possible and providing detailed information about inclusion and exclusion criteria regarding the recruiting process and for statistical analyses.

### Wearing Position and Types of Wearables

To enable a comparison between different wearables or wearing positions, researchers may simultaneously collect data from multiple sensors or different wearing positions [234,251]. Most of the reviewed studies did not include multiple wearables (eg, research and commercial grade) and did not capture data from the validated devices at different wearing positions (eg, hip or waist, wrist, and thigh). Depending on the primary outcome of interest, the recommendations of where to place the wearable device may vary. For example, to assess sleep-wake patterns, wrist-worn devices may optimize the recording of small movements that occur at the distal extremities when an individual is supine [245,253]. For example, Fairclough et al [254] reported that wrist placement promotes superior compliance than that of the hip position. In contrast, if researchers are interested in differentiating between body postures (eg, sitting vs standing), the thigh might be the position of choice because of the option of wearing the device under clothing to accurately assess intensity and posture or activity types [4]. However, only 1.8% (4/222) of studies validated posture or activity-type outcomes using thigh-worn devices. Future validation studies are needed with multiple devices and different wearing positions to increase comparability and to inform end users of which device to use and where to place it [250]. In addition, future signal analytical research purposes might be valuable in terms of extracting different outcomes from a single wearing position. Moreover, different types of wearables (ie, pedometers and uniaxial, biaxial, and triaxial accelerometers) have been validated. Researchers should be aware that the different types of devices have different technical requirements. For example, uniaxial accelerometers measure acceleration in 1 direction, whereas triaxial accelerometers measure acceleration in 3 directions. Thus, triaxial accelerometers provide more information, which might be helpful in developing further algorithms.

### Synchronization, Transparency, and Statistical Analyses

We evaluated whether the studies reported data synchronization, wear time, the algorithm of the validated outcome, and data analyses. As less than half (75/222, 33.8%) of all included studies reported information about the synchronization process between index and criterion measures, potentially introducing errors and biasing results, we suggest future research endeavors to apply time-stamped solutions such as asking participants to perform 3 vertical jumps at the beginning and the end of the measurement [233]. Following practical consideration when applying wearables [255,256], a large number of studies defined a valid day if ≥10 hours of wear time during waking hours were captured. We set the quality criterion to ≥8 hours per day, revealing that 56% (107/190) of studies considered the wear time criteria for a valid day. Capturing shorter periods may increase the risk of bias as less time is available to assess the data in different settings (eg, at home or at work).

A critical aspect from the perspective of transparency is the presentation of algorithms. Only 43.7% (97/222) of studies reported the algorithm or at least cited further information on the validated outcomes. In particular, no information about the used algorithms was provided in studies in which a commercial-grade device was validated. At this point, researchers often do not have access to the raw data of commercial-grade wearables or the *black box* algorithms. Moreover, companies can update wearables’ firmware or algorithms at any time, which hinders comparability [257]. In addition, the pace at which technology is evolving in optimizing algorithms far exceeds the pace of published validation research [238]. Open-source methods that are more flexible in using algorithms for different devices are needed [233,234].

A quality criterion for the used statistical analyses was not set because of the lack of consistent statistical guidelines for reporting the validity of an activity monitor. Most of the reviewed studies used traditional statistical tests of differences such as *t* tests or ANOVAs. Optimally and in line with recently published suggestions, researchers should integrate different analytical approaches such as combining traditional analyses with equivalence testing and including epoch-by-epoch comparisons whenever possible [234,258].

### Limitations

Some points merit further discussion. First, the evaluation of study quality was based on self-selected criteria. In particular, we selected the QUADAS-2 [240] tool and added further signaling questions in line with core principles, recommendations, and expert statements [232-234]. However, as we are not aware of any further quality tools and signaling questions for wearable validation purposes, our selected criteria can serve as a starting point for future systematic reviews that focus on the study quality of wearable technology under free-living conditions. Second, our included validation studies were published between 1987 and 2021. Given the rapid development of wearable technologies and the increasing availability of different research and commercial-grade devices, quality standards have been developed. Thus, when interpreting the study protocols, the time during which the study was conducted should be considered. Third, our review focused on the quality of study protocols. However, we did not take into account further important considerations when using wearables, such as wear or nonwear time algorithms, costs of the monitor, or time of data processing [35,250]. Fourth, our findings were limited to our search strategy; thus, we may have missed further validation studies. However, we applied backward and forward citation searches through the reference lists of the included studies to identify articles that may not have appeared in our search. Finally, this systematic review was limited to articles published in the English language.

### Conclusions

Currently, there is a wealth of research on commercial-grade wearables; however, the quality of published validation protocols in adults had not been assessed thus far. However, this is a critical step to enable both researchers and consumers to make guided decisions on which study to rely on and which device to use. To this end, our review unraveled that most validation studies did not meet the recommended quality principles [233,250]. Primarily, there is a lack of validation studies with gold standard reference measures such as video recording, polysomnography, or the doubly labeled water method. Moreover, most devices were validated only once and focused predominantly on intensity measure outcomes. Given the rising interest in the 24-hour physical behavior construct in health research, the next generation of validation studies should consider the validity of >1 aspect of the 24-hour physical behavior construct during a study protocol or to conduct a series of studies. Thus, we conclude that standardized protocols for free-living validation embedded in a framework [234] are urgently needed to inform and guide stakeholders (eg, manufacturers, scientists, and consumers) in (1) selecting wearables for self-tracking purposes, (2) applying wearables in health studies, and (3) fostering innovation to achieve improved validity.

## Appendix

Multimedia Appendix 1 Characteristics of 24-hour physical behavior, PRISMA (Preferred Reporting Items for Systematic Reviews and Meta-Analyses) checklist, search terms used in databases, classification tree for the judgment of overall study quality, data extraction, the validity of wearables, an overview of wearables used in validation studies, and risk of bias for the included studies.

## Abbreviations

24-HAC: 24-hour activity cycle
PA: physical activity
PRISMA: Preferred Reporting Items for Systematic Reviews and Meta-Analyses
QUADAS-2: Quality Assessment of Diagnostic Accuracy Studies-2
SB: sedentary behavior
