# Supplementary material for: Quality Evaluation of Free-living Validation Studies for the Assessment of 24-Hour Physical Behavior in Adults via Wearables: Systematic Review
Source: JMIR Mhealth Uhealth. 2022 Jun 9;10(6):e36377. doi: 10.2196/36377 (PMC9227659; doi:10.2196/36377)
Supplement: Multimedia Appendix 1 [file mhealth_v10i6e36377_app1.pdf]

Table S1. Characteristics of 24-hour physical behavior.

BEHAVIOR	SLEEP	PHYSICAL ACTIVITY	SEDENTARY BEHAVIOR
**Definition**	A naturally recurring and easily reversible state that is characterized by reduced or absent consciousness, perceptual disengagement, immobility, and the adoption of a characteristic sleeping posture	Any voluntary movement produced by skeletal muscles that results in energy expenditure	Any waking behavior characterized by an energy expenditure of 1.5 metabolic equivalents (METs; 1 MET = energy expenditure in rest), while in a sitting, reclining, or lying posture
**Intensity category** (Individual is sedentary or conducting LPA or MVPA – e.g. differentiated by MET-thresholds)	Sedentary (e.g., ~ 1 MET)	Sedentary (≤ 1.5 MET), light physical activity (LPA) (1.51-2.99 MET), moderate-to-vigorous physical activity (MVPA) (≥ 3 MET)	Sedentary (≤ 1.5 MET)
**Body Posture and/or movement** (postural location – e.g., differentiated by specific activity types)	Sitting, reclining, lying	Standing, cycling, walking running, wheelchair driving	Sitting, reclining, lying
**Activity type**	e.g., sleep at night, nap	e.g. vacuuming, commuting, driving a car	e.g. desk work, watching TV
**Biological State** (condition asleep or awake)	Asleep	Awake	Awake
**Domain**	At home, not at home	Work, home, leisure, transportation	Work, home, leisure, transportation
**Bout Length**	Short, moderate, Long	Short, moderate, Long	Short, moderate, Long
**Outcomes (examples)**	Sleep time, sleep-wake metrics, time awake	Energy expenditure, steps, time spent in different intensity categories, Time spent in standing or walking	Sedentary time, number of sedentary bouts

Table S2. PRISMA checklist.

Section and Topic	Item #	Checklist item	Location where item is reported
**TITLE**			
Title	1	Identify the report as a systematic review.	p.1
**ABSTRACT**			
Abstract	2	See the PRISMA 2020 for Abstracts checklist.	p.2-3
**INTRODUCTION**			
Rationale	3	Describe the rationale for the review in the context of existing knowledge.	p.4-6
Objectives	4	Provide an explicit statement of the objective(s) or question(s) the review addresses.	p.7
**METHODS**			
Eligibility criteria	5	Specify the inclusion and exclusion criteria for the review and how studies were grouped for the syntheses.	p.8-9
Information sources	6	Specify all databases, registers, websites, organisations, reference lists and other sources searched or consulted to identify studies. Specify the date when each source was last searched or consulted.	p.8
Search strategy	7	Present the full search strategies for all databases, registers and websites, including any filters and limits used.	p.8; Table S3
Selection process	8	Specify the methods used to decide whether a study met the inclusion criteria of the review, including how many reviewers screened each record and each report retrieved, whether they worked independently, and if applicable, details of automation tools used in the process.	p.8
Data collection process	9	Specify the methods used to collect data from reports, including how many reviewers collected data from each report, whether they worked independently, any processes for obtaining or confirming data from study investigators, and if applicable, details of automation tools used in the process.	p.9-10
Data items	10a	List and define all outcomes for which data were sought. Specify whether all results that were compatible with each outcome domain in each study were sought (e.g. for all measures, time points, analyses), and if not, the methods used to decide which results to collect.	p.9-10
	10b	List and define all other variables for which data were sought (e.g. participant and intervention characteristics, funding sources). Describe any assumptions made about any missing or unclear information.	p.9-10
Study risk of bias assessment	11	Specify the methods used to assess risk of bias in the included studies, including details of the tool(s) used, how many reviewers assessed each study and whether they worked independently, and if applicable, details of automation tools used in the process.	p.10
Effect measures	12	Specify for each outcome the effect measure(s) (e.g. risk ratio, mean difference) used in the synthesis or presentation of results.	NA
Synthesis methods	13a	Describe the processes used to decide which studies were eligible for each synthesis (e.g. tabulating the study intervention characteristics and comparing against the planned groups for each synthesis (item #5)).	p.9-10
	13b	Describe any methods required to prepare the data for presentation or synthesis, such as handling of missing summary statistics, or data conversions.	p.9-10
	13c	Describe any methods used to tabulate or visually display results of individual studies and syntheses.	NA

Section and Topic	Item #	Checklist item	Location where item is reported
	13d	Describe any methods used to synthesize results and provide a rationale for the choice(s). If meta-analysis was performed, describe the model(s), method(s) to identify the presence and extent of statistical heterogeneity, and software package(s) used.	NA
	13e	Describe any methods used to explore possible causes of heterogeneity among study results (e.g. subgroup analysis, meta-regression).	NA
	13f	Describe any sensitivity analyses conducted to assess robustness of the synthesized results.	NA
Reporting bias assessment	14	Describe any methods used to assess risk of bias due to missing results in a synthesis (arising from reporting biases).	p.9-10
Certainty assessment	15	Describe any methods used to assess certainty (or confidence) in the body of evidence for an outcome.	NA
**RESULTS**			
Study selection	16a	Describe the results of the search and selection process, from the number of records identified in the search to the number of studies included in the review, ideally using a flow diagram.	p.11
	16b	Cite studies that might appear to meet the inclusion criteria, but which were excluded, and explain why they were excluded.	p.11
Study characteristics	17	Cite each included study and present its characteristics.	p.11-13
Risk of bias in studies	18	Present assessments of risk of bias for each included study.	p.15-16; Table S8
Results of individual studies	19	For all outcomes, present, for each study: (a) summary statistics for each group (where appropriate) and (b) an effect estimate and its precision (e.g. confidence/credible interval), ideally using structured tables or plots.	p.12-14; Table S5
Results of syntheses	20a	For each synthesis, briefly summarise the characteristics and risk of bias among contributing studies.	Table S6
	20b	Present results of all statistical syntheses conducted. If meta-analysis was done, present for each the summary estimate and its precision (e.g. confidence/credible interval) and measures of statistical heterogeneity. If comparing groups, describe the direction of the effect.	NA
	20c	Present results of all investigations of possible causes of heterogeneity among study results.	NA
	20d	Present results of all sensitivity analyses conducted to assess the robustness of the synthesized results.	NA
Reporting biases	21	Present assessments of risk of bias due to missing results (arising from reporting biases) for each synthesis assessed.	NA
Certainty of evidence	22	Present assessments of certainty (or confidence) in the body of evidence for each outcome assessed.	NA
**DISCUSSION**			
Discussion	23a	Provide a general interpretation of the results in the context of other evidence.	p.18
	23b	Discuss any limitations of the evidence included in the review.	p.22-23
	23c	Discuss any limitations of the review processes used.	p.22-23
	23d	Discuss implications of the results for practice, policy, and future research.	p.18-22

Section and Topic	Item #	Checklist item	Location where item is reported
**OTHER INFORMATION**			
Registration and protocol	24a	Provide registration information for the review, including register name and registration number, or state that the review was not registered.	p.2
	24b	Indicate where the review protocol can be accessed, or state that a protocol was not prepared.	p.2
	24c	Describe and explain any amendments to information provided at registration or in the protocol.	NA
Support	25	Describe sources of financial or non-financial support for the review, and the role of the funders or sponsors in the review.	p.25
Competing interests	26	Declare any competing interests of review authors.	p.25
Availability of data, code and other materials	27	Report which of the following are publicly available and where they can be found: template data collection forms; data extracted from included studies; data used for all analyses; analytic code; any other materials used in the review.	NA

Table S3. Search terms used in EbscoHost, IEEE Explore, Scopus, PubMed, and Web of Science databases.

**EbscoHost**	valid* OR reliab* OR accuracy OR precision OR specificity OR sensitivity OR reproduc* OR OR Feasibility OR criterion OR comparability OR “construct validity” OR calibration OR determination OR recognition<br>*AND*<br>acceleromet* OR “wearable device*” OR “wearable technology” OR “wearable tracker” OR “motion sensor” OR pedomet* OR inclinom* OR “actigraph*” OR “activity monitor*” OR “activity tracker*” OR “motion sensor” OR “motion sensing” OR “heart rate monitor*” OR “fitness tracker*”<br>*AND*<br>“physical activity” OR “physical fitness” OR walking OR exercis* OR “energy expenditure” “step-count” OR “locomotor activity” OR “ambulatory movement” OR “activity intensity” OR OR “human movement classification” OR “free-living condition” OR locomotion OR “activity patterns” OR sport OR “everyday activities” OR “free-living activites” OR Sedentar* OR sitting OR “sedentary behav*” OR “body postures” OR sleep OR “physical behav*”
**IEEE Explore**	valid* OR reliab* OR accuracy OR precision OR specificity OR sensitivity OR reproduc*<br>*AND*<br>acceleromet* OR “wearable” OR pedomet* OR inclinom* OR “activity monitor*” OR “heart rate monitor*” OR “fitness tracker*”<br>*AND*<br>“physical activity” OR exercis* OR “energy expenditure” “step-count” OR “free-living activites” OR sedentar* OR sleep
**Scopus**	(TITLE-ABS-KEY(valid* OR reliab* OR accuracy OR precision OR specificity OR sensitivity OR reproduc* OR feasibility OR criterion OR comparability OR calibration OR determination OR recognition) *AND* TITLE-ABS-KEY(acceleromet* OR “wearable device*” OR “wearable technology” OR “wearable tracker” OR “motion sensor” OR pedomet* OR inclinom* OR “actigraph*” OR “activity monitor*” OR “activity tracker*” OR “motion sensor” OR “motion sensing” OR “heart rate monitor*” OR “fitness tracker*”) *AND* TITLE-ABS-KEY(“physical activity” OR “physical fitness” OR walking OR exercis* OR “energy expenditure” “step-count” OR “locomotor activity” OR “ambulatory movement” OR “activity intensity” OR “free-living condition” OR locomotion OR “activity patterns” OR sport OR “everyday activities” OR “free-living activites” OR sedentar* OR sitting OR “sedentary behav*” OR “body postures” OR sleep OR “physical behav*”)
**PubMed**	((valid*[Title/Abstract] OR reliab*[Title/Abstract] OR accuracy[Title/Abstract] OR precision[Title/Abstract] OR specificity[Title/Abstract] OR sensitivity[Title/Abstract] OR reproduc*[Title/Abstract] OR feasibility[Title/Abstract] OR criterion[Title/Abstract] OR comparability[Title/Abstract] OR calibration[Title/Abstract] OR determination[Title/Abstract] OR recognition[Title/Abstract]) *AND* (acceleromet*[Title/Abstract] OR “wearable device*”[Title/Abstract] OR “wearable technology”[Title/Abstract] OR “wearable tracker”[Title/Abstract] OR “motion sensor”[Title/Abstract] OR pedomet*[Title/Abstract] OR inclinom*[Title/Abstract] OR “actigraph*”[Title/Abstract] OR “activity monitor*”[Title/Abstract] OR “activity tracker*”[Title/Abstract] OR “motion sensor”[Title/Abstract] OR “motion sensing”[Title/Abstract] OR “heart rate monitor*”[Title/Abstract] OR “fitness tracker*”[Title/Abstract])) *AND* (“physical activity”[Title/Abstract] OR “physical fitness”[Title/Abstract] OR walking[Title/Abstract] OR exercis*[Title/Abstract] OR “energy expenditure” “step-count”[Title/Abstract] OR “locomotor activity”[Title/Abstract] OR “ambulatory movement”[Title/Abstract] OR “activity intensity”[Title/Abstract] OR “free-living condition”[Title/Abstract] OR locomotion[Title/Abstract] OR “activity patterns”[Title/Abstract] OR sport[Title/Abstract] OR “everyday activities”[Title/Abstract] OR “free-living activites”[Title/Abstract] OR sedentar*[Title/Abstract] OR sitting[Title/Abstract] OR “sedentary behav*”[Title/Abstract] OR “body postures”[Title/Abstract] OR sleep[Title/Abstract] OR “physical behav*”[Title/Abstract])
**Web of Science**	(((((TS=(Valid* OR reliab* OR accuracy OR precision OR specificity OR sensitivity OR reproduc* OR feasibility OR criterion OR comparability OR calibration OR determination OR recognition) AND TS=(acceleromet* OR “wearable device*” OR “wearable technology” OR “wearable tracker” OR “motion sensor” OR pedomet* OR inclinom* OR “actigraph*” OR “activity monitor*” OR “activity tracker*” OR “motion sensor” OR “motion sensing” OR “heart rate monitor*” OR “fitness tracker*”) AND TS=(“physical activity” OR “physical fitness” OR walking OR exercis* OR “energy expenditure” “step-count” OR “locomotor activity” OR “ambulatory movement” OR “activity intensity” OR “free-living condition” OR locomotion OR “activity patterns” OR sport OR “everyday activities” OR “free-living activites” OR sedentar* OR sitting OR “sedentary behav*” OR “body postures” OR sleep OR “physical behav*”) NOT KP=valid*) NOT KP=reliab*)NOT KP=recog*) NOT KP=feasib*) NOT KP=sensitiv*)

Figure S1. Risk of bias: Classification tree for the judgment of overall study quality.

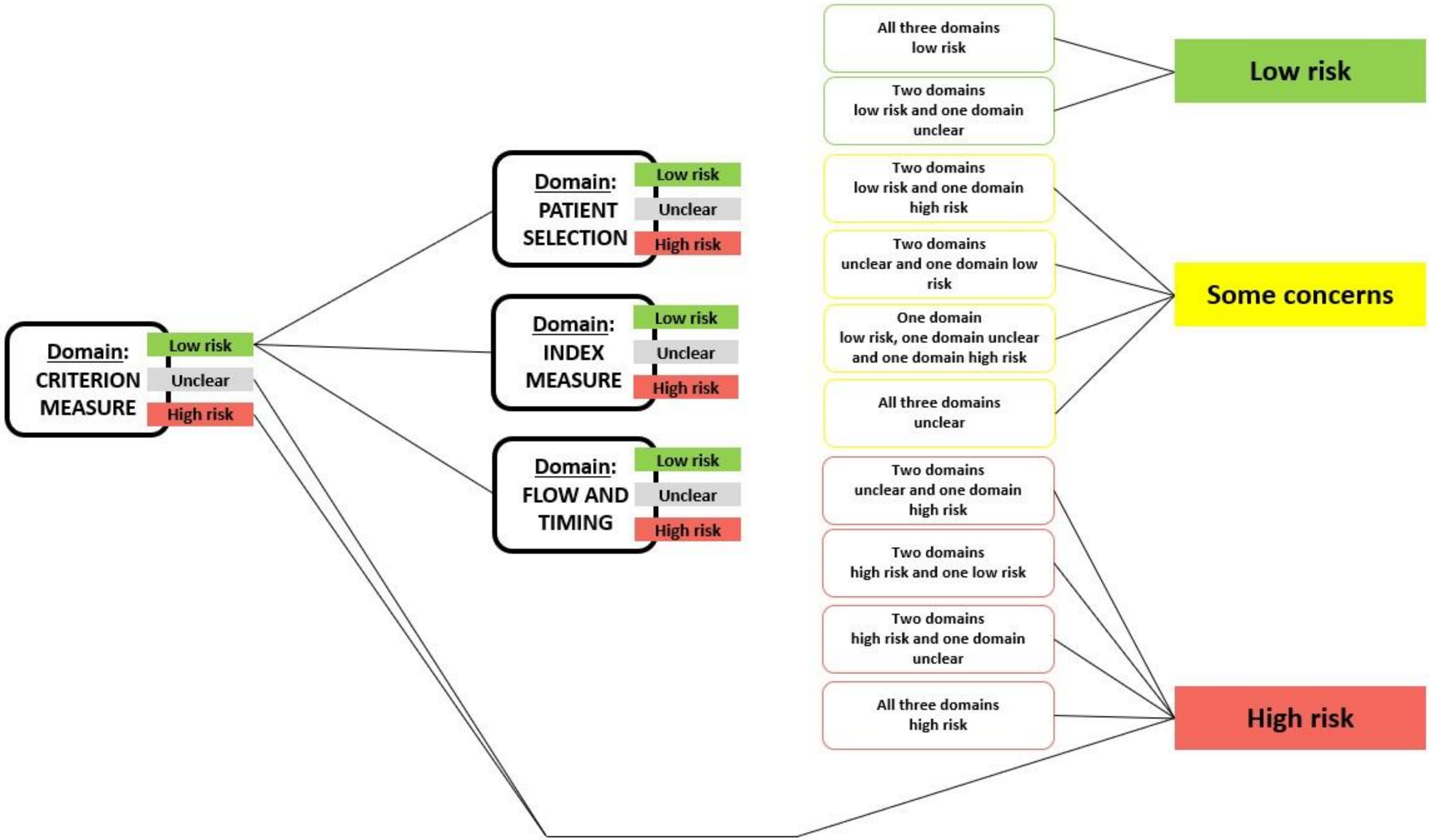

Table S4. Data extraction.

No.	Author (year, location)	Study population (N, age ± SD or range [yrs], % female, ethnicity)	Duration of measurement (validation time)	Wearable (placement; software; epoch-length; algorithm/cut-point)	Dimension (Outcome(s))	Criterion measures	Statistical analysis	Study conclusion	Overall risk of bias (low, some, high)	Funding/ Conflict of interest
1	Agogo et al. (2018, Netherlands)	Adults (N=69; 57.1±9.2 yrs; 46.4% females; NR)	7 days	*ActiGraph GT3X* (hip; ActiLife v6.6; NR; Freedson VM3)	Intensity (Total energy expenditure)	Doubly labelled water	Kernel density plots; Bland-Altman analysis; Shapiro-Wilk test	The GT3X accelerometer would underestimate mean TEE; lead to minimal loss in statistical power to detect significant association; and would result in biased estimate of the association between TEE and a health outcome.	Some	N[1]/N
2	Albright & Jerome (2011, United States)	Adults (N=30; 45.3±22.3 yrs; NR; NR)	< 1 day (approx. 8 hours)	*Oregon Scientific PE829*; *Sportline 343*; *Brookstone*; *Talking Pedometer*; *Accusplit Alliance AL300* (all waist; NR; NR; NR)	Intensity (Steps)	Wearable (Digi-Walker SW-200 (waist))	ANOVA; Percentage error; Bland-Altman analysis	This study found that the Accusplit Alliance AL300 was the most accurate talking pedometer for measuring the daily accumulation of steps; and that it is appropriate for use in both research on and the promotion of physical activity.	High	N/NR
3	Alharbi et al. (2016, Australia)	Patients with coronary heart disease (N=48; 65.6±6.9 yrs; 47.9% females; NR)	4 days	*Fitbit-Flex* (wrist; smartphone or computer application; 1 minute; NR)	Intensity (Steps; Time spent in MVPA)	Wearable (ActiGraph GT3X (waist))	Pearson correlation; Bland-Altman analysis; Paired t-test; Linear regression	Fitbit-Flex is accurate in assessing attainment of physical activity guideline recommendations and is useful for monitoring physical activity in cardiac patients. The device does; however; slightly over-estimate step counts and MVPA.	High	N/N
4	Ameen et al. (2019, Austria)	Adults (N=19; 29±13 yrs; 68.4% females; NR)	8 days (8 nights)	*Mi Band* (wrist; Mi Fit software; 30 sec.; algorithm v1.1.14); *MotionWatch 8* (wrist; MotionWare software v1.1.20; 30 sec; NR)	Biological state (Total sleep time)	Polysomnography	Spearman correlation; Bland-Altman analysis; Sensitivity; Positive predictive value; Cohen´s kappa	The available sleep trackers do not provide meaningful sleep analysis but may be interesting for simply tracking time in bed.	Some	N/N
5	An et al. (2017, United States)	Adults (N=35; 31.0±11.8 yrs; 51.2% females; NR)	1 day	*SenseWear Armband Mini* (upper arm; NR; NR; NR); *Basis B1 Band* (wrist; NR; NR; NR); *Withings Pulse* (waist; NR; NR; NR); *Misfit Shine* (wrist; NR; NR; NR); *Fitbit Flex* (wrist; NR; NR; NR); *Fitbit Zip* (hip; NR; NR; NR); *Garmin Vivofit* (wrist;	Intensity (Steps)	Wearable (New Lifestyle (waist))	Mean absolute percentage error; Pearson correaltion; Bland-Altman analysis; ANOVA	The results show that the Fitbit Zip and Withings Pulse provided the most accurate measures of step count under all three different conditions (i.e. treadmill; over-ground; and 24-hour condition); and considerable variability in accuracy across monitors and also by speeds and conditions.	High	NR[3]/N

				NR; NR; NR); *Jawbone UP24* (wrist; NR; NR; NR); *Nike + FuelBand SE* (wrist; NR; NR; NR); *Polar Loop* (wrist; NR; NR; NR)						
6	Ancoli-Israel et al (1997, United States)	Nursing-home patients (N=10; 86.4±6.0 yrs; 82% females; NR)	1 day (1 night)	*Actillume recorder* (wrist; NR; 1 minute; NR)	Biological state (Total sleep time)	Polysomnography	Spearman correlation; Sensitivity; Specificity	The Actillume is the most feasible technique for studying sleep and wake activity in demented nursing-home patients.	High	N/NR
7	Annegarn et al. (2011, Netherlands)	Patients with chronic heart failure or chronic obstructive pulmonary disease (N=10; 23.8±3.6 yrs, 54.2±16.8 yrs; NR; NR)	< 1 day (approx. 1 hour)	*CAM* (leg, trunk; NR; NR; NR)	Posture/Activity Type (walking, standing, sitting)	Observation (video)	Generalized linear mixed models; Bland-Altman analysis; Cohen´s kappa	The CAM is a promising single-sensor unobtrusive tool for providing accurate data on the type and duration of daily activities in the home environment of patients with chronic organ failure.	High	N/NR
8	Assah et al. (2011, Cameroon)	Adults (N=35; 34.2±7.3 yrs; 51.5% females; NR)	7 days	*Actiheart* (chest; Actiheart commercial software; 1 minute; branched equation model)	Intensity (Energy expenditure)	Doubly labelled water	T-test; Pearson correlation; Bland-Altman analysis	Combined HR and movement sensing is a valid method for estimating free-living PAEE on group level in adults in sub-Saharan Africa.	Some	N/N
9	Au-Yeung et al (2020, United States)	Adults (N=11; 72.4±5.5 yrs; 70% females; NR)	max. 14 days	*Withings Activite* (non-dominant wrist; NR; NR; proprietary algorithm); *ActiGraph wGT3x* (waist; non-dominant wrist; ActiLife 6; NR; NR)	Intensity (Steps)	Werable (PiezoRxD Pedometer (waist))	Pearson correlation; Linear regression; Bland-Altman analysis	Although the Withings Activite underestimated steps, they may be used in studies to estimate relative level of physical activity in free-living conditions since they have good correaltion with other well-validated devices.	High	N/NR
10	Baandrup & Jennum (2015, Denmark)	Patients with schizophrenia or bipolar disorder (N=42; 46.1±9.5 yrs; 40.5% females; NR)	1 day (1 night)	*Actiwatch Spectrum* (wrist; Actiware software v6.0.0, 30 sec.; NR)	Biological state (Total sleep time)	Polysomnography	Paired t-test; Bland-Altman analysis; Intraclass correlation coefficient	Actigraphy reliably measures the total sleep time in this specific patient population. For patients without extensive periods of wakefulness after sleep onset; actigraphy might provide a useful measure of sleep efficiency; sleep latency; and number of awakenings.	High	N/N
11	Bai et al. (2021, United States)	Adults (N=48; 26.8±3.0 yrs; 65% females; NR)	1 day	*Apple Watch series 2* (left wrist; NR; NR; NR); *Fitbit Alta* (right wrist; NR; NR; NR); *Fitbit Charge 2* (right wrist; NR; NR; NR)	Intensity (Steps; Time spent in MVPA)	Wearable (ActiGraph GT3X+ (hip); Yamax Digi-Walker SW-200 (waist))	Pearson correlation; Mean absolute percent error; Equivalence testing; Bland-Altman analysis	All three consumer monitors estimated step counts fairly accurately; and both the Charge2 and Apple2 reported reasonable heart rate estimation. However; all monitors substantially	High	N/N

								underestimated MVPA in free-living settings.		
12	Barkley et al. (2019, United States)	College students (N=15; 21.5± 0.8 yrs; 66.7% females; NR)	3 days	*Movband 2* (non-dominant wrist; NR; NR; proprietary algorithm)	Intensity (Counts (moves))	Wearable (ActiGraph GT1M (waist))	Pearsons correlation; Mixed model regression	The low-cost; Movband acceleromete appears to provide a valid assessment of physical activity behaviour/intensity.	High	NR/N
13	Barone-Gibbs et al. (2020, United States)	Pregnant women (N=58; 31.5±4.8 yrs; 100% females; White (N=44); Black (N=7); Asian (N=3); Multiracial (N=4))	7 days	*ActiGraph GT3X* (torso; ActiLife Software v6.13.3; 1 minute; NR)	Intensity (Sedentary time)	Wearable (ActivPAL3 micro (thigh))	Bland-Altman analysis; Pearson correlation; Intraclass correlation coefficient	Compared to activPAL; waist-worn GT3X produced moderate agreement; though similar mean estimates of SED across pregnancy.	High	N/N
14	Barreira et al. (2015, United States)	Adults (N=15; 27.5±2.5 yrs; 67% females; NR)	7 days	*ActiGraph GT3X+* (waist; ActiLife software v6.0 or higher; 1 minute; NR)	Posture/Activity Type (Breaks in sedentary time (sit-to-stand transitions))	Wearable (ActivPAL (right thigh))	Dependent t-test	The AG detected a significantly higher number of breaks in sedentary time; the majority of which do not correspond to sit-to-stand transitions as measured by the AP.	High	N/N
15	Bartholdy et al. (2018, Denmark)	Patients with knee osteoarthritis (N=18; 67.3±6.5 yrs; 75% females; NR)	1 day	*SENS Motion System* (thigh; SENS motion plus v1.3.0; NR; algorithm reported)	Postures/Activity Type (sedentary, walking, standing, other activities)	Self-reported activity diary	Percentage of agreement	The SENS motion activity measurement system can be regarded as a reliable and valid device for measuring sedentary behaviour in patients with knee OA; whereas detection of walking is not reliable and would require further work.	High	NR/Y
16	Beattie et al. (2017, United States)	Adults (N=60; 34±10 yrs; 40% females; NR)	1 day (1 night)	*Fitbit Surge* (each wrist; NR; NR; peak detector algorithm)	Biological state (Total sleep time)	Polysomnography	Kappa statistics; Sensitivity; Specificity; Accuracy; Paired t-test; Bland-Altman analysis	The results indicate that a reasonable degree of sleep staging accuracy can be achieved using a wrist-worn device; which may be of utility in longitudinal studies of sleep habits.	High	Y[2]/NR
17	Berendsen et al. (2014, Netherlands)	Adults (N=9; 27.2±8.3; 56% females; NR)	Min. 3 days	*ActivPAL3* (thigh; ActivPAL software 6.0.2; 1 sec.; NR); *ActiGraphGT3X* (waist; Actilife 5.10.0; 1 sec.; NR); *CAM* (thigh; Custom Matlab program; 1 sec.; NR)	Posture/Activity Type (sitting, lying, standing, walking)	Self-reported activity diary	Intraclass correlation coefficient; Bland Altman analysis; Friedman's ANOVA; Wilcoxon signed rank test	The ActivPAL3 is valid; reproducible and user friendly. The posture classification by the ActiGraphGT3X is not valid; but reflection of walking intensity and user friendliness are good. The CAM is valid; however; reproducibility at higher walking intensity and user friendliness might cause problems.	High	N/N
18	Berninger et al. (2018, Netherlands)	Adults (N=7; 34±10; 85.7% females; NR)	Min. 1 day	*VitaBit* (trouser pocket; VitaBit firmware; 30 sec.; proprietary algorithm)	Posture/Activity Type (sitting; standing; walking)	Wearable (ActiGraph GT3X+ (thigh))	Sensitivity; Specificity; Negative predictive rates; Positive predictive rates	As the results are not transferrable to daily life activities, a direct observation study in a free-living setting is recommended.	High	Y/Y

19	Berryhill et al. (2020, United States)	Adults (N=32; 23.8±5 yrs; 65.6% females; NR)	1 day (1 night)	*WHOOP Strap 2.0* (wrist; WHOOP cloud; NR; NR)	Biological state (Sleep duration)	Polysomnography	Interclass correlation coefficient	In healthy people; wearables can improve sleep quality and accurately measure sleep and cardiorespiratory variables.	High	Y/N
20	Blackwell et al. (2008, United States)	Community-dwelling women (N=68; 81.9±3.8 yrs; 100% females; African Americans 16.2%)	3 days	*SleepWatch-O* (wrist; NR; 1 minute; Cole-Kripke algorithm)	Biological state (Total sleep time)	Polysomnography	Paired t-tests; Intraclass correlation coefficient; Two-way ANOVA; Bland-Altman analysis	Sleep parameters from actigraphy corresponded reasonably well to PSG in this population; with the PIM mode of actigraphy correlating highest.	Some	N/N
21	Blackwell et al. (2011, United States)	Community-dwelling men (N=889; 76.28±5.47 yrs; 0% females; Caucasian 93.14%, African American 2.25%, Asian 2.14%, Hispanic/Other 2.47%)	4 days	*SleepWatch-O* (wrist; NR; 1 minute; Cole-Kripke algorithm)	Biological state (Total sleep time)	Polysomnography	Wilcoxon rank-sum tests; Paired t-tests; Correlation; Intraclass correlation coefficient; Bland-Altman analysis; Linear regression	Sleep parameters from the proportional integration mode (PIM) and time above threshold modes of actigraphy corresponded reasonably well to PSG in this population; with the PIM mode correlating highest.	Some	N/Y
22	Block et al. (2019, United States)	People with multiple sclerosis (N=36; 53.4±11.7 yrs; 58.1% females; NR)	7 days	*Fitbit Flex* (wrist; NR; NR; NR); *Fitbit Flex 2* (wrist; NR; 1 minute; NR)	Intensity (Steps)	Wearable (ActiGraph GT3X (wrist))	Bland-Altman analysis; Intraclass correlation coefficient; Paired t-test	Steps from Flex and Flex2 can be used interchangeably. Differences in total step count between ActiGraph and Flex devices can make cross-device comparisons of numerical step-counts challenging particularly for faster walkers.	High	N/N
23	Blondeel et al. (2020, Belgium)	Patients with chronic obstructive pulmonary disease (N=28; 66±8 yrs; 39% females; NR); Healty control (N=14; 69±7 yrs; 36% females; NR)	14 days	*Fitbit Zip* (hip; online Fitbit platform; NR; motion pattern algorithms); *Fitbit Alta* (wrist; online Fitbit platform; NR; motion pattern algorithms)	Intensity (Steps)	Wearable (Dynaport Movemonitor (lower back))	Univariate linear analysis; Unpaired t-test; Bland-Altman analysis; Kendall correlation	Although the accuracy of hip worn consumer-based activity trackers in patients with COPD and wrist worn activity trackers in healthy subjects as clinical endpoints is unsatisfactory; these devices are valid to use as a coaching tool.	High	N/N
24	Boeselt et al. (2016, Germany)	Patients with chronic obstructive pulmonary disease (N=20; 66.4± 7.4 yrs; 15% females; NR)	3 days	*Polar A300*™ (wrist; NR; NR; NR)	Intensity (Steps; Energy expenditure (calories, MET); Time spent in PA)	Wearable (Sense Wear (arm))	Bland-Altman analysis; Intraclass correlation coefficient; Linear regression; Mann-Whitney U test	The A300 ™ device with easy practical usability was shown not to be inferior for assessment of physical activity time; step count and calorie consumption in COPD patients when compared with the SWA.	High	N/N
25	Bonomi et al. (2009, Netherlands)	Adults (N=15; 41±11 yrs; 40% females; NR)	5 days	*Tracmor$_D$* (lower back; NR; NR; NR)	Intensity (Energy expenditure)	Doubly labelled water	Bland-Altman analysis; Multiple linear regression; Pearson correlation	Identification of activity types combined with MET intensity values improves the assessment of energy expenditure compared with activity counts.	Some	Y/NR
26	Bonomi et al. (2010, Netherlands)	Adults (N=30; 41±11 yrs; 40% females; NR)	14 days	*Tracmor$_D$* (lower back; Philips New Wellness Solutions; NR; equation reported)	Intensity (Total energy expenditure)	Doubly labelled water	Multiple linear regression; Pearson correlation	The Tracmor$_D$ is a highly accurate instrument for predicting free-living energy expenditure.	High	Y/NR

27	Bourke et al (2016, Norway)	Adults (N=20; 76.4±5.6 yrs; 50% females; NR)	1 day	*ActiGraph GT3X+* (right hip; ActiLife v6.11.8; NR; state machine algorithm)	Posture/Activity Type (standing, walking, lying, stair climbing)	Observation (video)	Cohen's kappa; Corrected kappa; Krippendorff's alpha	This is one of the most detailed validations of a body worn sensor algorithm to date and offers an insight into the challenges of developing a real-time physical activity classification algorithm for a tri-axial accelerometer-based sensor worn at the waist.	Some	N/NR
28	Bourke et al. (2019, Norway)	Adults (N=20; 76.4±5.6 yrs; 75% females; NR)	NR	*ActivPAL3* (left thigh; activPAL 122 software; v7.1.2.142; NR; proprietary algorithm)	Posture/Activity Type (sitting/lying, standing, walking); Intensity (Steps)	Observation (video)	Percentage of agreement; F1-score; Sensitivity; Specificity; Positive/negative predictive value	This validation study provides a detailed insight into the physical activities that the activPAL3 classifies in its 3 main activity categories; step detection and postural transition analysis in a laboratory and a free-living setting.	High	N/NR
29	Brage et al. (2015, United Kingdom)	Adults (N=46; males: 33.2± 8.1 yrs; females: 35± 10.1 yrs; 50% females; NR)	14 days	*Actiheart* (chest; NR; 1 minute; branched equation)	Intensity (Total energy expenditure)	Doubly labelled water	Bland-Altman analysis; Paired t-test; Root mean square error; Pearson correlation	Both accelerometry and heart rate may be used to estimate EE in adult European men and women; with improved precision if combined and if heart rate is individually calibrated.	Some	N/N
30	Brazeau et al. (2016, Canada)	Adults (N=20; 26.2±3.6 yrs; NR; 85% Caucasian)	7 days	*SenseWear Armband Pro 3* (upper arm; Innerview Research Software v6.1; NR; NR); *Actical* (hip; manufacturer software v2.1; 1 minute; 2-regression equation)	Intensity (Energy expenditure)	Doubly labelled water	Bland–Altman analysis; Pearson Correlation; Intraclass correlation coefficient; Paired t-test	Acceptable estimation of total energy expenditure was observed with the SWA. Both devices were reliable but not accurate for energy expenditure’s estimations during rest and for specific exercises.	Some	N/N
31	Breteler et al. (2019, Netherlands)	Adults (N=30; 40.4±10.6; 46% females; NR)	3 days	*Apple Watch* (wrist; WatchOS; NR; NR); *Misfit Shine* (right hip; Misfit App; NR; NR); *iHealth Edge* (right hip, wrist; iHealth MyVitals; NR; NR); *Yamax Digiwalker SW-200* (right hip, wrist; NR; NR; NR)	Intensity (Steps)	Wearable (ActiGraph GT9X (right hip; wrist))	Bland-Altman analysis; Median absolute difference	Validity varied widely between devices; with the Apple Watch being the most accurate and Yamax Digiwalker the least accurate for step count in free-living conditions.	High	NR/N
32	Brewer et al. (2017, United States)	Adults (N=53; 28.1±9 yrs; 83% females; NR)	7 days	*Fitbit Charge HR; Fitbit Charge; Fitbit Flex; Fitbit Surge; Fitbit Zip; Fitbit Alta* (all non-dominant wrist; NR; NR; proprietary algorithms)	Intensity (Steps; Time spent physically active)	Wearable (ActiGraph GT3X+ (right hip))	Pearson correlation; Paired t-tests; Bland-Altman analysis	The data produced by the Fitbit were consistent with the ActiGraph when the means of each device were compared over the 1-day and 7-day time periods.	High	N/N
33	Brooke et al. (2017, United States)	Adults (N=95; 28.5±9.9 yrs; 64.2% females; NR)	2 days (1 night)	*Nike+ FuelBand SE; Garmin VivoFit; Misfit Shine; Fitbit Flex; Jawbone UP; Polar*	Intensity (Energy expenditure);	Wearable (SenseWear Armband Mini	Pearson correlation; Mean absolute percentage error; Bland-Altman analysis	This study suggests that the PL and FC provide a reasonable estimate of EE under free-living conditions. The PL; FC; and MF were the most	High	NR/N

				Loop; Fitbit Charge HR; SenseWear Armband Mini (all wrist; NR; NR; NR)	Biological state (Sleep time)	(arm)); Sleep log		valid monitors used for measuring Sleep time.		
34	Bruignaux et al. (2010, France)	Adults (N=31; age range 35-55 yrs; 51.6% females; NR)	< 1 day (approx. 2hours)	*Polar Activity Watch 200* (wrist; NR; NR; NR)	Intensity (Energy expenditure; Steps; Time spent in PA)	Indirect calorimetry	Linear regression; Pearson correlation; Students t-test; Bland-Altman analysis	The AW200 appears to be very useful and accurate device for measuring EE during exercise in recreational hikers and provides a useful tool for keeping track of personal EE.	High	Y/N
35	Busse et al. (2009, United Kingdom)	Adults (N=22; 26.9 yrs; NR; NR)	4 days	*Stepwatch Activity Monitor* (right ankle; NR; NR; NR)	Intensity (Time spent in different intensities (inactive, low, MVPA))	Self-reported activity diary	Spearman correlation; Wilcoxon test	Activity monitors provide information that is related to actual activity and provide accurate and reliable data when tested on functional walking circuits.	High	N/N
36	Bussmann et al. (1998, Netherlands)	Failed back surgery patients (FPS) (N=10; 49.5±9.3 yrs; 75% females; NR)	< 1 day (28-82 min)	*Activity Monitor IC-3031* (each thigh; NR; NR; NR)	Posture/Activity Type (static, dynamic)	Observation (video)	Agreement; Sensitivity; Predictive value; Wilcoxon signed-ranks test	The AM appeared to be a valid instrument to quantify aspects of behaviour of FPS patients; such as duration of activities and number of transitions.	High	NR/NR
37	Cabanas-Sánchez et al. (2018, Spain)	Adults (N=50; 70.36±4.32 yrs; 70% females; NR)	2 days	*IDEEA* (chest, front of both thighs, both feet; NR; NR; algorithm reported)	Biological state (Sleep time)	Self-reported sleep diary and expert visual analysis of accelerometer raw data	Pearson correaltion; Mean differences; Mean percentage errors; Accuracy; Sensitivity; Specificity; Bland-Altman analysis	These four algorithms can be used to identify easily and with adequate accuracy the sleep period time using the IDEEA activity monitor from 24 h free-living data in older adults.	High	N/N
38	Calabro et al. (2015, United States)	Adults (N=29; 68.8±6.3 yrs; 58.6% females; NR)	14 days	*SenseWear Mini armband* (wrist; v7.0; NR; algoritm v2.2)	Intensity (Total energy expenditure)	Doubly labelled water	Equivalence testing; Mean absolute percent errors; Pearson correlation; Bland-Altman analysis	The Mini and 7D-PAR provided reasonably valid estimates of TEE but large errors in estimating AEE. The Mini and 7D-PAR have the potential to accurately estimate TEE for older adults.	Some	N/N
39	Campos et al. (2018, Canada)	Individuals with subacute stroke (N=33; 64.9±14.7 yrs; 30.3% females; NR)	< 1 day (7 hours)	*ActiGraph GT3x+* (waist, ankle; ActiLife software v6.0; 1 minute; proprietary algorithm)	Intensity (Steps)	Wearable (Model X6-2mini (ankle))	Paired t-test; Wilcoxon signed rank test; Shrout Fleiss intraclass correlation; Spearman correlation	The AG worn at the unaffected ankle without LFE produced the most accurate step count in people with stroke.	High	NR/N
40	Carpenter et al. (2021, United States)	Adults (N=11; 20.7±0.5 yrs; 73% females; White (91%))	14 days	*Fitbit Charge 3* (wrist; application programming interface; NR; proprietary algorithm)	Posture/Activity Type (Time spent sitting/lying)	Wearable (ActivPAL4 (thigh))	Paired t-tests; Intraclass correlation coefficient	Author´s suggest that Fitbit could replace activPAL when measuring total sedentary time.	High	N/N
41	Carter et al. (2008, United Kingdom)	Adults (N=14; 19±3 yrs; 0% females; NR)	10 days	*3dNX accelerometer model v2* (right hip; NR; 1 minute; equation reported)	Intensity (Energy expenditure)	Doubly labelled water	Bland-Altman analysis	Although it remains to cross-validate these models in other populations, early indications suggest that the 3dNXTM provides a useful method of predicting	Some	N/NR

								energy expenditure in free-living individuals.		
42	Castner et al. (2019, United States)	Women with asthma (N=47; 40.6±13.0 yrs; 100% females; 44.7% Non-Hispanic Black; 36.2% Non-Hispanic White; 4.3% Hispanic; 10.6% more than one race (Non-Hispanic); 2.1% Hispanic more than one race; 2.1% native Hawaiian)	14 days	*Fitbit Charge* (non-dominant wrist; Fitbit Connect software v2.0.1.6782; 1 minute; proprietary algorithm)	Biological state (Total sleep time)	Wearable (ActiGraph WGT3x+ (non-dominant wrist))	Mann-Whitney U test; Chi-square; Pearson correlation; Equivalence test; Bland-Altman analysis	There were important differences in total sleep time, efficiency, and wake count measures when comparing individual sleep segments versus 24-hour measures of sleep. Fitbit overestimates sleep efficiency and underestimates wake counts in this population compared to actigraphy.	High	N/N
43	Cellini et al. (2016, United States)	Adults (N=30; 19.2 ±0.86 yrs; 50% females; African American (N=4); Asian (N=12); Hispanic (N=11); Caucasian (N=3))	3 days (2 nights)	*ActiGraph GT3X+* (wrist; ActiLife 6.4.3 software; 1 minute; sedentary < 100, LPA 100-1951; MVPA > 1952 cpm); *Actiwatch-64* (non-dominant wrist; Actiware 5.52.0003 software; 1 minute; proprietary algorithm)	Biological state (Total sleep time); Intensity (Time spent in different intensities (sedentary, LPA, MVPA); Activity energy expenditure)	Wearables (Actiwatch-64 (wrist); ActiGraph GT3X+ (hip))	ANOVA; Intraclass correlation coefficient; Mean absolute percent errors; Equivalence test; Bland-Altman analysis; Limits of agreement	Overall our results showed good agreement of the GT3X+ with AW-64 for assessing sleep but a lack of agreement between AW-64 and GT3X+ for physical activity and sedentary behaviours.	High	N/N
44	Chakravarthy & Resnick (2017, United States)	Adults living in retirement community (N=14; 86.28±5.9 yrs; 64% females; NR)	3 days	*MotionWatch 8* (wrist; NR; 1 minute; sedentary < 178, light 179-561, moderate >562, vigorous > 1020 cpm)	Intensity (Counts)	Self-reported activity diary	Correlation; ANOVA	The findings provide preliminary support for the reliability and validity of the MotionWatch 8 when used with older adults.	High	NR/NR
45	Chocquette et al. (2009, Canada)	Adults (N=17; 66.1±5.2 yrs; 41.2% females; NR)	7 days	*Caltrac accelerometer* (waist; NR; NR; NR)	Intensity (Total energy expenditure)	Doubly labelled water	Mann-Whitney U test; Spearman correlation	Results suggest that TEE may be estimated with good accuracy using fat-free mass, the cost of standing still, and the cost of walking at 3 km*h^{-1}.	High	N/N
46	Chowdhury et al. (2017, United Kingdom)	Adults (N=30; 27±6 yrs; 50% females; NR)	1.5 days (36 hours)	*Apple Watch* (wrist; Watch OS v1.0.1; NR; NR); *Microsoft Band* (wrist; firmware v10.2.2818.9; app v1.3.10506.1; NR; NR); *Fitbit Charge HR* (wrist; Fitbit app v2.9.1; NR; NR); *Jawbone UP24* (wrist; UP app v4.4; NR; algorithm v5.2)	Intensity (Energy expenditure)	Wearable (Actiheart (chest))	Agreement; Bland-Altman analysis; Mean signed error; Mean absolute error; Root mean squared error; Pearson correaltion; Repeated measures ANOVA; Equivalence testing	None of the consumer devices were deemed equivalent to the reference method for daily energy expenditure. For all devices; there was a tendency for negative bias with greater daily energy expenditure.	High	N/N
47	Chu et al. (2017, Singapore)	Adult hospital students and employees (N=104; median 31.0	7 days	*Fitbit Flex* (non-dominant wrist; web-base software	Intensity (Steps)	Wearable (ActiGraph	Spearman correlation; Intraclass correlation coefficient; Mean	There were high correaltion and agreement in steps between Fitbit Flex and ActiGraph. However,	High	N/N

		yrs; 66.3% females; NR)		application; 1 minute; NR)		wGT3X-BT (right hip))	absolute percentage error; Bland-Altman analysis	findings suggested discrepancies in steps between devices.		
48	Clemes et al. (2010, United Kingdom)	Adults (N=134; 36.4±18.1 yrs; NR; NR)	< 1 day (approx. 12 hours)	*Silva Pedometer* (waist; NR; NR; NR); *NL-1000* (waist; NR; NR; NR)	Intensity (Steps)	Wearable (ActiGraph GT1M (waist))	Absolute percentage error; Bland-Altman analysis	The findings suggest the Silva pedometer is unacceptably inaccurate for activity promotion purposes, particularly in overweight and obese adults.	High	N/N
49	Colbert et al. (2011, United States)	Adults (N=56; 74.7±6.5 yrs; 79% females; 98% White)	10 days	*ActiGraph GT1M* (right waist; NR; 10 sec; Freedson and Crouter equations); *SenseWear Pro 3 Armband* (wrist; Innerview Research software v5.12; NR; NR)	Intensity (Total energy expenditure; Physical activity energy expenditure)	Doubly labelled water	Bland-altman analysis; Wilcoxon rank sum test	Objective devices more appropriately rank PAEE than self-reported instruments in older adults, but absolute estimates of PAEE are not accurate.	Low	N/N
50	Collins et al. (2019, United States)	Osteoarthritis patients (N=15; 68±8 yrs; 67% females; NR)	14 days	*Fitbit Charge 2* (wrist; in-house Python program incorporating the Fitbit application programming interface; NR; proprietary algorithm); *ActiGraph GT3X+* (wrist; hip; ActiLife v6.13.3; 1 minute; proprietary algorithm)	Intensity (Steps; Time in different intensities (sedentary, MVPA))	Wearable (ActiGraph GT3X (hip))	Intraclass correlation coefficient; Percentage bias	Fitbit overestimates steps and underestimates MVPA in knee OA subjects.	High	N/N
51	Connolly et al. (2020, United States)	Pregnant women (N=39; 28.6±8.4; 100% females; NR)	3 days	*Omron HJ-720* (waist; NR; NR; NR); *New Lifestyles 2000* (pocket; NR; NR; NR); *Fitbit Flex* (wrist; NR; NR; NR); *ActiGraph GT9X Link* (waist; NR; NR; NR)	Intensity (Steps)	Wearable (StepWatch (ankle))	Mean absolute percent error; One-way ANOVA; Independent-sample t-test; Bland-Altman analysis	The OM; NL; and SW monitors are valid measures for overground step-counting during pregnancy walking. However; the OM and NL significantly underestimate steps by second and third trimester pregnant women in free-living conditions.	High	NR/NR
52	Correa et al. (2016, United States)	Adults (N=87; 44.5+12.8 yrs; 82.8% females; 66.67% Caucasian)	7 days	*Actical physical activity monitor model C* (waist, dominant wrist; NR; 1 minute; NR); *Sensewear armband* (upper arm; NR; NR; proprietary algorithm); *IDEEA* (chest, front of both thighs, both feet; NR; NR)	Intensity (Total energy expenditure; Activity energy expenditure)	Doubly labelled water	Bland-Altman analysis; One-way ANOVA	The results suggest that the ability of energy expenditure and posture allocation to predict weight change is limited, and the accuracy of TEE and AEE measurements vary across activity monitoring devices, with multi-sensor monitors demonstrating stronger validity.	Low	N/NR
53	Cuberek et al. (2010, Czech Republic, Poland)	Young girls (N=135; 18.0±0.6 yrs; 100% females; NR)	7 days	*Yamax Digi-Walker SW-701* (waist; NR; NR; NR)	Intensity (Steps)	Wearable (ActiGraph GT1M (waist))	Spearman correlation; Kruskal-Wallis ANOVA; Wilcoxon-test	The convergent validity of the pedometer can be considered intermediate when used to measure the step counts in free-living physical activity; but should be	High	N/NR

								considered with caution when used to classify participants‘ step counts into corresponding physical activity categories because of a likelihood of ‘erroneous‘ classification in comparison with the accelerometer.		
54	Culhane et al (2004, Ireland)	Adults admitted to rehabilitation unit of hospital (N=5; 52-90 yrs; 60% females; NR)	4 days	*ADXL202 acccelerometer* (thigh, trunk; NR; NR; NR)	Posture/Activity Type (standing, sitting, lying, moving)	Observation (direct)	Accuracy	In a population of older adults, the static activities of sitting, standing and lying and dynamic activities can be distinguished using the technique and threshold values outlined here to a degree of accuracy of 92% and higher.	High	Y/NR
55	Davidson et al. (1997, United Kingdom)	Adults (N=19; 38±9 yrs; 0% females; NR)	9 days	*Polar Sports Tester 1000 HR monitor* (chest; NR; 1 minute; NR)	Intensity (Energy expenditure)	Doubly labelled water	Bland-Altman analysis; Linear regression	HR monitoring can provide a better estimate of 24 h EE of groups than the diary-respirometer method; but show that both methods can introduce errors of 20% or more in individuals	Some	N/NR
56	De Cocker et al. (2012, Belgium)	Adults (N=54; 33.9±11.1 yrs; 57.4% females; NR)	1 day	*Omron HJ-203 pedometer* (front pant pocket, necklace; NR; NR; NR)	Intensity (Steps)	Wearable (Yamax Digiwalker SW-200 (waist))	Spearman correlation; Repeated-measure ANCOVA; Intraclass correlation coefficient	The HJ-203 Omron pedometer showed acceptable accuracy for all wearing positions during stairs walking and treadmill walking at higher speeds; but limited accuracy during free-living conditions when worn at non-traditional wearing positions (necklace and pants pocket).	High	NR/NR
57	Degroote et al. (2018, Belgium)	Adults (N=36; 39.43±17.11 yrs; 50% females; NR)	2 days	*Polar M600* (non-dominant wrist; NR; NR; NR)*; Huawei Watch* (non- dominant wrist; NR; 15 minutes; NR)*; Asus Zenwatch3* (non-dominant wrist; NR; 15 minutes; NR)*; Fitbit Charge* (non- dominant wrist; NR; 1 minute; NR)	Intensity (Steps; Time spent in MVPA)	Wearable (ActiGraph GT3X+ (right waist))	Spearman correlation; Intraclass correlation coefficient; Bland-Altman analysis	In sum, all 4 consumer-level devices can be considered accurate step counters in free-living conditions.	High	NR/N
58	Degroote et al. (2020, Belgium)	Adults (N=19; 37.6±13.4 yrs; 68.4% females; NR)	3 days	*Geonaut On Coach; iWown i5 Plus; MyKronoz ZeFit4; Nokia GO; VeryFit 2.0; Xiaomi MiBand 2; Fitbit Charge 2*; (all non-dominant wrist; properietary software; NR; NR)	Intensity (Steps; Time spent in MVPA); Biological state (Total sleep time)	Wearables (Intensity: ActiGraph GT3X+ (right waist); Biological State: BodyMedia SenseWear	Spearman correlation; Intraclass correlation coefficient; Mean absolute percent error; Bland-Altman analysis	Validity ranged between devices; with Xiaomi having the highest validity for measurement of steps and VeryFit performing relatively strong across both sleep and steps domains.	High	NR/N

						(non-dominant upper arm))				
59	DeShaw et al. (2018, United States)	Adults (N=61; 41.8±9.2 yrs; 53% females; 80% White Non-Hispanic)	7 days	*Fitbit Charge* (non-dominant wrist; NR; 1 minute; customized SIP algorithms)	Intensity (Steps; Time spent in MVPA)	Wearables (ActiGraph GT3X+(right hip); ActivPal3 (right thigh))	Mean absolute percentage error; Root-mean-square error; Bland-Altman analysis; Kappa statistics	The FBC overestimated minutes of MVPA and steps when compared to both reference assessments	High	NR/NR
60	Dickinson et al. (2016, United States)	Adults (N=38; 26.05±7.99 yrs; 60.5% females; NR)	4 days (4 nights)	*Fitbit Charge HR* (wrist; NR; 30 sec; NR)	Biological state (Total sleep time)	Wearable (ActiWatch (wrist))	Linear regression; Bland-Altman analysis	We therefore do not recommend the Fitbit device when accurate point estimates are important.	High	N/N
61	Dieu et al. (2016, France)	Sport science students (N=40; Males 22.9±3.1 yrs, females 21.3±2.1 yrs; 40% females; NR)	1 day	*ActiGraph GT3X* (non-dominant wrist, dominant wrist; NR; NR; NR)	Intensity (Counts)	Wearable (ActiGraph GT3X (waist))	Pearson correlation	Findings suggest that the choice to wear the accelerometer on the nondominant or dominant wrist has no impact on results.	High	NR/N
62	Dominick et al. (2016, United States)	Adults (N=19; Males: 20.0±0.8 yrs; females: 21.3±4.4 yrs; 78.9% females; NR)	7 days	*Fitbit Flex* (dominant wrist; Fitabase; 1 minute; proprietary algorithms)	Intensity (Steps; Energy expenditure (MET); Time spent in different intensities (sedentary, LPA, MVPA))	Wearable (ActiGraph GT3X (dominant hip)	Paired t tests; Generalized linear mixed model; Correlation	Fitbit Flex provides accurate measures of steps for daily activity and minutes of reported exercise, regardless of exercise type.	High	N/N
63	Donahoe et al. (2018, Canada)	Adults (N=35; 23.3±10.1 yrs; 60% females; NR)	7 days	*PiezoRx* (hip; NR; NR; NR); *Fitbit Flex* (wrist; Fitabase; 1 minute; NR); *ActivPAL* (thigh; ActivPAL3 software; 15 sec; NR)	Intensity (Time spent sedentary)	Wearable (Actical (hip))	Pearson correlation; ANOVA; Mean absolute percent error; Equivalence testing; Bland-Altman analysis	Sedentary time derived using the PiezoRx pedometer may be statistically equivalent to the Actical accelerometer; but not the ActivPAL inclinometer or Fitbit Flex.	High	Y/NR
64	Dondzila et al. (2012, United States)	Adults (N=102; Two age groups: 1) 32.9±10.8 yrs; 2) 65.4±6.9 yrs; NR; NR)	1 day	*Omron HJ-720ITC* (left waist; NR; NR; NR); *Kenz lifecorder EX pedometer* (right waist; NR; NR; NR)	Intensity (Steps)	Wearable (New Lifestyles NL-1000 pedometer (left waist))	Mean error score; 95% limits of agreement; One sample t-test	The OM significantly underestimated steps during the 24-hour compared with a standard of care evaluation.	High	N/N
65	Dondzila et al. (2018, United States)	Adults (N=40; 21.6±2.0 yrs; 57.5% females; NR)	1 day	*Fitbit Charge HR* (non-dominant wrist; NR; NR; proprietary algorithms); *Mio Fuse* (non-dominant wrist; NR; NR; NR)	Intensity (Steps)	Wearable (New Lifestyles NL-1000 pedometer (dominant hip))	One-way ANOVA; Bland-altman analysis	Increasing exercise intensity is indicative of heightened accuracy for step detection and kcal estimation for the FB and MF, while decreasing heart rate accuracy for the FB. However, the MF performed poorly for estimating total daily activity.	High	N/NR
66	Durkalec-Michalski et	Adults (N=20; males 26.5±5.0 yrs; females	3 days	*ActiGraph GT1M* (waist; NR; NR; Work-Energy	Intensity (Energy expenditure)	Wearable (S-610 Polar Sport Tester (chest))	Correaltion; One-way ANOVA	Although AM and MR provided less accurate results than HRM in laboratory conditions, there were no	High	NR/NR

	al. (2013, Poland)	26.0±4.5 yrs; 55% females; NR)		Theorem; Freedson's equation)				significant differences between the three methods (HRM, AM and MR) when total daily energy expenditure was calculated for the participants in free-living condition.		
67	Edwardson et al. (2018, United Kingdom)	Adults (N=30; 32.6±8.7 yrs; 56.6% females; NR)	2 days	*Fitbit Flex; Garmin Vivofit3; Jawbone UP24; Polar Loop; Mi Band; Aquarius* (all wrist; NR; NR; NR).	Intensity (Steps)	Wearable (New-Lifestyle NL-80 (waist))	Mean absolute percentage errors; Repeated-measure ANOVA; Bland-Altman analysis	Tracker agreement with the waist-worn pedometer varied widely but trackers on the ND wrist had better agreement. The Mi Band was the most comparable to the pedometer.	High	N/NR
68	Ekelund et al. (2000, Sweden)	Athletes (N=8; 18.5±1.2 yrs; 0% females; NR)	8 days	*CSA activity monitor* (waist; NR; 15 sec; equation reported)	Intensity (Total energy expenditure)	Doubly labelled water	Spearman correlation; two-way MANOVA; Paired t-test; Independent t-test	The relationship between activity counts and total daily energy expenditure seems to be affected by different training conditions.	High	N/NR
69	Farina & Lowry (2017, United Kingdom)	Community-dwelling older adults (N=25; 72.5±4.9 yrs; 48% females; White British (96%); White Irish (4%))	7 days	*Fitbit Charge HR* (dominant wrist; NR; 1 minute; NR); *Misfit Shine* (dominant wrist, dominant waist; NR; NR; NR)	Intensity (Steps)	Wearable (ActiGraph GT3X+; NL-2000i (waist))	Spearman correlation; Pearson correlation; Intraclass correlation coefficient; Bland-Altman analysis	There was also substantial to near perfect agreement between all consumer-level activity monitors and reference devices.	High	NR/N
70	Feito et al. (2015, United States)	Adults (N=24; 23.8±8.8 yrs; NR; NR)	2 days	*ActiGraph GT1M* (hip; ActiLife v5.1; 15 sec; NR); *ActiGraph GT3X* (hip; ActiLife v5.1; 15 sec; NR)	Intensity (Steps)	Wearable (Stepwatch 3 (right ankle))	One-way repeated-measures ANOVA; Pearson correlation; Bland-Altman analysis	Turning the LFE on lessens the underestimation of steps recorded at walking speeds ≤54 $m*min^{-1}$ for both the GT3X and GT1M.	High	N/Y
71	Feito et al. (2012, United States)	Different weigth categories: Normal weight (N=21; 28.3±10.5 yrs, overweight (N=19; 31.2±9.9 yrs, obese (N=16; 29.0± 7.9yrs); 50% females; NR	2 days	*Actical* (left hip; Actireader v2.10; 15 sec, NR); *ActiGraph 7164* (right hip; Actisoft Analysis Software v3.2.1.1; 15 sec; NR); *ActiGraph GT1M* (left hip; Actilife v4.4.1; 15 sec; NR); *ActiGraph GT3X* (right hip; Actilife v4.4.1; 15 sec; NR); *ActivPAL* (right thigh; activPAL Professional Research edition v5.8.5.0; 15 sec; NR); *Digiwalker SW-200* (hip; NR; 15 sec; NR)	Intensity (Steps)	Wearable (Stepwatch 3 (right ankle)	Two-way repeated-measures ANOVA; One-sample t-test; Bland-Altman analysis	We demonstrated that BMI does not affect the step output of commonly used activity monitors during walking.	High	N/N
72	Ferguson et al. (2015, Australia)	Adults (N=21; 32.8±10.2 yrs; 52% females; NR)	2 days	*Nike Fuelband* (left wrist; Nike+ Fuelband iOS app V2.0.0; NR; NR); *Jawbone UP* (left wrist; Jawbone iOS app v2.8.1; NR; NR); *Mistfit*	Biological state (Sleep time); Intensity (Steps, Total energy expenditure,	Wearables (ActiGraph GT3X+ (waist); SenseWear (upper arm))	Bland-Altman analysis; Pearson correlation; Median absolute difference	In free-living conditions, the consumer-level activity monitors showed strong validity for the measurement of steps and sleep duration, and moderate valid for measurement of TDEE and MVPA.	High	NR/N

				Shine (left wrist; Shine iOS app v1.4.0; NR; NR); *Fitbit One; Fitbit Zip* (right waist; Fitbit iOS app v2.0.2., online dashboard software; NR; NR); *Withings Pulse* (right waist; Withing Health Mate iOS app v1.20, online dashboard software; NR; NR); *Striiv Smart pedometer* (right waist; inbuilt device software; NR; NR)	Time spent in MVPA)					
73	Fokkerood et al. (2014, Netherlands)	Adults with Intermitted claudification (N=21; 67±10 yrs; 38% females; NR)	< 1 day (approx. 1 ½ hours)	*Dynaport MoveMonitor* (lower back; NR; NR; pattern recognition approach using logical algorithms)	Posture/Activity Type (lying, sitting, locomation); Intensity (Steps)	Observation (video)	Intraclass correlation coefficient; Agreement; Sensitivity; Specificity; Predictive values	MoveMonitor provides accurate information on a diverse set of postures; daily activities; and number of steps in IC patients	High	N/N
74	Full et al. (2018, United States)	Community-dwellig adults (N=17; 58.47±7.0 yrs; 59% females; White (N=15), Hispanic (N=2)	1 day (1 night)	*ActiGraph GT3X+* (non-dominant wrist, dominant wrist, dominant hip; ActiLife v6.11; 30 sec; Cole-Kripke algorithm, 1000 cpm)	Biological state (Total sleep time)	Polysomnograp hy	Paired t-test; Bland-Altman analysis	PA accelerometer devices worn on either wrist provide valid estimates of TST, WASO, and SE when compared with PSG.	Some	N/N
75	Fuller et al. (2017, Australia)	Male elite team-sport athletes (N=21; 22.5±2.7 yrs; 0% females; NR)	4 days	*Actical Z series* (non-dominant wrist; Actiware 5.61 activity and sleep analysis software; 1 minute; Acticalthreshold sensitivities (Low, Medium and High))	Biological state (Sleep time)	Polysomnograp hy	Mean bias; 95% CI; Pearson correlation; Standard error of estimate; Bland-Altman analysis	Sleep parameters measured by the Actical® device are greatly influenced by the sleep-wake threshold applied. In the present study the Medium threshold (8.5 min) produced the smallest bias for most parameters compared with PSG.	Low	N/NR
76	Fullerton et al. (2017, United Kingdom)	Adults (N=10; 23.1±1.7 yrs; 20% females; NR)	1 day	*Runscribe inertial sensors* (left ankle, right ankle, left hip, right hip, left upper arm, right upper arm, left wrist, right wrist, spine; NR; Support vector machines)	Posture/Activity Type (Cycling, running, inactive, walking, home activities)	Observation (images)	Accuracy	Results show that recognition of activity and sub-category activity types is possible in a free-living environment thourough the use of multiple body worn accelerometers.	Some	N/NR
77	Gardner & Poehlman, (1998, United States)	Peripheral arterial occlusive disease patients with intermittent claudiation (N=22;	2 days	*Caltrac accelerometer* (hip; NR; NR; equation reported)	Intensity (Total energy expenditure)	Doubly labelled water	Pearson correlation; Spearman correlation; Paired t-tests	Free-living daily physical activity of older PAOD patients with intermittent claudication can be accurately predicted with an accelerometer, and to a lesser extent	Some	N/NR

		69±7 yrs; 9% females; Caucasians (N=10); African-Americans (N=12)						with a pedometer, worn over a 48-h period.		
78	Garnotel et al. (2020, France)	Adults (N=56; 39.6±12.7 yrs; 48% females; NR)	14 days	*ActiGraph GT3X* (hip; Actiheart software v4.0; 1 minute; Freedson VM3 combination model, single linear model, automatic-activity-recognition-based model)	Intensity (Total energy expenditure)	Doubly labelled water	Bland-Altman analysis; Paired t-test; ANOVA; Predicted R squared; Root mean squared error	The AAR model resulted in a 43% increase of daily PAEE variance explained by accelerometry predictions.	Low	N/N
79	Gill et al. (2018, United Kingdom)	Middle-aged male soccer fans (N=21; 30-65 yrs; 0% females; NR)	7 days	*SitFIT* (front trouser pocket; NR; NR; algorithm reported)	Posture/Activity Type (sitting, lying, upright); Intensity (Steps)	Wearable (ActivPAL (thigh))	Bland-Altman analysis; Pearson correlation	The SitFIT has excellent validity for measurement of free-living step counts and sedentary time and therefore addresses a clear need for a device that can be used as a tool to provide feedback on sedentary behaviour to facilitate behaviour change.	High	N/N
80	Godfrey et al. (2007, Ireland)	Adults (N=10; 24.9±1.69 yrs; 30% females; NR)	< 1 day (approx. 6 hours)	*ActivPAL* (thigh; activPAL interface program software; 15 sec; NR)	Posture/Activity Type (sitting/lying, standing, stepping)	Wearable (Analog Devices ADXL202 (thigh, chest))	Accuracy	In a population of healthy adults, the data obtained from the activPAL Professional physical activity logger for both static and dynamic activities showed a close match to a proven discrete accelerometer data with an offset of approximately 2% between the two systems.	High	N/NR
81	Gomersall et al. (2016, Australia)	University staff (N=29; 39.6±11 yrs; 90% females; NR)	7 days	*Fitbit One* (belt, bra, pocket; NR; NR; NR); *Jawbone UP* (wrist; NR; NR; NR)	Intensity (Steps; Time spent in different intensities (MVPA, sedentary))	Wearable (ActiGraph GT3X+ (hip))	Pearson correlation; Spearman correlation; Bland-Altman analysis; Linear regression; Cohen's kappa	There was moderate-strong agreement between the ActiGraph and both Fitbit One and Jawbone UP for the estimation of daily steps.	High	N/N
82	Gruwez et al. (2017, Belgium)	Adults (N=20; 30±5 yrs; 65% females; NR)	1 ½ days	*Jawbone Up Move* (wrist; NR; NR; NR); *Withings Pulse O2* (wrist; NR; NR; NR); *SenseWear Pro* (non-dominant upper arm; NR; NR)	Intensity (Steps, Energy expenditure); Biological state (Total sleep time)	Wearable (SenseWear Pro (non-dominant upper arm); Polysomnograp hy	Signed rank test; Spearman correlation; Bland-Altman analysis	Sleep and activity monitors are only able to produce a limited set of reliable measurements, such as TST, step count, and active EE, with a preference for U which performs globally better.	High	N/N
83	Haghayegh et al. (2019, United States)	Adults (N=35; 27.2±12.6 yrs; 48.5% females; Primarily Caucasian)	1 day (1 night)	*Motionlogger Micro Watch* (non-dominant wrist; NR; 30 sec; unique proprietary scoring interpretative algorithm); *Fitbit Charge 2* (wrist; proprietary software algoritm of Fitbit	Biological state (Total sleep time)	Zmachine portable EEG	Paired t-tests; Bland-Altman analysis; Linear regression; Minimum detectable change; Cohen´s kappa	Performance of Fitbit accelerometry and HRV technology in conjunction with its proprietary IA to detect sleep vs. wake episodes is slightly better than wrist actigraphy that relies solely on accelerometry and best performing Sadeh IA.	High	N/N

				Company; 30 sec; proprietary algorithm)						
84	Hamill et al. (2019, Australia)	Insomnia diagnosed participants (N=25; 50.6±15.9 yrs; 56% females; NR)	7 days	*Fitbit Alta HR* (non-dominant wrist; NR; 30 sec; proprietary algorithm)	Biological state (Total sleep time)	Wearable (Actiwatch Spectrum Pro (non-dominant wrist))	Linear mixed models; Pearson correaltion; Spearmans correaltion; Sensitivity; Specificity; Accuracy	Fitbit provides similar estimates of sleep outside the laboratory to a research grade actigraph.	High	N/Y
85	Hargens et al. (2017, United States)	Adults (N=22; 30.8±11.4 yrs; 67.18% females; NR)	7 days	*Fitbit Charge* (non-dominant wrist; NR; NR; MVPA ≥3 METs); *Fitbit One* (right pants pocket or right waist; NR; NR; MVPA ≥3 METs)	Intensity (Steps; Time spent in MVPA; Energy expenditure)	Wearable (ActiGraph GT3X+ (right waist))	Paired t-tests; Bland-Altman analysis; Mean absolute percent error; Mean absolute deviation	Commercial devices are less accurate in estimating steps and EE.	High	N/N
86	Härtel et al. (2011, Germany)	Patients with back pain (N=9; 46.4±10.9 yrs; 0% females; NR)	< 1 day (approx. 1.5 hours)	*kmsMove-sensor* (hip; NR; 1 sec; equation reported)	Intensity (Energy expenditure)	Indirect calorimetry	Intraclass correlation coefficient; Bland-Altman analysis	These findings indicate that the kmsMovesensor is an appropriate measuring device with relatively good accuracy to assess human energy expenditure in rehabilitation patients.	High	NR/NR
87	Henriksen et al. (2019, Norway)	Adults (N=50; 45.1±15.5 yrs; 48% females; NR)	1 day	*Polar M430* (wrist; Polar Flow; 24 hours; proprietary algorithm)	Intensity (Time spent in different intensities (sedentary, LPA, MVPA); Steps; Energy expenditure)	Wearables (ActiGraph (wrist; hip); Actiheart (chest))	Pearson correlation; Spearman correaltion; Intraclass correlation coefficient; Mean absolute percentage error; Bland-Altman analysis; Sensitivity; Specificity	The Polar M430 can potentially be used as an addition to established research-grade instruments to collect some PA variables over a prolonged period.	High	N/N
88	Hernandez-Vicente et al. (2016, Spain)	Adults (N=22; 20.0±1.2 yrs; 50% females; Caucasian (N=22))	7 days	*Polar V800* (wrist; NR; NR; NR)	Intensity (Energy expenditure; Steps; Time spent in different intensities (sedentary, physically active))	Wearables (ActiGraph (right hip); ActiTrainer (right hip))	Paired t-tests; Pearson correaltion; Bland-Altman analysis; Sensitivity; Specificty	Polar V800 accelerometer has a comparable validity to the accelerometer in free-living conditions; regarding "1-hour sedentary bouts" and "V800's walking time vs. ActiTrainer's lifestyle time" in young adults.	High	NR/N
89	Herrmann et al. (2011, United States)	Employees of a university campus (N=16; 40.2±12.6 yrs; 50% females; NR)	7 days	*MyWellness Key* (right waist; proprietary MyWellness Key web-based software; NR; light: 1.8–2.9 METs, moderate: 3–5.9 METs, vigorous: 6+ METs)	Intensity (Time spent in different intensities (LPA, MVPA))	Wearables (ActiGraph GT1M (left hip); Yamax Digiwalker SW-200 (hip))	Spearman correlation	The MyWellness Key has a high concurrent validity with the ActiGraph accelerometer to detect PA in both controlled laboratory and free-living settings.	High	Y/N
90	Hickey et al. (2017, United Kingdom)	Adults (N=10; 27.5±4.7 yrs; NR; NR)	< 1 day (two 1 hour sessions)	*AX3* (lower back; NR; NR; detection and segmentation algorithm)	Intensity (Steps)	Observation (video)	Spearman correlation; Intraclass correlation coefficient; Wilcoxon	The algorithm employed for identifying and quantifying steps and bouts from a single wearable accelerometer worn on the lower-	High	N/NR

							test; Bland-Altman analysis	back has been demonstrated to be valid and could be used for pragmatic gait analysis in prolonged uncontrolled free-living environments.		
91	Hickey et al. (2016, United States)	Adults (N=15; 24.9±5.1 yrs; 53.3% females; NR)	1 day	*Omron HJ720-ITC* (waist; NR; NR; NR); *Yamax Digiwalker SW-200* (waist; NR; NR; NR); *ActiGraph GT3X* (each hip; NR; NR; NR); *ActivPAL* (thigh; NR; NR; NR)	Intensity (Steps)	Wearable (StepWatch (ankle))	Linear mixed model; Bias; Percentage error	This study highlights the need to verify step-counting accuracy of activity monitors with activities that include different movement types/directions.	High	N/NR
92	Höchsmann et al. (2021, Switzerland)	Adults (N=30 median age 25 yrs; 60% females; NR)	3 days	*Garmin Vivofit 2* (non-dominant wrist; NR; NR; NR); *ActiGraph wGT3X+* (non-dominant wrist; opposite hip; ActiLife v6.13.3; 1 minute; NR)	Intensity (Steps)	Wearable (StepWatch 3 (right ankle))	Intraclass correlation coefficient; Mean absolute percentage error; Bland-Altman analysis	The overall high MAPE of the devices compared to the StepWatch during step-based activities, likely caused by inaccuracies during short and intermittent bouts of activity, may limit their validity in a free-living setting.	High	N/N
93	Hollewand et al. (2016, Netherlands)	Elderly subjects of residential care facility (N=18; 83.4±5.0 yrs; 77.8% females; NR)	< 1 day (6 hours)	*Dynaport MoveMonitor MicroMod* (lower back; McRobertst web service; 1 minute; NR)	Posture/Activity Type (standing, sitting, locomotion, lying)	Observation (direct)	Cohen´s kappa; Sensitivity; Specificity; Agreement	DynaPort system is not a valid method for assessing physical activity in frail elderly subjects	High	N/N
94	Hui et al. (2018, Canada)	Participants with stroke (N=12; 62.6±9.3 yrs; 42% females; NR)	3 days	*Fitbit One* (non-paretic ankle; NR; 1 minute; proprietary algorithms)	Intensity (Steps; Time spent in different intensities (sedentary, LPA, MVPA))	Wearable (Actical (non-paretic ankle))	Relative error; Regression analysis; Bivariate correlation	The Fitbit One was associated with the Actical accelerometer in measuring step count and light-intensity activity during free-living activity after stroke, but had lower error in capturing step count for those with faster walking speeds.	High	N/N
95	Jean-Louis et al. (2001, United States)	Postmenopausal women (N=39; 63.72±7.35 yrs; 100% females; NR)	1 day (1 night)	*Actillume* (wrist; ACTION3; 1 minute; NR)	Biological state (Sleep-wake scores)	Polysomnograp hy	Percentage agreement; Pearson correlation; Measurement error	Strong correaltion and agreements between sleep estimates from Actillume and PSG suggest that the Actillume can reliably monitor sleep and wakefulness	Some	N/NR
96	Jenkins et al. (2021, Australia)	Adults (N=33; 25.33±4.69 yrs; 60.6% females; NR)	7 days (7 nights)	*GENEActiv* (wrist; GENEActiv PC software v3.2; 1 minute; Phillips Respironics Algorithm)	Biological state (Total sleep time)	Wearable (Actiwatch-2 (wrist))	Sensitivity; Specificity; Accuracy; Bland-Altman analysis; Paired t-tests	Overall; the findings from this study provide the first empirical evidence to support the reliability of GENEActiv against Actiwatch-2 over multiple nights using a common algorithm with device-specific wake thresholds.	High	NR/N
97	Johannsen et al. (2010, United States)	Adults (N=30; 38.2±10.6 yrs; 50% females; 73% White;	14 days	*SenseWear Pro 3* (right upper arm; software v6.1; NR; algorithm V.2.2.3); *SenseWear*	Intensity (Total energy expenditure)	Doubly labelled water	Agreement; Paired t-test; Linear regression; ANCOVA; Intraclass correlation coefficient	The SenseWear Pro3 and the SenseWear Mini armbands show promise for accurately measuring	Low	Y/NR

		20% Hispanic; 7% Asian)		*Mini* (left arm; software v7.0; NR; algorithm V.2.2.4))				daily energy expenditure under free-living conditions.		
98	Johansson et al. (2006, Sweden)	Adults (N=8; 38.5±14.4 yrs; 25% females; NR)	14 days	*MTI ActiGraph* (lower back; NR; 15 sec; equation reported)	Intensity (Total energy expenditure)	Doubly labelled water	Bland-Altman analysis; Root mean squre error	The HR–ACC combination as well as ACC alone has potential as a method for assessment of TEE during free-living activities as compared with DLW.	High	N/N
99	Judice et al. (2015, Portugal)	Adults (N=7; 49.7±12.6 yrs; 71% females; NR)	14 days	*ActiGraph GT3X+* (right hip; Actilife v6.9.1; 15 sec; NR); *Actiheart* (chest; Actiheart and camNtech software; 15 sec; NR)	Posture/Activity Type (Sedentary time; Sedentary breaks)	Wearable (ActivPAL (thigh))	Paired samples t-test; Coefficient of derermination; Standard error of estimate; Concordance correlation coefficient; Bland-Altman analysis	The present results highlight the magnitude of potential errors in estimating sedentary time and breaks from common alternative methods other than ActivPAL.	High	N/N
100	Judice et al. (2019, Portugal)	Adults with overweight/obesity (N=10; 50.4±11.4 yrs; 50% females; NR)	7 days	*ActiGraph GT3X+* (right hip; Actilife v6.9.1; 15 sec; NR)	Posture/Activity Type (Sedentary time; Postural transitions)	Wearable (ActivPAL (thigh))	Paired samples t-test; Coefficient of derermination; Standard error of estimate; Concordance correlation coefficient; Bland-Altman analysis	Findings suggest that at the group level; AGincl provides similar estimates compared to AP for SB and standing-time; but not for SB-to-upright transitions or stepping-time.	High	N/N
101	Kanda et al. (2012, Japan)	University staff (N=4; 30.3±3.0 yrs; 25% females; Japanese (N=4)	NR	*DynaPort Activity Monitor* (waist, left leg; DynaScope software McRoberts; NR; NR)	Posture/Activity Type (locomotion; standing, sitting)	Self-reported activity diary	Intraclass correlation coefficient; Bland-Altman analysis	The triaxial accelerometer is reliable for evaluating the physical activity of Japanese COPD patients.	High	NR/N
102	Kang et al. (2017, South Korea)	Insomnia disorder patients (N=33; 38.4±11.2 yrs; 57.6% females; NR); Good sleepers (N=17; 32.1±7.4 yrs; 64.7% females; NR)	1 day (1 night)	*Fitbit Flex* (non-dominant wrist; NR; 1 minute; NR); *Actiwatch 2* (non-dominant wrist; Actiware software v6.0.8; 30 sec; NR)	Biological state (Total sleep time)	Polysomnograp hy	Intraclass correlation coefficient; Bland-Altman analysis; Repeated-measures ANOVA; Paired t-test; Wilcoxon test; Sensitivity; Specifity; Accuracy	The ICC of TST in the FFN–PSG comparison was excellent in both groups; and the frequency of agreement was high in good sleepers but significantly lower in insomnia patients.	High	N/NR
103	Kawada (2008, Japan)	University students (N=76; 24.7±1.9 yrs; 28.9% females; NR)	5 days	*Actiwatch Mini Mitter* (non-dominant wrist; NR; 1 minute; cutoff point 40)	Biological state (Sleep/wake times)	Self-reported sleep diary	Agreement	The accelerometer showed some measurement failure during waking; presumably because of the decrease in body movement.	High	NR/NR
104	Keating et al. (2012, United States)	College students (N=17; 20.35±0.86 yrs; 76.5% female; Chinese (N=17))	< 1 day (10 x 90 min)	*Fitbit Charge 2* (non-dominant wrist; NR; NR; NR)	Intensity (Steps)	Wearable (Yamax SW-200 (hip))	Linear mixed-model analysis	FC2 revealed promising validity evidence measuring total steps in a controlled setting and its validity may be questionable in college physical education settings where student physical activity patters are irregular.	High	NR/NR

105	Kerr et al. (2013, United States)	Adult cyclists (N=40; 36±12 yrs; 30% females; 85% Caucasian)	3-5 days	*ActiGraph GT3X+* (hip; ActiLife 6.2.1; 1 minute; 100-cpm cutpoint)	Posture/Activity Type (sedentary, standing still, standing/movin g, biking)	Observation (Images)	Sensitivity; Specificity	Researchers should be aware of the strengths and weaknesses of the 100-cpm accelerometer cutpoint for identifying sedentary behaviour.	Some	N/NR
106	Kerr et al. (2018, United States)	Breast cancer survivors (N=30; 62±8 yrs; 100% females; NR)	7 days	*ActiGraph GT3X+* (hip; Actilife 6.11; 1 minute; random forest classifier)	Posture/Activity Type (stepping, standing, sitting, sit-to-stand and stand-to-sit transitions)	Wearable (ActivPAL (thigh))	Generalized estimating equations; Sensitivity; Specificity; Accuracy	This is among the first algorithms for sitting and standing for hip-worn accelerometer data to be trained from entirely free-living activPAL data. The new algorithm detected prolonged sitting; which has been shown to be the most detrimental to health.	High	N/N
107	Kim et al. (2015, United States)	Adults (N=11; 30.67±7.24 yrs; 27.3% females; NR)	< 1 day (6 hours)	*ActiGraph GT3X* (waist; Actilife v5.10.0; 1 sec, 10 sec, 1 minute; Sojurn method, < 8 cpm, < 50/100/150 cpm); *ActivPAL* (right thigh; activPAL software v7.1.18; NR; proprietary algorithm)	Posture/Activity Type (Sedentary time)	Observation (video)	Mean absolute percentage error; Mean sensitivity; 1-specificity; Youden's index; Phi coefficient	ActivPAL should be considered for studies focusing on sedentary behaviour.	High	N/N
108	Kinnunen et al. (2012, Finland)	Male conscripts (N=24; 19-20 yrs; 0% females; NR)	7 days	*Polar activity recorder FA20* (non-dominant wrist; NR; 1 minute; equation reported)	Intensity (Energy expenditure)	Doubly labelled water	Multivariate linear regression analysis; Paired-samples t-test; Pearson correlation; Bland–Altman analysis	AR can be regarded as a reliable and valid method for assessing EE during intensive training.	Some	Y/NR
109	Kinnunen et al. (2019, Finland)	Adults (N=15; 30±6 yrs; 0% females; NR)	14 days	*Polar Active* (wrist; Polar web service; 30 sec; NR); *Polar RS800CX* (wrist; NR; 30 sec; NR)	Intensity (Energy expenditure)	Doubly labelled water	Pearson correlation; Bland–Altman analysis	Wrist motion sensor combined with heart rate monitor during exercise sessions showed high agreement with golden standard measurement of daily TEE.	Some	Y/N
110	Koehler et al. (2011, Germany)	Endurance athletes (N=14; 30.4±6.2 yrs; 0% females; NR)	7 days	*SenseWear Pro3 Armband* (right arm; SenseWear Professional software v6.1; 1 minute; proprietary algorithm)	Intensity (Total energy expenditure)	Doubly labelled water	Correlation coefficient; Bland–Altman analysis	SWA does not provide valid results of TEE and ExEE in endurance athletes because of the underestimation of EE at higher exercise intensities	High	N/N
111	Koenders et al. (2018, Netherlands)	Male students (N=27; mean 22 yrs; 0% females; NR)	< 1 day (30 min)	*HealthPatch* (chest; NR; 1 sec; NR)	Posture/Activity Type (lying, sitting, standing, walking)	Observation (video)	Intraclass correlation coefficient; Bland Altman analysis	Good validity of the HealthPatch to monitor lying and poor validity to monitor sitting/standing or walking. In addition; the validity outcomes were less favourable in the hospital setup.	High	N/N
112	Kogure et al. (2011, Japan)	Adults (N=6; 32.2±3.9 yrs; 66.67% females; NR)	2 days	*Mini Motionlogger Actigraph* (non-dominant	Biological state (Total sleep time)	Polysomnograp hy	Agreement; Sensitivity; Specificity	All our validation results indicate that the NWA device, placed under a mattress or a futon, can produce	High	NR/NR

				wrist; NR; 1 minute; Cole-Kripke algorithm)				almost identical sleep/wake scores to Actigraph.		
113	Kooiman et al. (2015, Netherlands)	Adults (N=56; Males: 37.1±10.6 yrs, females: 30±9.5 yrs; 67.9% females; NR)	< 1 day (6.5 hours)	*Lumoback* (lower back; NR; NR; NR); *Fitbit Flex* (wrist; NR; NR; NR); *Nike+ Fuelband SE* (wrist; NR; NR; NR); *Jawbone Up* (wrist; NR; NR; NR); *Misfit Shine* (trousers front pocket; NR; NR; NR); *Withings Pulse* (trousers front pocket; NR; NR; NR); *Fitbit Zip* (trousers front pocket; NR; NR; NR); *Omron HJ-203* (trousers front pocket; NR; NR; NR); *Yamax Digiwalker SW-200* (waist; NR; NR; NR)	Intensity (Steps)	Wearable (ActivPAL (thigh))	Intraclass correlation coefficient; t-test; Bland-Altman analysis	Validity of eight of the ten trackers was good. Fitbit Zip showed the highest validity; Nike+ Fuelband indicated a low validity.	High	N/N
114	Kozey-Keadle et al. (2011, United States)	Overweight office workers (N=20; 46.5±10.7 yrs; 75% females; NR)	< 1 day (6 hours)	*ActivPAL* (right thigh; NR; 15 sec; NR); *ActiGraph GT3X* (right hip; NR; 1 sec; 50, 100, 150, 200, 250 cpm)	Posture/Activity Type (lying, sitting)	Observation (direct)	Repeated-measures linear mixed model; Correlation and confidence intervals; Bland-Altman analysis	The AP was more precise and more sensitive to reductions in sitting time than the AG. And thus; studies designed to assess SB should consider using the AP	High	N/N
115	Kubala et al. (2020, United States)	Adults (N=30; 24.8±4.1 yrs; 50% females; 87% White)	7 days	*Fitbit Alta* (non-dominant wrist; App/software v2.38.1; NR; NR); *Jawbone Up3* (non-dominant wrist; App/software v4.29.0.100; NR; NR); *Misfit Shine 2* (non-dominant wrist; App/software v2.15.2; NR; NR); *Polar A360* non-dominant wrist; App/software v3.5.4; NR; NR); *Samsung Gear Fit2* (non-dominant wrist; App/software v1.6.17030904; NR; NR); *Xiaomi Mi Band 2* (non-dominant wrist; App/software v3.0.4; NR; NR)	Biological state (Total sleep time)	Wearable (Actiwatch Spectrum Classic (wrist))	Intraclass correlation coefficient; T-test; Bland-Altman analysis	Agreement between commercial activity monitors and Actiwatch varied by device; with greater agreement observed for total sleep time than wake after sleep onset.	High	N/NR

116	Kumahara et al. (2015, Japan)	Adults (N=31; 21.6±1.1 yrs; 100% females; NR)	1 day	*Kenz e-style2; Tanita Calorism Smart; Omron CaloriScan HJA-306* (all pants pocket, shoulder bag; NR; NR; NR)	Intensity (Steps; Time spent in MVPA; Energy expenditure)	Wearable (Lifecorder (waist))	Pearson correlation; Bland–Altman analysis	Monitors placed at the PP location; especially the Kenz monitor; showed acceptable accuracy for young adult women in real-life settings. In contrast; MVPA indices assessed using these monitors showed limited validity	High	N/N
117	Kurita et al. (2017, Japan)	Adults (N=43; 42.5±12.2 yrs; 41.8% females; NR)	2 days	*Active style Pro HJA-350IT* (waist; Omron health management software BI-LINK for physical activity professional edition v1.0; 10 sec, 1 minute; NR); *ActiGraph GT3X+* (waist; Actilife v6.10.4; 1 minute; 100, 150 cpm)	Posture/Activity Type (Sedentary time; sedentary breaks)	Wearable (ActivPAL3 (thigh))	Repeated ANOVA; Pearson correlation; Bland-Altman analysis	Compared to the AP as the criterion; the ASP can underestimate total sedentary time and the GT3X+ can overestimate it; and more so at the lower levels of sedentary time.	High	N/N
118	Kwon et al. (2021, United States)	Pacific Islanders (N=20; 32.5±15.1 yrs; 60% females; Tongan Americans (N=10); Samoan Americans (N=10))	7 days	*MotionSense HRV* (non-dominant wrist; mCerebrum mobile application; 1 minute; NR)	Intensity (Time spent in different intensities (sedentary, physically active))	Wearable (ActiGraph GT9X Link (wrist))	Pearson correlation; Bland–Altman analysis	MotionSense HRV yielded comparable estimates for SED and PA when compared with the GT9X accelerometer under free-living conditions.	High	N/N
119	Latshang et al. (2016, Switzerland)	Adults (N=51; 27±9 yrs; 0% females; NR)	5 days (5 nights)	*MSR2005* (wrist; Respironics Actiware 5; 1 minute; NR); *MSR2010* (wrist, ankle; Respironics Actiware 5; 1 minute; NR)	Biological state (Total sleep time)	Polysomnography	Bland-Altman analysis; Spearman correlation; Friedman ANOVA; Wilcoxon test; Mann-Whitney U tests	Actigraphy of the wrist or ankle by a one-axis or a three-axis device accurately estimates mean TST in groups of subjects and mean TST over several nights in individuals traveling to altitude.	Some	N/N
120	Le Masurier et al. (2004, United States)	Adults (N=12; Males: 30.5±6.6 yrs, females: 27.7±6.3 yrs; 50% females; NR)	1 day	*Yamax SW-200; Omron HJ-105; Sportline 330* (all waist; NR; NR; NR)	Intensity (Steps)	Wearable (CSA accelerometer 7164 (waist))	One-way ANOVA; Accuracy	Different brands of motion sensors detect steps differently; therefore, caution must be used when comparing step counts between studies that have employed different brands of motion sensors.	High	N/N
121	Lebleau et al. (2020, Belgium)	Adults (N=60; 39.4±12 yrs; 48% females; NR)	1 day	*Nokia Gos* (each wrist; non-dominant hip; NR; NR; NR)	Intensity (Steps); Biological state (Total sleep time)	Wearable (ActiGraph wGT3X-BT (non-dominant wrist)	Intraclass correlation coefficient; Bias; Bland-Altman analysis	There are high discrepancies in step count between devices because of the different types of activities in daily life.	High	NR/NR
122	Lee & Laurson (2015, United States)	Students (N=35; Males: 22.8±2.2 yrs, females: 21.3±1.7 yrs; 51% females; NR)	3 days	*SenseWear Pro3* Armband (right arm; NR; 1 minute; NR)	Intensity (Steps)	Wearable (Yamax Digiwalker SW-701 (right hip))	Intraclass correlation coefficient; Paired t-test; Bland-Altman analysis	The SWA underestimates steps during treadmill walking and appears to overestimate steps during free-living compared to the DIGI pedometer.	High	N/N

123	Lee & Suen (2017, Hong Kong)	Adults (N=49; 18-64 yrs; 51% females; NR)	7 days	*ActiGraph GT9X Link* (non-dominant wrist; NR; 1 minute; Cole-Kripke algorithm, Sadeh algorithm, Sazonov algorithm, high sensitivity threshold (<40 counts), medium sensitivity threshold (<20 counts), low sensitivity threshold (<10 counts) [10], neural network model)	Biological state (Total sleep time)	Wearable (Actiwatch 2 (non-dominant wrist))	Spearman correlation; Paired t-test; Cohen´s d; Bland-Altman analysis	Results showed that the sleep function of the ActiGraph Link performs similar to a validated accelerometer (Actiwatch 2) and provides an opportunity to measure both sleep and PA simultaneously.	High	N/N
124	Lee et al. (2018, United States)	Adults (N=78; 27.6±11 yrs; 54% females; 71% Caucasian, 22% Asian/Pacific Islander, 6% Hispanic/Latino, 1% African American)	4 days (3 nights)	*ActiGraph GT9X Link* (wrist; ActiLife software 6.5.3; NR; Sadeh and Cole-Kripke sleep algorithm); *SenseWear Mini Armband* (upper arm; SenseWear software v8.1; 1 minute; NR); *Basis Peak* (wrist; application v1.20.1; NR; NR); *Fitbit Charge HR* (wrist; application v2.15.1; NR; NR); *Jawbone UP3* (wrist; application v1.2.14; NR; NR); *Garmin Vivosmart* (wrist; application v3.90; NR; NR)	Biological state (Total sleep time)	Self-reported sleep diary	Pearson correlation; Glass´delta; Mean absolute percentage error; Bland-Altman analysis	Some of the wearable trackers resulted in closer approximations to self-reported sleep outcomes than a previously sleep research-grade device, these trackers offer a lower-cost alternative to tracking sleep in healthy populations.	High	N/N
125	Lee et al. (2018, South Korea)	Patients with diagnosis of stroke (N=24; 59.7±14.6 yrs; 30% females; NR)	3 days	*ActiGraph wGT3X-BT* (wrists, ankles; ActiLife v6.8.2; 15 sec; Freedson Combination ('98) and Work-Energy Theorem)	Intensity (Energy expenditure)	Observation (direct)	Spearman correlation; Mann Whitney U test; Bland-Altman analysis	Combined models of accelerometers showed higher correlation with direct observation than separate ones.	High	N/N
126	Lee et al. (2015, United States)	College-aged adults (N=37; Males: 21.6±2.8 yrs, females: 21.1±2.5 yrs; 48% females; NR)	3 days	*Omron HJ-720 IT pedometer* (left hip; NR; NR; NR); *ActiGraph GT3X* (right hip; NR; NR; NR); *Polar Active* (left wrist; NR; NR; NR)	Intensity (Steps)	Wearable (Yamax Digiwalker SW-701 (right hip))	Intraclass correlation coefficient; Concordance coefficient; Repeated-measures ANOVA; Bland-Altman analysis	The Omron pedometer seems to provide the most reliable and valid estimate of steps taken, as it was the best performer under lab-based conditions and provided comparable results to the YX in free-living.	High	NR/NR
127	Leenders et al. (2006, United States)	Adults (N=13; 25.8±1.6 yrs; 100% females; NR)	7 days	*Tritrac-R3D* (waist; NR; 1 minute; equations reported); *ActiGraph 7164* (waist; NR; 1 minute; equations reported)	Intensity (Total energy expenditure)	Doubly labelled water	Pearson correlation; Concordance correlation coefficient; One-sample t-test; Bland-Altman analysis	Of the 14 different regression equations from the literature, only two developed for ACT compared favorably with DLW; however, the difference in TDEE between these two methods was variable and	Some	N/NR

								rather large. These results reemphasize the difficulty in converting body movement into energy expenditure on an individual basis from accelerometry		
128	Li et al (2020, United States)	Adults (N=43; mean age 69, range 56-87 yrs; 51% females; White (N=28); Chinese American (N=3); African American (N=8); Hispanic (N=4))	7 days (1 night)	*Actiwatch spectrum* (non-dominant wrist; NR; 30 sec; Hidden Markov Model)	Biological state (Sleep/wake identification)	Polysomnography	Accuracy; Sensitivity; Specificity; Positive predictive value; Cohen´s kappa; Pearson correlation; paired t-tests; Bland-Altman analysis	Unsupervised data-driven HMM algorithm achieved better performance than the commonly used Actiwatch software algorithm and the pre-trained UCSD algorithm.	High	NR/N
129	Liu et al. (2015, Canada)	Adults (N=35; 59±1.6 yrs; 65% females; NR)	1 day	*Lifesource XL-18 pedometer* (right/left front pocket; NR; NR; NR)	Intensity (Steps)	Wearable (Yamax SW-200 (waist))	Absolute percentage error; Percentage error; Sample t-tests; Bland-Altman analysis; Paired t-tests; Intraclass correlation coefficient; Regression analysis	XL-18 is suitable for measuring steps in controlled and free-living conditions. However; caution may be required when interpreting the steps recorded under slower speeds and free-living conditions.	High	NR/NR
130	Löf et al. (2013, Sweden)	Adults (N=22; 36±8 yrs; 100% females; NR)	14 days	*Actiheart* (chest; Actiheart software v4.0.11; NR; branched equation); *IDEEA* (each thigh; each foot; chest; NR; 1 minute; NR); *RT3* (right hip; Stayhealthy RT3 Assist v1.0.7; 1 minute; NR)	Intensity (Activity energy expenditure)	Doubly labelled water	Repeated-measures ANOVA; Pearson correlation; Linear regression; Bland-Altman analysis	The Actiheart may be useful for groups and the RT3 for individuals while the IDEEA requires further development	Some	N/N
131	Lyden et al. (2012, United States)	Adults (N=13; 24.8±5.2 yrs; 69% females; NR)	2 days	*ActiGraph GT3X* (waist; NR; 1 sec; NR); *ActivPAL* (thigh; activPAL software v5.8.5; 1 sec; NR)	Posture/Activity Type (Sedentary time; Sedentary breaks)	Observation (direct)	Repeated-measures linear mixed model; Percentage bias or mean difference	AG-LFE and AG-Norm were not accurate in estimating break rate or the absolute number of breaks and were not sensitive to changes between conditions. ActivPAL is a valid tool to measure components of sedentary behaviour	High	N/Y
132	Lyden et al. (2017, United States)	Adults (N=13; 24.8±5.2 yrs; 62% females; NR)	3 days	*ActivPAL* (thigh; ActivPAL software v5.8.5; 1 sec; < 1.5 MET (sedentary), 1.5-2.99 (light), >2.99 (MVPA))	Intensity (Time spent in different intensities (sedentary, MVPA))	Observation (direct)	Repeated-measures linear mixed model; Root-mean square error; Intraclass correlation coefficient; Bland-Altman analysis	The AP can be used to accurately capture individualized estimates of active and sedentary behaviour variables in free-living settings.	High	N/N
133	Mackey et al. (2011, United States)	Adults (N=19; 82.0±3.3 yrs; 42% females; 13.68% White)	14 days	*SenseWear Pro Armband* (right upper arm; InnerView Professional Research Software v5.1;	Intensity (Total energy expenditure; Activity energy expenditure)	Doubly labelled water	Paired t-tests; Pearson correlation; Intraclass correlation coefficient; Bland-Altman analysis	Acceptable levels of agreement were observed between SWA and criterion measurements of TEE and AEE in older adults.	Some	N/NR

				1 minute; proprietary algorithm)						
134	Madrid-Navarro et al. (2019, Spain)	Patients with Parkinson disease and control (N=30; Parkinson disease: 65.53±2.19 yrs; control: 60.71±1.97 yrs; 20% females; NR)	1 day (1 night)	*Kronwise 3.0* (wrist; Kronoware 10.0 software; 30 sec; TAPL algorithm)	Biological state (Total sleep time)	Polysomnography	Paired t-test; Pearson correaltion; Bland-Altman analysis	The ACM device has proven to be clinically useful to evaluate sleep in an objective manner; thanks to the integrated management of different complementary variables; having advantages over conventional actigraphy.	Some	N/N
135	Manuta et al. (2016, United States)	Adults (N=40; 22.37±4.92 yrs; 48% females; NR)	1 day (1 night)	*Fitbit Flex* (wrist; NR; NR; NR); *Misfit Shine* (wrist; NR; NR; NR); *Withings Pulse O2* (wrist; NR; NR; NR); *Actiwatch Spectrum* (wrist; Actiware v6; 15 sec; default algorithm)	Biological state (Total sleep time)	Polysomnography	Bland-Altman analysis; Wilcoxon test; Pearson correlation	For all devices; we found no difference and strong correlation of total sleep time with PSG. Sleep efficiency differed from PSG forWithings; Misfit; Fitbit; and Basis; while Actiwatch mean values did not differ from that of PSG.	High	N/N
136	Marcotte et al. (2020, United States)	Adults (N=48; 20.4±1.3 yrs; 56.5% females; NR)	< 1 day (4 hours)	*ActiGraph GT3X-BT* (non-dominant wrist, right hip; ActiLife v6.13.3; 1 sec, 1 minute; processing methods reported)	Intensity (Time spent in sedentary)	Observation (video)	Linear mixed models; Mean absolute percent error; Sensitivity; Specificity	Accurate group-level estimates of SB from a hip-worn AG can be achieved using either simpler count-based approaches (CPM100 and CPM150) or machine learning models (Soj1x and Soj3x).	High	N/N
137	Matthews et al. (2018, United States)	Adults (N=932; 63.2±5.9 yrs; 50.54% females; NR)	7 days	*ActiGraph GT3X* (hip; NR; 1 sec, 1 minute; Freedson, Sasaki, Crouter2, Soj3x)	Intensity (Energy expenditure); Posture/Activity Type (sitting, lying)	Doubly labelled water; Wearable (ActivPAL (thigh))	Mean differences; T-tests; Error structure	New exposure assessments suitable for use in large epidemiologic studies (ACT24; ActiGraph) were more accurate and had higher correaltion than a traditional questionnaire.	Low	N/N
138	McDevitt et al. (2021, Ireland)	Adults (N=15; Males: 23.4±3.4 yrs, femals: 29±12.6 yrs; 27% females; NR)	2 days	*Verisense* (wrist; Verisense Cloud Platform; 15 sec; Heuristic algorithm)	Biological state (Total sleep time); Intensity (Counts; Time spent in different intensities MVPA, LPA, sedentary))	Wearable (Actiwatch 2 (wrist))	Spearman correlation; Bland–Altman analysis; Sensitivity; Specificity; Accuracy	Our results showed moderate-high agreement of Verisense with Actiwatch 2 for assessing epoch-by-epoch physical activity and sleep; but a lack of agreement for activity classifications.	High	N/N
139	McGinley et al. (2015, Canada)	Adults with type 2 diabetes (N=35; 62.8±7.8 yrs; 40% females; NR)	14 days	*MyWellness Key accelerometer* (left hip, left bra; NR; NR; >3.0 MET)	Intensity (Time spent in MVPA)	Self-reported activity diaries	Spearman correlation; Bland–Altman analysis	Waist-worn MWK measured PA volume accurately; and was acceptably accurate at discriminating between low- and moderate-intensity PA in people with type 2 diabetes. The MWK underestimated PA volume and intensity when worn on a bra.	High	N/Y

140	McVeigh et al. (2021, Australia)	Adults (N=59; 48±11 yrs; 71.2% females; White (N=50); Aboriginal (N=1); Asian (N=4); Mixed (N=4))	8 days	*Fitbit Charge 2* (wrist; Fitabase; 1 minute; proprietary algorithm)	Intensity (Time spent in different intensities (sedentary; LPA; MVPA); Steps)	Wearable (ActiGraph GT3X+ (hip))	Pearson correlation; Spearman correlation; Intraclass correlation coefficient; Paired t-test; Bland-Altman analysis; Mean absolute percentage error; ANOVA; Kappa statistics	The Charge 2 may be a useful tool for self-monitoring of SB and PA in an overweight population; as mostly good agreement was demonstrated with the GT3X+.	High	Y/NR
141	Middelweerd et al. (2021, Netherlands)	Adults (N=30; 23.9±3.9 yrs; 66.6% females; NR)	7 days	*Fitbit One* (right hip; NR; 1 minute; proprietary algorithm)	Intensity (Steps; Time spent in MVPA)	Wearable (ActiGraph GT3X+ (waist))	Absolute error percentage; Linear mixed model analysis Intraclass correlation coefficient; Bland–Altman analysis	Although the Fitbit One overestimates the step activity compared with the ActiGraph; it can be considered a valid device to assess steps activity; including for real-time minute-by-minute self-monitoring. However; agreement and correlation between ActiGraph and Fitbit One regarding time spent in moderate; vigorous; and moderate–vigorous physical activity was lower.	High	N/N
142	Mikkelsen et al. (2020, Denmark)	Adults (N=41; 47.6±10.4 yrs; 75.6% females; NR)	7 days	*Fitbit Charge 2* (wirst; firmware v22.53.4; 1 minute; proprietary algorithm)	Intensity (Steps; Time spent in different intensities (sedentary, LPA, MVPA); Activity energy expenditure)	Wearable (ActiGraph GT3X (hip, wrist))	Bland-Altman analysis	Moderate to substantial differences between devices were found for most outputs; which could be due to differences in algorithms.	High	N/N
143	Miyamoto et al. (2018, Japan)	Patients with chouronic obstructive pulmonary disease (N=12; 76.6±6.9 yrs; 8% females; NR)	7 days	*Omron Active Style Pro HJA-750C* (waist; NR; 10 sec; NR)	Intensity (Energy exependiture (MET))	Wearable (DynaPort Move Monitor (waist); Actimarker (waist))	Spearman correlation; Bland–Altman analysis; Wilcoxon test	The HJA was validated for evaluating the PA in patients with COPD.	High	N/N
144	Moore et al. (2012, United Kingdom)	Poststroke participants with mild gait deficit (N=9; 73±8 yrs; 33.3% females; NR)	10 days	*Sense Wear Pro 3* (upper arm; NR; NR; NR)	Intensity (Total energy expenditure)	Doubly labelled water	Mann-Whitney U test; Spearman rank correlation coefficient; Bland-Altman analysis	The multisensor array is a portable and accurate method of capturing daily energy expenditure and may assist in understanding how stroke influences free-living energy expenditure and aid in clinical management.	High	N/N
145	Mouritzen et al. (2020, Denmark)	Adults (N=18; 56.1±1 yrs; 72.2% females; NR)	1 day (1 night)	*Garmin Vivosmart 4* (wrist; Garmin Connect™ accounts; 1 minute; NR)	Biological state (Total sleep time)	Polysomnograp hy	Paired t-tests; Cohen's kappa; Intraclass correlation coefficient; Bland-Altman analysis	GV4 is not able to reliably describe sleep architecture but may allow for detection of changes in sleep onset; sleep end; and TST (ICC ≥ 0.825) in longitudinally followed groups.	Some	N/NR

146	Murakami et al. (2019, Japan)	Adults (N=19; 32.3±9.6 yrs; 52.6% females; NR)	15 days	*Fitbit Flex; Jawbone UP24; Misfit Shine; Epson Pulsense; Garmin vivofit* (all wrist; NR; NR; NR); *Tanita AM-160; Omron CaloriScan HJA-401F* (all pocket; NR; NR; NR); *Withings Pulse O2; Omron Active style Pro; Panasonic Actimarker; Suzuken Lifecorder EX; ActiGraph GT3X* (all waist; NR; NR; NR)	Intensity (Activity energy expenditure)	Doubly labelled water	Dunnett test; Mean absolute percent errors; Pearson and Spearman correlation; Bland-Altman analysis	Most wearable devices do not provide comparable PAEE estimates when using gold standard methods during 15 free-living days.	Some	NR/Y
147	Myers et al. (2014, United States)	Adult patients with abdominal aortic aneurysm (N=24; 71±3 yrs; 10% females; NR)	3 days	*ActiGraph GT1M* (waist; NR; 1 sec; NR); *Omron 720-ITC* (hip; NR; NR; NR)	Intensity (Energy expenditure)	Self-reported activity diary	ANOVA; Intraclass correlation coefficient; Bland-Altman analysis	ACC provided a reasonably accurate reflection of EE based the criterion measure; an activity recall questionnaire.	High	NR/NR
148	Narayanan et al. (2020, New Zealand)	Adults (N=15; 31.5±10.8 yrs; 66.6% females; NR)	< 1 day (2 hours)	*Axivity AX3* (hip, thigh, wrist; OmGui v1.0.0.30; 5 sec; rando forest)	Posture/Activity Type (sitting; lying; standing, walking, running, cycling)	Observation (Video)	Sensitvity; Specificity; Accuracy	This validation study demonstrated that a dual-accelerometer system previously validated in a laboratory setting also performs well in semi free-living conditions.	High	N/N
149	Nguyen et al. (2013, Switzerland)	Adults (N=17; 24.5±5 yrs; 47.1% females; NR)	< 1 day (2 hours)	*Lifecorder Kenz EX* (waist; NR; 1 minute; equation reported); *Step Watch 3 Activity Monitor* (ankle; NR; 1 minute; equation reported)	Intensity (Energy expenditure)	Indirect calorimetry	Pearson correlation; Bland-Altman analysis; Paired t-test	Combining GPS and 2 accelerometers allows for an accurate assessment of PA and EE in free-living situations.	High	N/NR
150	O´Brien et al. (2018, Canada)	Adults (N=39; males: 63.9±10.9 yrs; females: 54.9±10.6 yrs; 71.8% females; NR)	7 days	*PiezoRx*® (left hip; NR; NR; 100 steps/min (MPA), 120 (VPA))	Intensity (Steps; Time spent in different intensities (MVPA, sedentary))	Wearable (ActiGraph GT3X (right hip))	Pearson correlation; Sensitivity; Specificity; Percentage agreement; Bland-Altman analysis	The PiezoRx® appears to be a valid measure of free-living PA compared to accelerometry.	High	Y/NR
151	O´Brien et al. (2020, United Kingdom)	Adults with rheumatoid arthouritis (N=104; 58.5±12.1 yrs; 71% females; NR)	7 days	*ActiGraph GT3X+* (right hip; Actilife; 1 sec; ≤ 244 cpm)	Intensity (Time spent sedentary)	Wearable (ActivPAL3 (thigh))	Bland-Altman analysis	Rheumatoid arthritis specific cut-points offer a validated measure of sedentary time; light-intensity PA and moderate-intensity PA in these patients; and demonstrated superior accuracy for estimating free-living sedentary time; compared to non-rheumatoid arthritis cut-points.	High	N/N
152	O´Neill et al. (2017,	Patients with bronchiectasis (N=55;	7 days	*Digiwalker CW-700* (dominant hip; NR; NR; NR)	Intensity (Steps)	Wearable (ActiGraph GT3X+	Percentage agreement; Bland-Altman analysis	ActiGraph or pedometer could be used to measure simple daily step counts, but ActiGraph was superior	High	N/N

	Northern Ireland)	63±10 yrs.; 60% females; NR)				(dominant side hip))		as it measured intensity of physical activity and was a more precise measure of time spent walking.		
153	Paul et al. (2015, Australia)	Community-dwelling healthy older adults (N=32; 67.7±5.7 yrs; 63%; females; NR)	7 days	*Fitbit (One or Zip)* (right hip; NR; NR; proprietary algorithm)	Intensity (Steps)	Wearable (ActiGraph GT3X+ (right hip))	Percentage agreement; Bland-Altman analysis	The Fitbit accurately tracked steps during the 2MWT, but the ActiGraph appeared to underestimate steps. There was strong agreement between Fitbit and ActiGraph counted steps.	High	N/N
154	Pavey et al. (2016, Australia)	Adults (N=57; 28.11± 7.42 yrs; 48% females; NR)	1 day	*GENEActiv* (wrist; PC software v2.1; 15 sec; NR)	Posture/Activity Type (sitting/lying, standing)	Wearable (ActivPAL 3 (thigh))	Paired t-test; Intraclass correlation coefficient; Pearson correlation; Bland-Altman analysis; Kappa statistics	The estimation of sedentary time by posture classification of the wrist-worn GENEActiv accelerometer was comparable to the activPAL. The GENEActiv may provide an alternative	High	N/NR
155	Pomeroy et al. (2011, United States)	American Indians (N=50; 20-34 yrs.; 50% females; American Indians (N=50))	7 days	*Accusplit-AX120* (hip; NR; NR; NR); *Dynastream-AMP-331* (ankle; NR; NR; pattern recognition algorithm)	Intensity (Steps)	Wearable (MTI-ActiGraph 7164 (hip))	Spearman correlation; Generalized linear models; Bland-Altman analysis	Between-monitor differences in step counts influence the observed relationship between walking and obesity-related traits.	Some	N/Y
156	Quante et al. (2018, United States)	Adults (N=22; 32.3±11.4 yrs; 59% females; White (N=14); Hispanic (N=3); Other (5))	5 days (1 night)	*ActiGraph GT3X+* (non-dominant wrist; ActiLife 6; 1 minute; Sadeh and Cole–Kripke algorithm); *Actiwatch Spectrum* (non-dominant wrist; Respironics Actiware 5; 30 sec; medium threshold (40 counts per epoch)	Biological state (Total sleep time)	Polysomnography	Intraclass correlation coefficient; Accuracy; Sensitivity; Specificity	The two actigraphs provided comparable and accurate data compared to PSG; although both poorly identified wake episodes (i.e.; had low specificity).	Low	N/N
157	Rabinovich et al. (2013, Greece, Belgium, United Kingdom)	Chronic obstructive pulmonary disease patients (N=80; 68±6 yrs; 23.8% females; NR)	14 days	*ActiGraph GT3X* (right waist; Actilife 5; NR; NR); *DynaPort MoveMonitor* (waist; NR; NR; NR); *Lifecorder PLUS* (left waist; Physical Activity Analysis Software; NR; NR); *Actiwatch Spectrum* (right waist; Respironics Actiware 5; NR; NR); *RT3* (right waist; StayHealthy RT3 Assist v1.0.7; NR; NR); *SenseWear Armband* (left upper arm SenseWear Professional 6.0; NR; NR)	Intensity (Total energy expenditure)	Doubly labelled water	One-way ANOVA; Pearson correlation	GT3X and DynaPort MoveMonitor best explained the majority of the TEE.	Some	N/N

158	Rafamantanantsoa et al. 2002, Japan)	Adults (N=24; 48±10 yrs; 0% females; Japanese (N=24)	14 days	*LifeCorder* (right waist; NR; NR; equation reported)	Intensity (Total energy expenditure)	Doubly labelled water	One-way ANOVA; Pearson correlation; Bland-Altman analysis	These results suggest that; same as the previous study; AC is superior to HR in estimating TEE; and seems to be satisfactory for estimation at both group and individual levels; particularly for large-scale studies of older individuals when compared to the DLW method.	Low	N/NR
159	Redenius et al. (2019, United States)	Adults (N=65; 42±14 yrs; 72.3% females; NR)	7 days	*Fitbit Flex* (non-dominant wrist; Fitbit application program; 1 minute; proprietary algorithm)	Intensity (Time spent in different intensities (sedentary, MVPA))	Wearable (ActiGraph GT3X+ (hip))	Pearson correlation; Spearman correlation; Repeated-measures ANOVA; Bland-Altman analysis; Pitman's Tests; Equivalence tests	In comparison with the GT3X+ accelerometer; the Fitbit Flex provided comparatively accurate estimates of SED; but the Fitbit Flex overestimated MVPA under free-living conditions.	High	N/N
160	Regalia et al. (2020, United States)	Adults (N=54; 66.17±10.07 yrs; 33.9% females; NR)	1 day (1 night)	*E4 wristband* (non-dominant wrist; NR; 30 sec; Sadeh's algorithm, ACTS1's algorithm)	Biological state (Total sleep time)	Polysomnography	Sensitivity; Specificity; Accuracy; Precision; F_1-score; Cohen´s kappa; Bland-Altman analysis; Lin's concordance correlation coefficient	Results provide evidence of promising performance of a fullautomation of the sleep tracking procedure with ACT-S1 on older adults.	Some	N/Y
161	Reid et al. (2017, Canada)	Adults (N=21; 21.23±1.63 yrs; 100% females; NR)	7 days	*Fitbit One* (bra, waist; proprietary online software; NR; proprietary algorithm); *Fitbit Flex* (wrist, proprietary online software; NR; proprietary algorithm)	Intensity (Steps; Time spent in different intensities (sedentary LPA, MVPA))	Wearable (ActiGraph GT3X+ (waist))	Repeated-measures ANOVA; Bland-Altman analysis	Regardless of wear-location all Fitbit devices provide similar activity monitoring and users can wear the devices wherever best accommodates their lifestyle or needs.	High	N/NR
162	Rosenberger et al. (2016, United States)	Adults (N=40; 36 yrs; 53% females; NR)	1 day	*ActiGraph GT3X+* (right wrist, right hip; Actilife 6 NR; Sadeh algorithm); *ActivPAL* (right thigh; ActivPAL3 v7.1.18; NR; NR); *Fitbit One* (left wrist, right hip; iPhone app); *GENEActiv* (right wrist; GENEActive PSSoftware v2.2.; NR; Sadeh algorithm, >644 cpm); *Jawbone UP* (right wrist; iPhone App; NR; NR); *LUMOback* (lower back; iPhone App; NR; NR); *Nike Fuelband*	Intensity (Time spent in different intensities (LPA, MVPA); Steps); Posture/Activity Type (Sedentary time); Biological state (Total sleep time)	Wearable (ActiGraph GT3X+ (right hip); Omron Hj-112 (right hip); ActivPAL (right thigh)); Zmachine	Mean absolute percent errors; Equivalence testing; Bland-Altman analysis	Currently; no device accurately captures activity data across the entire 24-h day; but the future of activity measurement should aim for accurate 24-h measurement as a goal.	High	N/N

				(right wrist; iPhone App; NR; NR)						
163	Rothney et al. (2010, United States)	Adults (N=22; 41.8±13.9 yrs; 68.18% females; NR)	14 days	*ActiGraph GT1M* (hip; Actilife v.4.3.0; 1 sec; two regression model)	Intensity (Energy expenditure)	Doubly labelled water	Paired t-tests; Bland–Altman analysis	The two-regression model with LPF showed good agreement with total EE measured using room calorimeter and DLW. However; the individual variability in assessing time spent in sedentary; low; and moderate PA intensities and related EE remains significant.	Some	N/N
164	Rousset et al. (2014, France)	Adults (N=41; Males: 41.7±6.9 yrs, females: 42.4±8.0 yrs; 48.8% females; NR)	10 days	*Actiheart* (chest; NR; 1 minute; equation reported); *SenseWear Pro-3 Armband* (right upper arm; NR; 1 minute; NR)	Intensity (Total energy expenditure)	Doubly labelled water	Paired t-tests; Bland-Altman analysis; Bias	Both monitors are appropriate for estimating TEE. Armband is more effective than Actiheart at the individual level for daily light-intensity activities.	High	N/NR
165	Rozanski et al. (2018, Canada)	Adults attending in- or outpatient therapy for stroke (N=37; 64.4±15.0 yrs; 35.1% females; NR)	< 1 day (5.5-10 hours)	*Fitbit Charge HR* (wrist; NR; 5 minutes; NR); *Garmin Vivosmart* (wrist; NR; 1 minute; NR)	Intensity (Steps)	Wearable (X6-2mini (ankle))	Spearman correlation; Wilcoxon rank test; Bland-Altman analysis	FBT and GAR had moderate to strong correlation with best available reference measures of walking activity in individuals with subacute stroke.	High	N/N
166	Rutgers et al. (1997, Netherlands)	Adults (N=13; 73±3 yrs; 100% females; NR)	3 days	*Sport Tester PE 400* (chest; NR; 5 minutes; NR)	Intensity (Total energy expenditure)	Self-reported activity questionnaire	Paired t-Test; ANOVA; Pearson correlation	Minute-by-minute HR monitoring did not appear to be a valid method for predicting TEE for individuals or small groups.	High	NR/NR
167	Sánchez-Ortuño et al. (2010, United States)	Adults with and without insomnia (N=62; Insomnia group: 28.4±6 yrs; 61.3% females; Caucasians (N=15); African American (N=10); Asians (N=3); Others (N=3); Normal sleepers: 28.3±4.9 yrs; 58.1% females; Caucasians (N=22); African American (N=5); Asians (N=3); Others (N=1))	3 days (3 Nights)	*Mini-Mitter Actiwatch* (non-dominant wrist; NR; 1 minute; default medium threshold)	Biological state (Total sleep time)	Polysomnography; Self-reported sleep diaries	Pearson correlation; Multiple regression; Repeated measure ANOVA; Linear mixed models	ACT provides informative data for insomnia sufferers and normal sleepers in their usual sleep environments. The ACT estimate of SOL seems sensitive to night-to-night differences in subjective sleep ratings.	High	N/Y
168	Sargent et al. (2016, Australia)	Elite athletes (N=16; 19.3±1.5 yrs; 0% females; NR)	9 days	*Activity monitor* (wrist; Actiware Sleep v3.1; 1 minute; Actiware-Sleep scoring algorithm)	Biological state (Sleep time)	Polysomnography	Bland-Altman analysis; Paired t-tests; Percentage agreement; Sensitivity; Specificity; Cohen´s kappa	Applying the correct sleep–wake threshold is important when using activity monitors to measure the sleep of elite athletes.	Some	N/NR

169	Sasaki et al. (2018, Japan)	Adults (N=29; 34.6±12.5 yrs; 48.28% females; NR)	7 days	*Life Microscope* (waist; NR; 1 minute; NR)	Intensity (Steps; Time spent in different intensities (sedentary, LPA, MVPA))	Wearable (Active Style Pro HJA-750C (left waist))	Pearson correlation; Paired t-tests; Intra-class coefficient; Bland–Altman analysis	Both accelerometers were comparable in their measurement of step counts and time spent in different physical activity intensities under free-living conditions; and either could be used for population studies	High	N/Y
170	Schmal et al. (2018, Denmark)	Elderly patients after operative treatment of proximal femur fractures (N=22; 81±8 yrs; 50% females; NR)	2 days	*Fitbit Flex* (wrist; NR; NR; NR); *Misfit Shine* (ankle, wrist; NR; NR; NR); *Axivity AX3* (thigh; NR; 1 minute; NR)	Intensity (Steps)	Observation (video)	ANOVA; Paired t-test; Mann–Whitney U test; Spearman correlation; Pearson correlation; Sensitivity; Specificity	TheMisfit and the Axivity trackers can reliably monitor activity in elderly patients after operative treatment of proximal femur fractures. However; the wear location is decisive.	Some	N/N
171	Schneider et al. (2004, United States)	Adults (N=20; Males: 39.5±16.6 yrs; females: 43.3±16.6 yrs; 50% females; NR)	1 day	*Accusplit Alliance 1510; Freestyle Pacer Pro; Colorado on the Move; Kenz Lifecorder; New-Lifestyles NL-2000; Omron HJ-105; Oregon Scientific PE316CA; Sportline 330 and 345; Walk4Life LS 2525; Yamax Skeletone EM-180; Yamax Digi-Walker SW-701* (all waist; NR; NR; NR)	Intensity (Steps)	Wearable (Yamax Digi-Walker SW-200 (waist))	Difference score; Two-way repeated-measures ANOVA; Independent t-tests; Bland-Altman analysis	The KZ; YX200; NL; and YX701 appear to be suitable for most research purposes. Given the potential for pedometers in physical activity research; it is necessary that there be consistency across studies in the measurement of "steps per day."	High	N/NR
172	Scott et al. (2019, Australia)	Adults (N=10; 19.3 yrs; 60% females; NR)	7 days	*Fitbit basic model* (wrist; Fitbit application programming interface Fitabase; 1 minute; NR)	Biological state (Total sleep time); Intensity (Time spent in different activities (MVPA, sedentary))	Wearable (Actiwatch-64 (wrist))	Paired t-test; Mean absolute percentage error; Bland-Altman analysis	The findings suggest that it is inappropriate to substitute actiwatches with commercial activity trackers in research setting; as the latter are probably insufficient for precision diagnostics or stratification of cases into treatment relevant subgroups.	High	N/N
173	Semanik et al. (2020, United States)	Adults with chronic knee symptoms (N=35; mean age 52 yrs; 69% females; NR)	7 days	*Fitbit Flex* (wrist; Fitabase; NR; NR)	Intensity (Time spent in different intensities (LPA, MVPA))	Wearable (ActiGraph GT3X+ (waist))	Bland–Altman analysis	The Fitbit Flex does not appear to be an adequate substitute for research-grade accelerometry in this population of persons with chronic knee symptoms.	High	N/N
174	Siddall et al. (2019, United Kingdom)	Soldiers (N=20; 23±2 yrs; 50% females; NR)	10 days	*GENEActiv* (wrist; GENEActiv software v3.1; 1 minute; equation reported); *Fitbit Surge HR* (wrist; NR; NR; NR)	Intensity (Total energy expenditure)	Doubly labelled water	Repeated-measures ANOVA; Bland-Altman analysis; Paired t-tests; Equivalence test; Pearson correlation	Wearable physical activity monitors provide a cheaper and more practical method for estimating free-living TEE than DLW in military settings.	Some	N/NR
175	Silcott et al. (2011,	Adults (N=62; Normal 31.3±8.6 yrs, overweight 35.8±11.0	1 day	*Omron HJ-720ITC pedometers* (waist, pants pocket, neck; NR; 1	Intensity (Steps)	Wearable (StepWatch-3 (ankle))	Percentage accuracy; Two-way repeated-	Results from our study show that the Omron significantly	High	Y/NR

	United States)	yrs, obese 46.2±12.7 yrs; 50% females; NR)		hour; NR); *Yamax SW-200* (waist; NR; NR; NR)			measures ANOVA; Bland-Altman analysis	underestimates steps per day under free-living conditions.		
176	Silva et al. (2015, Portugal)	Adults (N=17; 24.9±4.8 yrs; 0% females; NR)	8 days	*Actiheart* (chest; commercial software v4.0.99; 1 minute; branched equations model)	Intensity (Total energy expenditure; Activity energy expenditure)	Doubly labelled water	Paired sample t-tests; Wilcoxon test; Simple linear regressions; Concordance coefficient correlation; Agreement	Regardless of caffeine intake; the combined HR and motion sensor is valid for estimating free-living energy expenditure in a group of healthy men but is less accurate for an individual assessment.	Some	N/N
177	Silva et al. (2019, United States)	Adults with knee osteoarthritis (N=14; 68.0±8.3 yrs; 64.3% females; NR)	14 days	*Fitbit Charge 2* (wrist; in-house application programming; 1 minute; NR)	Intensity (Time spent in different intensities (sedentary, MVPA))	Wearable (ActiGraph GTX3+ (hip))	Agreement; Sensitivity; Specificity	Using Fitbit for MVPA and sedentary time assessment may lead to inaccurate estimates of both. Fitbit MVPA estimates were generally more conservative than ActiGraph estimates.	High	N/NR
178	Simunek et al. (2016, Czech Republic)	Adults (N=20; 34.0±6.3 yrs; 30% females; NR)	7 days	*Garmin Vivofit* (wrist; NR; NR; NR); *Polar Loop* (wrist; NR; NR; NR)	Intensity (Steps)	Wearable (Yamax Digiwalker SW-701 (waist); ActiGraph GT3X (waist))	Paired t-test; Mean absolute percentage errors; Intraclass correlation coefficient; Bland-Altman analysis	Vívofit showed higher validity than Loop in measuring daily step counts in free-living conditions. Loop appears to overestimate the daily number of steps in individuals who take more steps during a day.	High	N/N
179	Sjöberg et al. (2021, Sweden)	Patients with Chronic Pain (N=42; 43.8±11-8 yrs; 76% females; NR)	3 days	*Fitbit Versa* (wrist; web-based application programming interface; NR; proprietary algorithm)	Intensity (Energy expenditure; Steps)	Wearable (ActiGraph GT3X (waist))	Intraclass correlation coefficient; ANOVA; Spearman correlation; Bland-Altman analysis; Mean absolute percentage error	The wrist-worn device systematically overestimated energy expenditure and showed poor agreement and correlation compared to the criterion standard (Jaeger Oxycon Pro) and the relative criterion standard (ActiGraph GT3X).	High	N/N
180	Skipworth et al. (2011, NR)	Advanced cancer in patients and healthy outpatients (N=14; 64.8±12.5 yrs; 14.3% females; NR)	14 days	*ActivPAL* (right thigh; manufacturer's software; NR; NR)	Intensity (Energy expenditure (MET))	Doubly labelled water	Pearson correlation; Bland-Altman analysis	AM-systems provide valid estimates of body positions and transfers; but not step count; especially in non-self caring patients. ActivPAL can derive estimates of EE but there is considerable variability in results; which is consistent; in part; with the inaccuracy in step count.	Some	N/N
181	Skotte et al. (2014, Denmark)	Adults (N=17; 34±11 yrs; 58.82% females; NR)	< 1 day (9 hours)	*ActiGraph GT3X+* (thigh, hip; Acti4; ActiLife v5.5; NR; algorithm reported)	Posture/Activity Type (sitting)	Wearable (pressure sensor (hip pocket))	Sensitivity; Specificity	The developed method for detecting physical activity types showed a high sensitivity and specificity for sitting; standing; walking; running; walking stairs; and cycling in a standardized setting and for sitting posture during free living.	High	N/NR
182	Slinde et al. (2013, Sweden)	Overweight or obese; lactating women (N=62; 33.2±4.2 yrs; 100% females; NR)	7 days	*SenseWear Armband Pro 2* (upper arm; InnerView Professional 5.1 and 6.1 Software; NR; NR)	Intensity (Total and activity energy expenditure)	Doubly labelled water	Paired t-test; Pearson correlation; Bland-Altman analysis	$TEE_{SWA5.1}$ and $AEE_{SWA5.1}$ were fairly estimated on a group level while $TEE_{SWA6.1}$ and $AEE_{SWA6.1}$ were significantly and	Some	N/N

								systematically underestimated. Both SWA software vs showed		
183	Smits et al. (2018, Australia)	Adults with lower back pain (N=5; 32.2±5 yrs; 40% females; NR)	1 day	*ActivPAL3-micro* (right thigh, trunk; proprietary software activPAL™ v7.2.32; 15 sec; description reported)	Posture/Activity Type (lying)	Wearable (ActivPAL3-micro (right thigh; trunk))	Bland-Altman analysis; Intraclass correlation coefficient; Mean absolute differences	There was some agreement between the single- and dual-monitor estimates of lying time under free-living conditions; but measures were not interchangeable.	High	N/Y
184	St-Laurent et al. (2018, Canada)	Pregnant women (N=16; 32±2 yrs; 100% females; NR)	7 days	*Fibit Zip* (hip; NR; NR; NR); *Fitbit Flex* (wrist; NR; NR; NR)	Intensity (Steps; Time spent in different activities (sedentary, LPA, MVPA))	Wearable (ActiGraph GT3X (hip))	Repeated-measure ANOVA; Bland–Altman analysis; Paired t-test; Linear regression	The validity of Fitbit Zip and Fitbit Flex is good; depending on measured parameters. Fitbit Zip is accurate to measure steps whereas Fitbit Flex is precise for MVPA.	High	N/N
185	Stein et al. (2003, United States)	Habitually active adults (N=28; 30.6±4.70 yrs; 100% females); Sedentary adults (N=28; 27.9±5.35 yrs; 100% females); White (N=45); Native American (N=1); African American (N=2); Hispanic (N=5); Other (N=3))	2 days	*Caltrac motion sensor* (left hip; NR; NR; equation reported)	Intensity (Energy expenditure)	Wearable (Polar Vantage XL (wrist))	Paired sample t-test; Pearson correlation	All methods were sensitive to variation in both the rate of EE and the duration over which activity was monitored. Accelerometry and PAR are useful methods for categorizing EE in epidemiologic studies among pregnant women but absolute estimates are biased relative to HR.	High	N/NR
186	St-Onge et al. (2007, Canada)	Adults (N=45; 35.1±14 yrs; 71.1% females; NR)	10 days	*SenseWear Pro* (upper right arm; Innerview v4.02; NR; NR)	Intensity (Energy expenditure)	Doubly labelled water	Intraclass correlation coefficient; Mean difference; Regression analysis; Bland-Altman analysis; Paired t-test	The portable armband shows reasonable concordance with DLW for measuring daily EE in free-living adults. The armband may therefore be useful to estimate daily EE.	Some	Y/Y
187	Strath et al. (2005, United Kingdom)	Adults (N=10; 25.8±3.4 yrs; 60% females; NR)	< 1 day (6 hours)	*ActiGraph 7164* (wrist, thigh, hip; NR; NR; Freedson equation)	Intensity (Energy expenditure)	Indirect calorimetry	Pearson correaltion; Bland–Altman analysis; Repeated measures ANOVA	The combination of HR and ACC improves the accuracy of PAEE estimates and could be applied in large-scale epidemiological studies.	High	NR/N
188	Sugino et al. (2011, Japan)	Patients with Chronic Obstructive Pulmonary Disease (N=14; 74.3±6.2 yrs; 0% females; NR)	< 1 day (7 hours)	*Actimarker* (waist; NR; 1 minute; NR)	Intensity (Time spent in physical activity)	Wearable (DynaPort Activity Monitor (waist, left leg))	Regression analysis; Wilcoxon signed rank test; Intraclass correlation coefficient; Bland-Altman analysis	The validity of the Actimarker was confirmed; and repeatability was obtained when the data from at least 3 non-rainy weekdays were analyzed.	High	NR/NR
189	Sushames et al. (2016, Australia)	Adults (N=25; 23.7±5.8 yrs; 48% females; NR)	1 day	*Fitbit Flex* (wrist; NR; 1 minute; NR)	Intensity (Steps; Activity Energy expenditure; Time spent in MVPA)	Wearable (ActiGraph GT3X+ (hip))	Paired samples t-tests; Intraclass correlation coefficient; Bland-Altman analysis	The Fitbit Flex has moderate validity for measuring physical activity relative to direct observation and the Actigraph.	High	N/N
190	Svensson et al. (2019, Japan)	Adults (N=20; 25-67 yrs; 50% females; Japanese (N=20))	14 days	*Fitbit Versa* (wrist; Application programming interface;	Biological state (Total sleep time)	Portable EEG system	Sensitivity; Specificity; Accuracy; Bland-Altman	The consumer sleep tracker could be a useful tool for measuring sleep duration in longitudinal	High	N/N

				30 sec; proprietary algorithms)			analysis; Bias; Paired t-tests	epidemiologic naturalistic studies albeit with some limitations in specificity.		
191	Te Lindert et al. (2013, Netherlands)	Adults (N=15; 29.7±3.9 yrs; 33.3% females; NR)	1 day (1 night)	*Actiwatch* (two at non-dominant wrist; Actiwatch Activity and Sleep Analysis software v5.08; 15 sec; equation reported); *GENEActiv* (two at non-dominant wrist; Geneactiv PC software v1.0; 15 sec; NR)	Biological state (Total sleep time)	Self-reported sleep diary	Cohen´s kappa; Bland-Altman analysis	The algorithm allows for continuity of outcome parameters in ongoing actigraphy studies that consider switching to MEMS-accelerometers.	High	N/N
192	Tedesco et al. (2019, Ireland)	Adults (N=20; Males: 70.2±2.9 yrs, females: 71.1±3.1 yrs; 55% females; NR)	1 day	*Fitbit Charge 2* (wrist; NR; NR; NR); *Garmin vivosmart HR+* (wrist; NR; NR; NR)	Intensity (Energy expenditure; Steps; Time spent in MVPA); Biological state (Total sleep time)	Wearables (ActiGraph GT9X-BT (wrist, waist)); New-Lifestyles NL-2000i (waist))	Mean percentage error; Intraclass correlation; Bland-Altman analysis	The tested well-known devices could be adopted to estimate steps; energy expenditure; and sleep duration with an acceptable level of accuracy in the population of interest.	High	N/N
193	Thorup et al. (2017, Denmark)	Patients with cardiac disease (N=24; 67±10.03 yrs; 8.33% females; NR)	1 day	*Fitbit Zip* (waist; NR; 1 minute; NR)	Intensity (Steps)	Wearable (Shimmer 3 (ankle))	Percentage relative error; Interclass correlation coefficient; Bland-Altman analysis	A speed of 3.6 km/hr or higher is required to expect acceptable accuracy in step measurement using a Zip; on a treadmill and in real life. Inaccuracies are directly related to slow speeds; which might be a problem for patients with cardiac disease who walk at a slow pace.	High	N/N
194	Toth et al. (2018, United States)	Adults (N=12; 35±13 yrs; 50% females; NR)	1 day	*StepWatch* (at each ankle two; Modus Health v3.4; 1 minute; NR); *ActivPAL* (at each thigh; AP process and presentation v7.2.28; 1 minute; NR); *Fitbit Zip* (hip; NR; 1 minute; NR); *Yamax Digi-Walker SW-200* (hip; NR; 1 minute; NR); *New Lifestyles NL-2000* (hip; NR; 1 minute; NR); *Fitbit Charge 2* (at each wrist; NR; 1 minute; NR); *ActiGraph GT9X* (at each wrist; ActiLife 6 v6.13.1; 1 minute;	Intensity (Steps)	Observation (video)	Mean absolute percent error; One-sample t-tests; Intraclass correlation coefficient; Pearson correlation	Across all waking hours of 1 d; step counts differ between devices. The SW; regardless of settings; was the most accurate method of counting steps.	High	N/Y

				MAVM step counting algorithm)						
195	Tudor-Locke et al. (2006, United States)	Convenience sample (N=9; 25-40 yrs; 100% females; NR)	1 day	*Kellogg's* Special K* Step Counters* (waist; NR; NR; NR); *Yamax Digiwalker DW-500* (waist; NR; NR; NR)	Intensity (Steps)	Wearable (ActiGraph 7164 (waist))	Absolute percent error; Intraclass correlation coefficient	K pedometers are unacceptably inaccurate.	High	N/NR
196	Tully et al. (2014, Northern Ireland)	Adults (N=42; median age 42 yrs; 59.5% females; NR)	7 days	*Fitbit Zip* (waist; NR; NR; NR)	Intensity (Steps)	Wearable (ActiGraph GT3X (waist); Yamax CW700 (waist))	Spearman correlation; Wilcoxon signed rank tests; Bland-Altman analysis	Given the high level of correlation and no apparent systematic biases in the Bland Altman plots; the use of Fitbit Zip as a measure of physical activity. However, the Fitbit Zip recorded a significantly higher number of steps per day than the Actigraph.	High	N/N
197	Uiterwaal et al. (1998, Netherlands)	Maintenance mechanic in a city hall (N=1; 42 yrs; 0% female; NR)	2 days	*DynaPort ADL3 Monitor* (waist; NR; NR; NR)	Posture/Activity Type (lying, swing/seesaw, locomotion; standing; sitting)	Observation (video)	Sensitivity; Predictive value	Considering the simple instrumentation of the ADL monitor this validity is considered good.	High	NR/NR
198	Vähä-Ypyä et al. (2017, Finland)	Adults (N=13; 26-62 yrs; NR; NR)	20 days	*Hookie AM20* (hip; NR; 6 sec; equations reported)	Posture/Activity Type (sitting, standing, walking, running, cycling)	Wearable (Hookie AM20 (thigh))	Sensitivity; Specifictiy; Accuracy; Cohen´s kappa	The proposed APE analysis of the raw data from hip-worn triaxial accelerometer gives accurate and specific information about daily times spent lying, sitting, and standing.	High	N/N
199	Valenti et al. (2014, Netherlands)	Overweights and obese adults (N=36; 41±7 yrs; 69% females; NR)	14 days	*Tracmor$_D$* (lower back; Direct life; 1 minute; NR)	Intensity (Counts; Activity energy expenditure)	Doubly labelled water	Pearson correlation; Squared correlation coefficient	Equations derived with the Tracmor$_D$ allow valid assessment of PAL and AEE.	Some	N/Y
200	Van Alphen et al. (2020, Netherlands)	Adults with intellectual and visual disabilities (N=22; 35.1±13.6 yrs; 50% femlaes; NR)	< 1 day (2 hours)	*Actiwatch-2* (wrist; Philips Actiware 6.0.9; 15 sec; NR)	Postures/Activity Type (lying, sitting, standing still, standing/moving)	Observation (video)	Multilevel analysis	The Actiwatch-2 seems able to assess obvious upper body movement in people with PIMD; and whether there is involvement in an activity situation.	High	N/N
201	Van Blarigan et al. (2017, United States)	Prostate cancer survivors (N=25; Median 66 yrs; 0% female; White (N=15); Asian (N=1); Other (N=6)	7 days	*Fitbit One* (right hip; manufacturer´s website; 1 minute; 3-5.9 METs (faily active), ≥ 6 METs (vigorous)); *Omron pedometer HJ-322U* (right hip; manufacturer´s website; NR; NR)	Intensity (Energy expenditure (MET))	Wearable (ActiGraph GT3X+ (right hip)); Self-reported activity diary	Pearson correlation; Spearman correlation	Among prostate cancer survivors, the Fitbit One's activity and step measurements were well correlated with the ActiGraph GT3X+ and Omron pedometer.	High	Y/N

202	Van de Wouw et al. (2013, Netherlands)	Older adults with intellectual disability (ID) (N=7; 65.3±8.75 yrs; 14% females; NR)	2 days (2 nights)	*Actiwatch-7* (wrist; Actiwatch Sleep Analysis 7 software; 1 minute; NR); *Actiwatch-2* (wrist; Actiware software; 1 minute; NR)	Biological state (Total sleep time)	Polysomnography	Accuracy; Bland-Altman analysis	We recommend using the high sensitivity setting of the Actiwatch for clinical evaluation of sleep, and for epidemiological research in older adults with ID.	High	N/N
203	Van den Berg-Emons et al. (2000, Netherlands)	Patients with congestive heart failure (N=10; Median 63 yrs; 10% females; NR)	< 1 day (45 min)	*Vitaport 2* (each leg, thigh, two at chest; S.P.I.L; NR; NR)	Posture/Activity Type (lying, standing, sitting, walking, cycling)	Observation (video)	Agreement; Sensitivity; Predictive value	The Activity Monitor is a valid instrument to quantify several aspects of everyday physical activity in congestive heart failure.	High	N/NR
204	Van der Weegen et al. (2015, Netherlands)	Healthy (N=10; 30.4±8.3 yrs; NR; NR); Chronically ill adults (N=12; 61.6±9.2 yrs; NR; NR)	6-7 days	*MMOXX 1.01* (lower back; MOXBWO; 1 minute; NR)	Intensity (Counts)	Wearable (ActiGraph GT3X (lower back))	Spearman correlation; Bland-Altman analysis	The MOX is capable of measuring physical activity and can be used in the It's LiFe! intervention.	High	N/N
205	Van Hees et al. (2011, Sweden)	Women (N=108; 20-35 yrs; 100% female; NR)	10 days	*GENEActiv* (wrist; NR; NR; equation reported)	Intensity (Activity energy expenditure)	Doubly labelled water	Linear regression analysis; Bland-Altman analysis	A simple summary measure derived from a wrist-worn tri-axial accelerometer adds significantly to the prediction of energy expenditure	Low	Y/N
206	Van Hees et al. (2018, United Kingdom)	Adults (N=3752; 69.1±5.6 yrs; 25% females; NR)	9 days (9 nights)	*GENEActiv* (non-dominant wrist; NR; 5 sec; equation reported)	Biological state (Sleep period time window)	Self-reported sleep diary	Multi-level regression analysis; Correlation coefficient; Mean absolute error	We demonstrated the accuracy of our algorithm to detect the SPT-window.	High	N/N
207	Van Nassau et al. (2015, Australia)	Staff from a non-government health agency (N=42; 38±11 yrs; 86% females; NR)	11 weeks	*ActiGraph GT1M and GT3X* (right hip; NR; NR; < 100 cpm)	Posture/Activity Type (sitting, standing)	Wearable (ActivPAL (thigh))	Spearman correlation; Bland-Altman analysis	This study suggests that studies aimed at determining differences in occupational sitting and standing time should use activPAL-type inclinometers as a preferred type of objective measure.	High	N/N
208	Vanhelst et al. (2012, France)	Sport science students (N=25; Males 25.3±4.8 yrs, females 25.5±4.4 yrs; 44% females; NR)	1 day	*ActiGraph GT3X* (level of the back; NR; 1 minute; NR)	Intensity (Counts)	Wearable (ActiGraph GT1M (level of the back))	ANOVA; Intraclass correlation coefficient; Concordance correlation coefficient; Bland-Altman analysis	Findings suggest that the two accelerometers provided similar results and therefore the GT3X may be used in clinical and epidemiological studies without additional calibration or validation studies.	High	NR/N
209	Varela Mato et al. (2017, United Kingdom)	Bus drivers (N=28; 43.9±27 yrs; 0% females; NR)	7 days	*ActiGraph GT3X+* (waist; ActiLife software v6.11.8; 1 minute; < 50, <100, <150, <200, <250, <300 cpm)	Intensity (Time spent sedentary)	Wearable (ActivPAL (thigh))	Paired sample t-tests; Cohen´s d; Intraclass correlation coefficient; Bland-Altman analysis	The use of the ActiGraph to measure sedentary time in this understudied, highly sedentary and at risk occupational group is not recommended.	High	N/NR
210	Vetrovsky et al. (2019, Czech Republic)	Patients with heart failure (N=15; 65.5±12.6 yrs; 40% females; NR); Healthy adults (N=14;	3 days	*Withings GO* (non-dominant wrist; NR; NR; NR); *Fitbit Charge 2* (dominant wrist; NR; NR; NR); *Gramin vivofit* (dominant wrist; NR;	Intensity (Steps)	Wearable (Actigraph wGT3X-BT (waist))	Concordance correlation coefficient; Mean percentage error; Mean absolute percentage error	Even though none of the tested activity monitors fall within arbitrary thresholds for validity, most of them perform reasonably well enough to be useful tools that clinicians can use to simply	High	N/N

		43.3±18.9 yrs; 64% females; NR)		NR; NR); *Garmin vivofit 3* (dominant wrist; NR; NR; NR); *Omron HJ-322U-E* (waist; NR; NR; NR); *SmartLAB walk +* (neck; NR; NR; NR)				motivate chronic heart failure patients to walk more.		
211	Villars et al. (2012, France)	Healthy male volunteers (N=35; 27.6±6.5 yrs; 0% females; NR)	7 days	*Actiheart* (chest; Actiheart software; 1 minute; branched equations model); *RT3* accelerometer (waist; NR; 1 minute; proprietary algorithm)	Intensity (Activity energy expenditure)	Doubly labelled water	Agreement; Pearson correlation; Root mean sqaure error; Intraclass correlation coefficient	This study supports a good level of agreement between the Actiheart ACC/HR estimates and DLW-measured AEE in lean and overweight men with varying fitness levels.	Low	N/N
212	Washburn et al. (1990, United States)	Adults (N=45; 72.9±5.3 yrs; 49% females; NR)	3 days	*Caltrac* (non-dominant hip; NR; NR; NR)	Posture/Activity Type (sitting, lying, standing, walking)	Self-reported activity diary	Spearman correlation	The association of the CaItrac and a self-reported diary assessment of physical activity shown in our data and the observation of a lower mean daily Caltrac reading in our older; compared with our younger; population provides evidence for the validity of the CaItrae for the assessment of daily physical activity in healthy older individuals.	High	N/NR
213	Webber & St. John, 2016, United States)	Older adults with a variety of diagnoses (N=38; 83.2±7.1 yrs; 89% females; NR)	1 day	*ActiGraph GT3X+* (right hip, ankle; Actilife 6; 1 sec; LFE algorithm)	Intensity (Steps)	Wearable (Step Watch 3.0 (ankle))	One-way ANOVA; Intraclass correlation coefficient; Bland-Altman analysis	Although these finding suggest the GT3X+ (ankle, LFE) functions as well as the StepWatch in detecting steps during walking in older adults with slow gait speeds, further research is needed to determine whether the GT3X+ is also able to disregard other body movements (e.g., fidgeting) that occur when full day monitoring is utilized.	High	N/NR
214	Welk et al. (2007, United States)	Adults (N=30; 24.9±6.1 yrs; 56.67% females; NR)	< 1 day (approx. 5.5 hours)	*Sense Wear Pro 2* (arm; Interview Research Software v3.9; 1 minute; proprietary alforithm); *MTI Actigraph* (hip; NR; 1 minute; six different estimates)	Intensity (Energy expenditure; Time spent in MVPA)	Wearable (IDEEA (both feet; both thighs; chest))	Univariate t-tests; Pairwise correlation; Bland-Altman analysis	The study indicates that the Matthews MTI cut-off point and the new SP2 equation provide the most accurate indicators of PA.	High	NR/NR
215	Welk at al., 2017, United States)	Adults (N=52; 28.1±10.8 yrs; 46% females; NR)	7 days	*Metria IH1* (left arm; NR; 1 minute; proprietary algorithms)	Intensity (Energy expenditure)	Wearable (Sensewear core monitor (left arm))	Pearson correlation; Mean absolute percent error	The disposable nature of the adhesive Metria IH1 monitor offers promise for clinical evaluation of physical activity behaviour in patients.	High	Y/N
216	White et at. (2019,	Adults (N=193; 40-66 yrs; 46% females; NR)	8 days	*AX3* (each wrist, right thigh; pampro v0.4.0; 5 sec; equations reported)	Intensity (Total energy expenditure)	Doubly labelled water	Pairwise mean bias and 95% limits of agreement; Root Mean	Acceleration measured at either wrist or thigh can be used to estimate population levels of AEE	Some	N/Y

	United Kingdom)						Squared Error; Pearson correlation; linear regression	and TEE in free-living conditions with high precision.		
217	Whybrow et al. (2013, United Kingdom)	Adults (N=15; 38.9±10.4 yrs; 53% females; NR)	12 days	*IDEEA* (eight sensors; IDEEA's software; NR; NR)	Intensity (Energy expenditure)	Doubly labelled water	Student's t tests; Pearson correlation coefficient; Lin's concordance coefficient; Bland–Altman analysis	The IDEEA system overestimated EE in the free-living environments.	High	N/N
218	Williams et al. (2020, United States)	Young adults with insomnia (N=34; 19.97±2.11 yrs; 56% females; 59% Caucasian, 21% Hispanic, 12% African American, 6% Asian/Pacific Islander, 3% Multiracial)	8 days (1 night)	*Actiwatch AW64* (non-dominant wrist; Actiware v5.5; 30 sec; NR)	Biological state (Total sleep time)	Polysomnography	Bivariate correlation; Intraclass correlation coefficient; Paired samples t-tests; One-way repeated measures ANOVA; Bland-Altman analysis	Actigraphy was a valid objective measure of SOL, WASO, TST, and SE in a young adult insomnia sample, as compared to ambulatory PSG.	High	Y/N
219	Winkler et al. (2016, Australia)	Adults (N=741; median age 57 yrs; 56% females; NR)	7 days (7 nights)	*ActivPAL 3* (thigh; activPAL software 6.4.1; NR; algorithm reported)	Biological state (Wake time)	Self-reported sleep diary	Kappa statistics; Bland-Altman analysis	In free-living data from Australian adults; a simple algorithm developed in a different population showed 'almost perfect' agreement with the diary method for most individuals (88%).	High	N/NR
220	Wu et al. (2021, Canada)	Normotensive adults (N=32; 25±6 yrs; 60% females; NR)	7 days	*ActivPAL* (each thigh; PAL Software Suite v7; NR; algorithm reported)	Intensity (Energy expenditure (MET); Steps)	Wearable (PiezoRxD (hip))	Friedman's ANOVA; Bland-Altman analysis; Absolute mean error; Equivalence testing	We present a simple; data processing technique that uses an alternative curvilinear cadence-MET equation that improves the ability of the activPAL to measure intensity-related physical activity in both laboratory and free-living settings.	High	N/N
221	Yamada et al. (2018, Japan)	Community-dwelling healthy older adults (N=44; 64-96 yrs; 55% females; NR)	14 days	*Kenz Lifecorder EX* (NR; NR; NR; NR)	Intensity (Total energy expenditure)	Doubly labelled water	Correlation coefficient; Bland-Altman analysis	ACCTRI and sPAR underestimated TEE and PAL, and adequate corrections are required. The corrected ACCTRI and sPAR are both useful tools to estimate TEE and PAL.	Low	N/NR
222	Yoshida et al. (2019, Japan)	Endurance runners (N=8; 20±1 yrs; 100% females; NR)	7-8 days	*Omron Active style Pro HJA-350IT* (right hip; NR; 1 minute; equation reported)	Intensity (Total energy expenditure)	Doubly labelled water	Paired t-tests; Pearson correlation; Bland-Altman analysis	Findings suggest that the combined method has high validity against the DLW method.	Some	NR/N

[1]N= No; [2]Y=Yes; [3]NR=Not reported

Table S5. Validity of wearables separated by dimensions and age groups.

	Wearable	Younger aged adults (18-40 yrs)			Middle aged adults (41-64 yrs)			Older aged adults (≥ 65 yrs)		
		Bio.[1]	Pos./AT[2]	Int.[3]	Bio.[1]	Pos./AT[2]	Int.[3]	Bio.[1]	Pos./AT[2]	Int.[3]
Uniaxial accelerometer	Actical			↓; ↓			↔			
	ActiGraph AM7164			↑; ↑; ↔; ↔; ↔						
	ActiGraph GT1M		↓	↑; ↓; ↓			↔			↔; ↑
	Activity Monitor IC-3031					↑				
	Actiwatch activity monitor	↓; ↔; ↑								
	Actiwatch Spectrum	↔; ↑; ↔						↔		↔
	Actiwatch 64	↑		↓						
	Caltrac accelerometer			↔					↔	↓; ↑
	CSA accelerometer			↔						
	Lifecorder				↔					
	Lifecorder PLUS									↔
	Motionlogger Sleepwatch	↔								
	MSR2005	↑								
	MyWellness Key						↑; ↔			
	New Lifestyle NL-1000			↑						
	Polar Active Watch			↔; ↓						
	Polar activity recorder FA20			↑						
	Polar RS800CX			↔						
	Sleepwatch-O							↔; ↔		
Biaxial acc.	Actiwatch-2	↔	↔					↔		
	ADXL202					↑				
	Omron HJ-203			↔; ↑						
	Omron Walking Style Pro HJ-720IT			↓; ↓; ↔; ↑; ↓			↓			↓; ↔
	SenseWear Pro	↔		↔			↔			↔; ↔
	SenseWear Pro 2			↔; ↔						
	SenseWear Pro 3			↓; ↔; ↓; ↓			↑			↔; ↑
	Stepwatch Activity Monitor			↔; ↑						
Triaxial acc.	3dNX model v2			↑						
	Actical Z series	↔								
	ActiGraph GT3X		↓; ↔; ↓	↔; ↑; ↓; ↓; ↑; ↔; ↑; ↔; ↓; ↑		↑; ↓; ↔	↓; ↔; ↔			↔; ↑
	ActiGraph GT3X+	↔; ↔; ↑	↓; ↔; ↔; ↑	↓; ↔	↑	↓; ↔; ↔; ↓	↔; ↑; ↓		↔	↓; ↔
	ActiGraph GT9X	↑; ↓		↓; ↓						
	Actiheart			↑; ↑; ↔; ↔; ↑		↓	↔			
	Actillume recorder				↑			↑		
	Actimaker EW4800P			↓						↑
	ActivPAL		↑; ↑; ↔	↓; ↓; ↔; ↑; ↓; ↓; ↔		↑; ↑	↔			
	ActivPAL3				↑				↑	↔
	ActivPAL3-micro		↑							
	Actiwatch AW 7							↔		
	Apple Watch			↑; ↓						
	Apple Watch series 2			↔						
	Axivity AX3		↑	↑			↔			↑
	Basis B1 Band			↓						
	Basis Peak	↓								
	CAM		↑			↑				
	Dynaport ADL Monitor		↑							
	DynaPort ADL3 Monitor					↑				
	Dynaport MoveMonitor								↔	↑; ↑
	Dynaport MoveMonitor MicroMod								↔	
	Dynastream AMP-331			↔						
	E4 wristband							↔		
	Epson PULSENSE			↓						
	Fitbit Alta	↔; ↑		↔; ↔	↑					↓
	Fitbit basic model	↓		↔						
	Fitbit Charge HR	↑; ↔; ↔		↔; ↑; ↓; ↓			↔			↔
	Fitbit Charge	↔; ↔		↔; ↔			↓			
	Fitbit Charge 2	↔; ↔		↔; ↔; ↓; ↔			↓; ↓; ↔	↓		↓; ↓; ↔; ↓
	Fitbit Charge 3		↔							
	Fitbit Flex	↔; ↔; ↑; ↔		↓; ↔; ↔; ↔; ↓; ↔; ↓; ↔; ↑; ↔; ↔; ↔			↔; ↔; ↓			↔; ↓
	Fitbit Flex 2						↔			

	Fitbit One	↑; ↔	↔	↔; ↔; ↔; ↔; ↓			↔			↑; ↔
	Fitbit Surge		↔	↔; ↓						
	Fitbit Versa	↔			↔		↔			
	Fitbit Zip	↑		↑; ↔; ↔; ↑; ↔; ↓			↔			↓; ↑; ↔
	Garmin Vivofit 1	↑		↓; ↓; ↓; ↔						↔
	Garmin Vivofit 2			↓			↔			
	Garmin Vivofit 3			↔			↑			↔
	Garmin Vivosmart	↔						↓		↔
	Garmin Vivosmart 4				↔					
	GENEActiv	↔; ↓; ↑	↔; ↓	↓; ↑; ↔				↑		
	Hookie AM20		↑			↑				
	Huawei Watch			↔						
	Jawbone UP	↔; ↑; ↔; ↔		↓; ↔; ↔; ↔; ↑; ↓						
	Jawbone UP3	↔; ↔								
	Jawbone UP24			↓; ↓; ↔; ↓						
	Kenz Lifecorder EX			↔; ↓; ↑; ↑						↔; ↔
	Kenz e-style2			↑						
	kmsMove-sensor						↑			
	Kronwise 3.0				↑					
	Life Microscope			↑						
	Lifesource XL-18						↔			
	Lumoback		↑	↑; ↓						
	Metria IH1			↑						
	Microsoft Band			↓						
	Mio FUSE			↓						
	Misfit Shine	↑; ↔; ↔		↓; ↓; ↔; ↔; ↑; ↓						↑; ↔
	Misfit Shine 2	↔								
	MMOXX 1.01			↔			↔			
	Motionlogger Microwatch Actigraph	↔								
	MotionSense HRV			↑						
	MotionWatch 8	↔								↑
	Movband Model 2			↑						
	MSR2010	↑								
	MyKronoz ZeFit4	↔		↔						
	New Lifestyles NL-2000			↓; ↔						
	Nike + FuelBand SE			↓; ↔; ↔; ↔; ↓						
	Nokia GO	↓; ↔		↔; ↓						
	Omron Active Style Pro HJA-350IT			↑		↓				
	Omron Active Style Pro HJA-750C			↔						↑
	Omron CaloriScan HJ-306			↑						
	Polar A300									↑
	Polar A360	↔								
	Polar Loop	↔		↓; ↑; ↓; ↓						
	Polar M430						↔			
	Polar M600			↔						
	Polar V800			↔						
	RT3			↔; ↔						↔
	Runscribe inertial sensors		↑							
	Samsung Gear Fit2	↑								
	SENS Motion System								↔	
	SenseWear Mini	↓; ↔		↓; ↔						↔
	SitFIT					↑	↑			
	SmartLAB walk +						↑			↔
	Stepwatch 3 Activity Monitor			↑						
	Tanita AM-160			↓						
	Tanita Calorism Smart			↑						
	Tracmor$_D$						↑; ↑; ↔			
	Tritrac-R3D			↔						
	Verisense	↔		↔						
	VeryFit 2.0	↔		↔						
	VitaBit		↔							
	WHOOP Strap 2.0	↑								
	Withings Activite									↔
	Withings GO									↔
	Withings Pulse			↑; ↔; ↑						
	Withings Pulse O2	↔; ↔		↔; ↓						
	Xiaomi Mi Band	↔								
	Xiaomi Mi Band 2	↓; ↔		↔; ↑						

**Pedometer**	Accusplit Alliance AL300						↑			
	Accusplit Alliance 1510			↓						
	Accusplit-AX120			↔						
	Brookstone Talking						↔			
	Colorado on the Move			↔						
	Freestyle Pacer Pro			↓						
	Geonaute	↔		↔						
	Kellogg's* Special K* Step Counters			↓						
	Oregon Scientific PE829						↓			
	Oregon Scientific PE316CA			↓						
	PiezoRx			↑			↑			
	Silva pedometer model 56012			↓						
	Sportline 330			↓; ↑						
	Sportline 343						↓			
	Sportline 345			↔						
	Striiv Smart Pedometer			↔						
	Walk4Life LS 2525			↓						
	Yamax Digiwalker CW-700						↔			
	Yamax Digiwalker DW-500			↔						
	Yamax Digiwalker SW-200			↓; ↓; ↔; ↑; ↑; ↔; ↔			↔			
	Yamax Digiwalker SW-701			↓; ↑						
	Yamax Skeletone EM-180			↔						
**Unclear**	Activity monitor	↔								
	Aquarius			↓						
	Asus Zenwatch 3			↔						
	HealthPatch		↔							
	iHealth Edge			↔						
	Intelligent Device for Energy Expenditure and Activity			↓; ↓			↑	↑		
	iWown i5 Plus	↔		↔						
	Omron CaloriScan HJA-401F			↔						
	Omron HJ-105			↔; ↓						
	Omron HJ-322U						↑			↔; ↔
	Polar Activity Watch 200			↑			↑			
	Polar Sports Tester 1000 HR			↔						
	Sport Tester PE 400									↓
	Vitaport 2					↔				

Notes:
[1] Biological State; [2] Posture/Activity Type; [3] Intensity
↑: Moderate to strong validity; ↔: Mixed validity; ↓: Poor/weak validity

Table S6. Overview of wearables used in validation studies.

Model (Manufacturer)	Type	Memory/ Battery life	Feedback display (yes/no)	Epoch-length	Dynamic range [g]	Sampling frequency [Hz]	Dimensions (W x D x H) [mm]	Weight [g]	Used in No. Studies
**3dNX accelerometer model v2** (BioTel Ltd., Bristol, UK	Research-grade triaxial accelerometer	NR[1]	No	NR	NR	100	125 x 58 x 8	93	1
**Accusplit Alliance AL300** (Accusplit Inc., Livermore, CA, USA)	Commercial-grade pedometer	NR	Yes	NR	NR	NR	NR	136	1
**Accusplit Alliance 1510** (Accusplit Inc., Livermore, CA, USA)	Commercial-grade pedometer	NR	Yes	NR	NR	NR	NR	NR	1
**Accusplit-AX120** (Accusplit Inc., Livermore, CA, USA)	Commercial-grade pedometer	NR	Yes	NR	NR	NR	NR	90.8	1
**Actical accelerometer** (Philips Respironics, Inc., Murrysville PA, USA)	Research-grade uniaxial accelerometer	NR	No	1-5 sec	NR	32	28 x 27 x 10	17	3
**Actical Z series** (Mini-Mitter Philips Respironics, Inc., Bend, OR, USA)	Research-grade triaxial accelerometer	NR	No	1- 60 sec	0.05-2	32	NR	NR	1
**ActiGraph AM7164** (MTI Health Services, Florida, USA)	Research-grade uniaxial accelerometer	32 MB/4 days	No	10 sec	0.05-2.13	10	51 x 41 x 15	37.8	5
**ActiGraph GT1M** (ActiGraph, LLC, Pensacola, FL, USA)	Research-grade uniaxial accelerometer	1 MB/14 days	No	5 sec	0.05-2	30	38 x 37 x 18	27	7
**ActiGraph GT3X** (ActiGraph, LLC, Pensacola, FL, USA)	Research-grade triaxial accelerometer	2 GB/31 days	No	1-60 sec	0.05-2	30 – 100	38 x 37 x 18	27	19
**ActiGraph GT3X+** (ActiGraph, LLC, Pensacola, FL, USA)	Research-grade triaxial accelerometer	2 GB/31 days	No	1-60 sec	± 6	30 – 100	46 x 33 x 15	19	17
**ActiGraph GT9X** (ActiGraph Inc, Pensacola, FL, USA)	Research-grade triaxial accelerometer	4 GB/16 days	Yes	1-60 sec	± 8	30 - 100	35 x 35 x 10	14	4
**Actillume recorder** (Ambulatory Monitoring, Inc., Ardsley, NY)	Research-grade triaxial accelerometer	32 K RAM/ 7 days	NR	60 sec	0.003	20	10 x 30 x 60	85	2
**Actiheart** (CamNtech, Cambridge, UK)	Research-grade triaxial accelerometer	1 GB/21 days	No	15, 30, 60 sec	± 2.5	100	39.7 x 30.2 x 9.25	10.5	7
**Actimarker EW4800P** (Panasonic Electric Works Co., Ltd, Osaka, Japan)	Research-grade triaxial accelerometer	180 days	No	60 sec	0-2	20	60 x 35 x 13	24	2
**ActivPAL** (PAL Technologies Limited, Glasgow, UK)	Research-grade triaxial accelerometer	16 MB/10 days	No	15 sec	± 2	20	50 x 35 x 7	15	12
**ActivPAL3** (PAL Technologies Limited, Glasgow, UK)	Research-grade triaxial accelerometer	16 MB/7 days	No	1 sec	± 2	20 / 80	53 × 35 × 7	15	3
**ActivPAL3-micro** (PAL Technologies Ltd, Glasgow, Scotland**)**	Research-grade triaxial accelerometer	7 days	No	15 sec	± 2	20	23.5 x 43 x 5	9.5	1
**Activity monitor** (Philips Respironics, Bend, USA)	Research-grade accelerometer	NR	NR	60 sec	0.01	32	NR	NR	1
**Activity Monitor IC-3031** (Temec Instruments BV, Kerkrade, Netherlands)	Research-grade uniaxial accelerometer	NR	No	NR	NR	16	15 x 20 x 5	NR	1
**Polar Activity Watch 200** (Polar Electro Oy, Kempele, Finland)	Commercial-grade device	<100 hours/ 2 years	Yes	NR	NR	NR	NR	83	1

**Actiwatch activity monitor** (Mini Mitter Company, Inc., Sunriver, OR, USA)	Research-grade uniaxial accelerometer	NR	NR	NR	NR	32	27 x 26 x 9	NR	3
**Actiwatch Spectrum** (Philips Healthcare, Andover, MA, USA)	Research-grade uniaxial accelerometer	1 MB/8 months	Yes	NR	0.5-2	32	48 x 37 x 14	30	5
**Actiwatch-2** (Philips Respironics Inc., Murrysville, PA, USA)	Research-grade biaxial accelerometer	1 MB/30 days (at 1 min epoch)	No	NR	0.5-2	32	43 x 23 x 10	16	3
**Actiwatch AW 7** (Cambridge Neuro-technology Ltd., Cambridge, U.K.)	Research-grade triaxial accelerometer	180 days/ 8 years	Yes	1 min	≥ 0.05	32	39 x 32 x 9	10.2	1
**Actiwatch 64** (Mini-Mitter, Inc., Bend, Ore, USA)	Research-grade uniaxial accelerometer	64 KB/180 days	Yes	15 sec - 15 min	NR	32	29 x 37 x 12	16	2
**ADXL202** (Analog Devices BV Ltd, Limerick, Ireland)	Research-grade biaxial accelerometer	NR	NR	NR	± 2	0.01-5000	5 x 5 x 2	5	1
**Apple Watch** (Apple Inc., Los Altos, CA; USA)	Commercial-grade triaxial accelerometer	NR	Yes	NR	NR	NR	38.6 x 33.3 x 10.5	25	2
**Apple Watch series 2** (Apple Inc., Los Altos, CA; USA)	Commercial-grade triaxial accelerometer	<18 Std.	Yes	NR	NR	NR	38.6 x 33.3 x 11.4	28.2	1
**Aquarius** (Aquarius Accessories Ltd, UK).	Commercial-grade device	NR	NR	NR	NR	NR	NR	NR	1
**Asus Zenwatch 3** (Asus Corp., Taipeh, Taiwan)	Commercial-grade device	4 GB/ <61 Std.	Yes	NR	NR	NR	45 x 45 x 10	60	1
**Axivity AX3** (Axivity Ltd., York, UK)	Research-grade triaxial accelerometer	512 MB/30 days (with 12.5 Hz)	No	NR	± 16	12.5 - 3200	23 x 32.5 x 8.9	11	4
**Basis B1 Band** (Basis Science, Inc., San Francisco, CA, USA)	Commercial-grade triaxial accelerometer	4 days	Yes	30 sec	NR	NR	36 x 273 x 27	44	1
**Basis Peak** (Intel, Santa Clara, CA, USA)	Commercial-grade triaxial accelerometer	4 days	Yes	NR	NR	NR	36 x 273 x 27	24	1
**Brookstone Talking Pedometer** (Brookstone Co Inc., Peterborough, NH, USA)	Commercial-grade pedometer	NR	Yes	NR	NR	NR	NR	99	1
**Caltrac accelerometer** (Muscle Dynamics Fitness network, Torrance, CA, USA)	Research-grade uniaxial accelerometer	NR	No	NR	NR	NR	70 x 70 x 20	78	4
**CAM** (Maastricht Instruments BV, Maastricht, Netherlands)	Research-grade triaxial accelerometer	2 GB/ NR	NR	1 sec	±4	25	63 x 45 x 18	100	2
**Colorado on the Move** (NR)	Pedometer	NR	NR	NR	NR	NR	NR	NR	1
**CSA accelerometer** (Computer Science and Applications, Inc., Shalimar, FL, USA)	Research-grade uniaxial accelerometer	NR	No	NR	0.05-3.2	0.25-2.5	66 x 43 x 15	70	1
**Dynastream AMP-331** (Dynastream Innovations, Inc., Alberta, Canada)	Research-grade triaxial accelerometer	7 days	Yes	NR	NR	NR	NR	NR	1
**Dynaport ADL Monitor** (McRoberts, Den Haag, Netherlands)	Research-grade triaxial accelerometer	10 MB/1 day	No	1-60 sec	14	32	125 x 95 x 34	295	3
**DynaPort ADL3 Monitor** (McRoberts BV, Den Haag, Netherlands)	Research-grade triaxial accelerometer	NR	NR	NR	NR	NR	NR	NR	1
**Dynaport MoveMonitor** (Mc Roberts BV, Den Haag, Netherlands)	Research-grade triaxial accelerometer	7 days	NR	NR	NR	NR	84 x 50 x 8	70	1
**Dynaport MoveMonitor MicroMod** (Mc Roberts BV, Den Haag, Netherlands)	Research-grade triaxial accelerometer	64 MB/ 72 hours	NR	NR	NR	100	83 x 51 x 8	40	1

**E4 wristband** (Empatica, Milan, Italy)	Research-grade triaxial accelerometer	< 60 hours/ > 32 hours	NR	NR	± 2	32	44 x 40 x 16	25	1
**Epson PULSENSE** (Seiko Epson, Suwa, Japan)	Commercial-grade triaxial accelerometer	NR/ 36 hours	NR	NR	NR	NR	NR	NR	1
**Fitbit Alta** (Fitbit Inc., San Francisco, CA, USA)	Commercial-grade triaxial accelerometer	< 30 days/ 5 days	Yes	NR	NR	NR	NR	32	5
**Fitbit Charge HR** (Fitbit Inc, San Francisco, CA, USA)	Research-grade triaxial accelerometer	30 days/5 days	Yes	NR	NR	NR	157.5-193-21	30	8
**Fitbit Charge (**Fitbit Inc., San Francisco, CA, USA)	Commercial-grade triaxial accelerometer	<30 days/ 5-7 days	Yes	NR	NR	NR	NR	NR	4
**Fitbit Charge 2 (**Fitbit Inc., San Francisco, CA, USA)	Commercial-grade triaxial accelerometer	< 30 days/ 5 days	Yes	30 sec	NR	NR	103 x 5 x 225	150	11
**Fitbit Charge 3 (**Fitbit Inc., San Francisco, CA, USA)	Commercial-grade triaxial accelerometer	< 30 days/ 7 days	Yes	1 sec, 5 sec	NR	NR	19.9 x 34.5 x 22.7	32	1
**Fitbit Flex** (Fitbit Inc, San Francisco, CA, USA)	Commercial-grade triaxial accelerometer	5 days/7 days	No	NR	NR	NR	32 × 12 × 10	14.6	20
**Fitbit Flex 2 (**Fitbit Inc., San Francisco, CA, USA)	Commercial-grade triaxial accelerometer	< 30 days/ 5 days	No	NR	NR	100	31 x 9 x 7	23.5	1
**Fitbit One (**Fitbit Inc., San Francisco, CA, USA)	Commercial-grade triaxial accelerometer	< 23 days/ 10 days	Yes	60 sec	NR	NR	19.3 x 48 x 9.65	8	9
**Fitbit Surge (**Fitbit Inc., San Francisco, CA, USA)	commercial-grade triaxial accelerometer	< 30 days/ 7 days	Yes	1 sec, 5 sec	NR	NR	NR	52	2
**Fitbit Versa (**Fitbit Inc., San Francisco, CA, USA)	Commercial-grade triaxial accelerometer	2.5 GB/ 4 days	Yes	NR	NR	NR	39 x 39 x 11	38	2
**Fitbit Zip** (Fitbit Inc, San Francisco, CA, USA)	Commercial-grade triaxial accelerometer	7 days/6 Months	Yes	NR	NR	NR	28 x 35.5 x 9.65	8	10
**Freestyle Pacer Pro** ((Freestyle Brands, Carrollton, TX, USA)	Commercial-grade pedometer	NR	Yes	NR	NR	NR	NR	NR	1
**Garmin Vivofit 1** (Garmin, Schaffhausen, Switzerland)	Commercial-grade triaxial accelerometer	> 1 year	Yes	NR	NR	NR	25.5 x 10	25.5	5
**Garmin Vivofit 2** (Garmin, Schaffhausen, Switzerland)	Commercial grade triaxial accelerometer	NR/1 year	yes	NR	NR	NR	21 x 10,5 x 120	25,5	1
**Garmin Vivofit 3** (Garmin, Schaffhausen, Switzerland)	Commercial-grade triaxial accelerometer	1 year	Yes	NR	NR	NR	10 x 10	26- 28	2
**Garmin Vivosmart 4** (Garmin, Schaffhausen, Switzerland)	Commercial grade triaxial accelerometer	14 days/7 days	yes	1 min	NR	NR	15 x 10.5 x 197	16.5	1
**Garmin Vivosmart** (Garmin, Schaffhausen, Switzerland)	Commercial grade triaxial accelerometer	/7days	yes	NR	NR	NR	140 –200	19	2
**GENEActiv** (ActivInsights Ltd., Cambridgeshire, United Kingdom)	Commercial-grade triaxial accelerometer	500 MB/45 days (at 10 Hz)	No	NR	± 8	1000	43 x 40 x 13	16	7
**Geonaute** (Decathlon, Villeneuve d´Ascq France)	Commercial-grade pedometer	NR	NR	NR	NR	NR	NR	NR	1
**HealthPatch** (Vital Connect, CA, USA)	Commercial-grade device	NR	No	NR	NR	NR	NR	NR	1
**Hookie AM20** (Traxmeet Ltd, Espoo, Finland)	Research-grade triaxial accelerometer	2 GB	NR	30s	± 16	100	66 × 27 × 13	15	1

**Huawei Watch (**Huawei Technologies Co., Ltd., Longgang, China)	Commercial-grade triaxial accelerometer	14 / 7 days	Yes	NR	NR	NR	42.8 x 42.8 x 10.5	36.2	1
**Intelligent Device for Energy Expenditure and Activity (IDEEA)** (Minisun LLC, Fresno, CA, USA)	Research-grade device	200 Mb/48 hours	No	NR	NR	NR	70 x 44 x 18	59	4
**iHealth Edge** (Andon Health Co., Ltd., Tianjin, China)	Commercial-grade device	NR	Yes	NR	NR	NR	NR	NR	1
**iWown i5 Plus** (Boje Sport, Leipzig, Germany)	Commercial-grade device	NR	Yes	NR	NR	NR	46 x 18.7 x 7.9	NR	1
**Jawbone UP** (Jawbone, San Francisco, California, USA)	Commercial-grade triaxial accelerometer	9 month/10 days	N	NR	NR	NR	14.0 x 15.5 (small)	19-23	1
**Jawbone UP3** (Jawbone, San Francisco, California, USA)	Commercial-grade triaxial accelerometer	/7 days	No	30s	NR	NR	140 x 90	29	2
**Jawbone UP24** (Jawbone, San Francisco, California, USA)	Commercial-grade triaxial accelerometer	7 days	No				66–81 × 50–56	19 - 23	9
**Kellogg's* Special K* Step Counters** (Kellogg´s, Battle Creek, MI, USA)	Commercial-grade pedometer	NR	Yes	NR	NR	NR	64 x 48 x 22	21	1
**Kenz Lifecorder EX** (Suzuken, Co., Ltd., Nagoya, Japan)	Research-grade triaxial accelerometer	200 days/6 months	Yes	5 sec - 10 min	0.06-1.94	32	72.5 x 41.5 x 27.5	60	5
**Kenz e-style2** (Suzuken, Co., Ltd., Nagoya, Japan)	Research-grade triaxial accelerometer	NR	NR	NR	NR	NR	63 x 36 x 14	22	1
**kmsMove-sensor** (movisens GmbH, Karlsruhe, Germany)	Research-grade triaxial accelerometer	7 days	Yes	4s	±8	128	53 x 30 x 20	NR	1
**Kronwise 3.0** (Kronohealth, Murcia, Spain)	Research-grade triaxial accelerometer	NR	NR	NR	± 2	10	NR	NR	1
**Lifecorder** (Suzuken, Co., Ltd., Nagoya, Japan)	Research-grade uniaxial accelerometer	NR	Yes	NR	NR	NR	62 x 46 x 26	42	1
**Lifecorder PLUS** (Suzuken, Co., Ltd., Nagoya, Japan)	Research-grade uniaxial accelerometer	7 days/2 months	Yes	NR	NR	NR	75 x 42 x 29.1	48	1
**Life Microscope (**Hitachi Ltd., Tokyo, Japan)	Research-grade triaxial accelerometer	14 days/NR	No	1 min	NR	20	21 x 39 x 15.5	22	1
**Lifesource XL-18 (**A&D Medical, Toronto, ON, Canada**)**	Commercial-grade triaxial accelerometer	14 days/1 year	Yes	NR	NR	NR	76 x 34 x 11	28,35	1
**Lumoback** (Lumo Bodytech Inc., Mountain View, CA, USA)	Commercial-grade triaxial accelerometer	NR/120-168 h	No	NR	NR	NR	415 x 100 x 8	25	2
**Metria IH1 (**Vandrico Inc., Vancouver, Canada)	Commercial grade triaxial accelerometer	28 days/1 week	No	NR	NR	NR	NR	NR	1
**Microsoft Band** (Microsoft, Inc., Redmond, WA, USA)	Commercial-grade triaxial accelerometer	64 MB/48 hours	Yes	NR	NR	NR	NR	60	1
**Mio FUSE** (Mio, Vancouver, Canada)	Commercial-grade triaxial accelerometer	NR/24 hours	Yes	NR	NR	NR	30 x 259 x 16	39.7	1
**Misfit Shine (**Fossil Group, Richardson, TX, USA)	Commercial-grade triaxial accelerometer	4 months	Yes	NR	NR	NR	27.5 x 3.3 x 27.5	9.4	10
**Misfit Shine 2 (**Fossil Group, Richardson, TX, USA)	Commercial-grade triaxial accelerometer	NR/ 120 days	Yes	NR	NR	NR	30.5 x 8 x 30.5	8.5	1
**MMOXX 1.01** (Maastricht Instruments BV, Maastrich, Netherlands)	Research-grade triaxial accelerometer	NR	NR	NR	± 6	25	45 x 40 x 14	NR	1

**Motionlogger Sleepwatch** (Ambulatory Monitoring Inc., Ardsley, NY, USA)	Research-grade uniaxial accelerometer	2 MB/30 days	Yes	1 sec - 10 min	NR	10	55 x 45 x 18	65	1
**Motionlogger Microwatch Actigraph** (Ambulatory Monitoring Inc., Ardsley, New York, USA)	Research-grade triaxial accelerometer	NR	Yes	30 sec	NR	NR	NR	NR	1
**MotionSense HRV** (MD2K Center, Memphis, USA)	Research-grade triaxial accelerometer	NR	No	NR	2	16	NR	NR	1
**MotionWatch 8** (CamNTech, Cambridge, UK)	Research-grade triaxial accelerometer	4 MB/ 3 months	No	1-60 sec	0.01 - 8	50	36 x 28.2 x 9.4	9.1	2
**MovBand Model 2** (Movband, LLC, Brecksville, OH, USA)	Commercial-grade triaxial accelerometer	40 days	Yes	NR	2-8	NR	NR	NR	1
**MSR2005** (Electronics GmbH, Henggart, Switzerland)	Research-grade uniaxial accelerometer	NR	No	1 min	2	256	NR	NR	1
**MSR2010** (Electronics GmbH, Henggart, Switzerland)	Research-grade triaxial accelerometer	NR	No	1 min	2	256	NR	NR	1
**MyKronoz ZeFit4** (Kronoz LLC, Genf, Switzerland)	Commercial-grade triaxial accelerometer	5 days	Yes	NR	NR	NR	45.5 x 18.6 x 9.3	17	1
**MyWellness Key** (Technogym, Gambettola, Italy)	Commercial-grade uniaxial accelerometer	49-59 days	Yes	NR	0.06 - 12	16	85 x 20 x 7	18.7	2
**New Lifestyle NL-1000** (New Lifestyles, Inc., Lee´s Summit, MO, USA)	Commercial-grade uniaxial accelerometer	7 days/18 months	Yes	4 sec /min	NR	NR	63.5 x 38.1 x 22.2	31.2	1
**New Lifestyles NL-2000** (New Lifestyles, Inc., Lee´s Summit, MO, USA)	Commercial-grade triaxial accelerometer	7-14 days/ g months	Yes	NR	NR	NR	63.5 x 38.1 x 12.7	25.5	3
**Nike + FuelBand SE** (Nike Inc., Beaverton, OR, USA)	Commercial-grade triaxial accelerometer	4 days	Yes	NR	NR	NR	147–197 × 19	27 - 32	5
**Nokia GO** (Nokia Corporation, Espoo, Finland)	Commercial-grade triaxial accelerometer	8 months	Yes	NR	NR	NR	34.5 x 9.4	9	2
**Omron CaloriScan HJA-401F** (Omron Healthcare, Inc., Vernon Hills, IL, USA)	Commercial-grade device	7 days	Yes	NR	NR	NR	NR	NR	1
**Omron HJ-105** (Omron Healthcare, Inc., Vernon Hills, IL, USA)	Commercial-grade device	7 days/ 3 months	Yes	NR	NR	NR	63.5 x 38.1 x 25.4	24	2
**Omron HJ-203** (Omron Healthcare, Inc., Vernon Hills, IL, USA)	Commercial-grade biaxial accelerometer	7 days/ 1.5 yrs	Yes	NR	NR	NR	35.5 x 68.5 x 11.0	19	2
**Omron CaloriScan HJ-306** (Omron Healthcare, Inc., Vernon Hills, IL, USA)	Commercial-grade triaxial accelerometer	7 days/ 6 months	Yes	NR	NR	NR	78 x 33 x 10	25	1
**Omron HJ-322U-E** (Omron Healthcare, Inc., Vernon Hills, IL, USA)	Commercial-grade device	7 days/ 6 months	Yes	NR	NR	NR	78 x 34 x 12	27	2
**Omron Walking Style Pro HJ-720IT** (Omron Healthcare, Inc., Bannockburn, IL, USA)	Commercial-grade biaxial accelerometer	41 days/ approx. 6 months	Yes	NR	NR	NR	47 x 73 x 16	37	6
**Omron Active Style Pro HJA-350IT** (Omron Healthcare, Inc., Bannockburn, IL, USA)	Commercial-grade triaxial accelerometer	4 GB/ NR	Yes	10-60 sec	± 6	32	74x 46 x 34	60	2
**Omron Active Style Pro HJA-750C** (Omron Healthcare, Inc., Vernon Hills, IL, USA)	Commercial-grade triaxial accelerometer	NR/2 months	Yes	NR	± 6	32	40 x 52 x 12	23	1
**Oregon Scientific PE829** (Oregon Scientific, Tualatin, OR, USA)	Commercial-grade pedometer	7 day	Yes	NR	NR	NR	61.4 x 53.5 x 26	60	1

**Oregon Scientific PE316CA** (Oregon Scientific, Tualatin, OR, USA)	Commercial-grade pedometer	NR	Yes	NR	NR	NR	32 x 41 x 63	28	1
**PiezoRx** (StepsCount, Ontario, Canada)	Commercial-grade pedometer	33 days	Yes	NR	NR	NR	NR	NR	2
**Polar Active Watch** (Polar Electro Oy, Kimpel, Finland)	Commercial-grade uniaxial accelerometer	21 days/4 days	Yes	30 sec	NR	0.3-4	NR	45	2
**Polar activity recorder FA20** (Polar Electro Oy, Kimpel, Finland)	Commercial-grade uniaxial accelerometer	NR/1.5 yrs	Yes	NR	NR	NR	NR	NR	1
**Polar A300** (Polar Electro Oy, Kimpel, Finland)	Commercial-grade triaxial accelerometer	60 hours/200 hours	Yes	NR	NR	NR	115 x 95 x 74.9	48	1
**Polar A360** (Polar Electro Oy, Kimpel, Finland)	Commercial-grade triaxial accelerometer	60 hours/NR	Yes	NR	NR	NR	NR	33.7	1
**Polar Loop** (Polar Electro Oy, Kimpel, Finland)	Commercial-grade triaxial accelerometer	4 Mb/6 days	Yes	NR	NR	NR	145–240 × 20	38	3
**Polar M430** (Polar Electro Oy, Kimpel, Finland)	Commercial-grade triaxial accelerometer	20 days	Yes	NR	NR	50	12 x 128 x 128	51	1
**Polar M600** (Polar Electro Oy, Kimpel, Finland)	Commercial-grade triaxial accelerometer	NR	Yes	NR	NR	NR	NR	63	1
**Polar RS800CX** (Polar Electro Oy, Kimpel, Finland)	Commercial-grade uniaxial accelerometer	NR/1 yr	Yes	NR	NR	NR	NR	NR	1
**Polar Sports Tester 1000 HR** (Polar Electro Oy, Kimpel, Finland)	Commercial-grade device	NR	Yes	NR	NR	NR	NR	NR	1
**Polar V800** (Polar Electro Oy, Kimpel, Finland)	Commercial-grade triaxial accelerometer	60 hours/30 days	Yes	NR	NR	NR	37 x 56 x 12.7	79	1
**RT3** (Stayhealthy, Inc., Monrovia, CA, USA)	Research-grade triaxial accelerometer	21 days/ 8.5 days	No	1 - 60 sec	± 250	0.7 - 5000	71 x 56 x 28	65.2	3
**Runscribe inertial sensors** (Scribe Labs, CA, USA)	Research-grade triaxial accelerometer	NR	No	NR	NR	10	NR	NR	1
**Samsung Gear Fit2** (Samsung Group, Seoul, South Korea)	Commercial-grade triaxial accelerometer	32 MB/ 15 days	Yes	NR	NR	NR	46.6 x 1.6 x 11.1	21	1
**SENS Motion System** (SENS Innovation ApS, Copenhagen, Denmark**)**	Commercial-grade triaxial accelerometer	14 days/20 weeks	No	NR	± 4	12.5	50 x 21 x 5	8	1
**SenseWear Pro** (BodyMedia, Inc., Pittsburgh, PA)	Commercial-grade biaxial accelerometer	5 days /4 days	No	NR	NR	32	85.3 x 53.4 x 19.5	85	4
**SenseWear Pro 2** (BodyMedia, Inc., Pittsburgh, PA)	Commercial-grade biaxial accelerometer	NR/2 weeks	No	60 sec	NR	32	NR	NR	2
**SenseWear Pro 3** (BodyMedia, Pittsburgh, PA, USA)	Commercial-grade biaxial accelerometer	NR/2 weeks	No	NR	± 2	NR	85 x 53 x 19	79	7
**SenseWear Mini** (BodyMedia, Pittsburgh, PA, USA)	Commercial-grade triaxial accelerometer	NR	No	NR	± 1	1	86 x 60 x 27	45.36	4
**Silva pedometer model 56012** (Silva Sweden AB, Stockholm, Sweden)	Research-grade pedometer	12 months	Yes	NR	NR	NR	NR	19	1
**SitFIT** (PAL Technologies Limited, Glasgow, UK)	Research-grade triaxial accelerometer	NR	No	NR	NR	NR	NR	NR	1
**Sleepwatch-O** (Ambulatory Monitoring, Inc, Ardsley, NY, USA)	Research-grade uniaxial accelerometer	2 Mb/ 60 days	Yes	NR	NR	NR	NR	NR	2
**SmartLAB walk +** (HMM Diagnostics GmbH, Dossenheim, Germany)	Commercial-grade triaxial accelerometer	7 days memory	Yes	NR	NR	NR	70 x 37 x 10.5	30	1

**Sportline 330** (E&B Giftware LLC, Hazleton, PA, USA)	Commercial-grade pedometer	NR/NR	Yes	NR	NR	NR	19.7 x 5.9 x 27.6	90.7	2
**Sportline 343** (E&B Giftware LLC, Hazleton, PA, USA)	Commercial-grade pedometer	NR/NR	Yes	NR	NR	NR	NR	68	1
**Sportline 345** (E&B Giftware LLC, Hazleton, PA, USA)	Commercial-grade pedometer	NR/NR	Yes	NR	NR	NR	78 x 43 x 17	45	1
**Sport Tester PE 400** (Polar Electro Oy, Kimpel, Finland)	Commercial-grade device	NR/NR	Yes	NR	NR	NR	NR	NR	1
**Stepwatch Activity Monitor** (Cyma Corp., Mountlake Terrace, WA, USA)	Research-grade biaxial accelerometer	NR/NR	No	NR	NR	NR	50 x 15 x 65	65	2
**Stepwatch 3 Activity Monitor** (Cyma Corp., Mountlake Terrace, WA, USA)	Research-grade triaxial accelerometer	32 KB/7 years	No	NR	NR	NR	75 x 50 x 20	38	1
**Striiv Smart Pedometer** (Striiv, Inc. Redwood City, CA, USA)	Commercial-grade pedometer	NR/NR	Yes	NR	NR	NR	82.5 x 50 x 32.5	39.7	1
**Tanita AM-160** (Tanita Europe BV, Amsterdam, Netherlands)	Commercial-grade triaxial accelerometer	7 days/NR	Yes	NR	NR	NR	75 x 14 x 35	26	1
**Tanita Calorism Smart** (Tanita Europe BV, Amsterdam, Netherlands)	Commercial-grade triaxial accelerometer	NR/NR	Yes	NR	NR	NR	55 x 11.8 x 29	23	1
**$\textbf{Tracmor}_\textbf{D}$** (Philips DirectLife, Amsterdam, Netherlands)	Research-grade triaxial accelerometer	22 weeks/3 weeks	No	1 min	NR	20	32 x 32 x 5	12.5	3
**Tritrac-R3D** (Professional Products, Madison, WI, USA)	Research-grade triaxial accelerometer	14 days/ NR	NR	1 - 15 min	0.05 - 6.3	0.1 - 3.0	120 x 65 x 22	170	1
**Verisense** (Shimmer Research Ltd., Dublin, Ireland)	Research-grade triaxial accelerometer	NR/6 months	No	NR	NR	12.5-1600	43 x 35 x 12	29.6	1
**VeryFit 2.0** (OEM, Guangdong, China)	Commercial-grade triaxial accelerometer	NR/5 days	Yes	NR	NR	NR	NR	18	1
**VitaBit** (Ipmit, Ljunljana, Slovenia)	Research-grade triaxial accelerometer	NR/>30 days	No	NR	16	33	39 x 14 x 8.5	4.8	1
**Vitaport 2 (**Temec Instruments BV, PC Heerlen, Netherlands)	Research-grade device	NR	NR	NR	NR	32	150 x 90 x 45	700	1
**Walk4Life LS 2525** (Walk4Life, Inc., Plainfield, IL, USA)	Commercial-grade pedometer	NR/NR	Yes	NR	NR	NR	NR	NR	1
**Withings Activite** (Withings SA, Issy les Moulineaux, France)	Commercial-grade triaxial accelerometer	NR/8 months	No	NR	NR	NR	50 x 50 x 50	37	1
**Withings GO** (Withings SA, Issy les Moulineaux, France)	Commercial-grade triaxial accelerometer	NR/8 months	Yes	NR	NR	NR	3.6 x 1.1 x 19.5	NR	1
**Withings Pulse** (Withings SA, Issy les Moulineaux, France)	Commercial-grade triaxial accelerometer	NR/14 days	Yes	NR	NR	NR	22 × 8 × 43	8	3
**Withings Pulse O2** (Withings SA, Issy les Moulineaux, France)	Commercial-grade triaxial accelerometer	NR/14 days	Yes	NR	NR	NR	22 x 8 x 43	8	4
**WHOOP Strap 2.0** (WHOOP Inc. Boston, MA, USA)	Commercial-grade triaxial accelerometer	NR/36 h	No	NR	NR	NR	25.4 x 245	18.1	1
**Xiaomi Mi Band** (Xiaomi, Beijing, China)	Commercial-grade triaxial accelerometer	16 MB/60 days	Yes	NR	NR	NR	37 x 13.6 x 9.9	13	2
**Xiaomi Mi Band 2** (Xiaomi, Beijing, China)	Commercial-grade triaxial accelerometer	20 days	Yes	NR	NR	NR	15.7 x 40.3 x 10.5	19	2

**Yamax Digiwalker CW-700** (Yamax Corp., Tokyo, Japan)	Commercial-grade pedometer	2 weeks/3 years	Yes	NR	NR	NR	61 x 19 x 46	38	1
**Yamax Digiwalker SW-701** (Yamax Corp., Tokyo, Japan)	Commercial-grade pedometer	None/3 yrs	Yes	NR	0.35-0.50	NR	50 x 38 x 14	21	2
**Yamax Digiwalker SW-200** (Yamax Corp., Tokyo, Japan)	Commercial grade pedometer	None/ 3 yrs	Yes	1, 2, 5 sec	0.35-0.50	NR	50 x 38 x 14	21	7
**Yamax Skeletone EM-180** (Yamax Corp., Tokyo, Japan)	Commercial-grade pedometer	NR/ 3 yrs	Yes	NR	NR	NR	48 x 36 x 12.3	16	1
**Yamax Digiwalker DW-500** (Yamax Corp., Tokyo, Japan)	Commercial-grade pedometer	NR/ 3 yrs	Yes	NR	NR	NR	50 x 38 x 14	21	1

[1]NR: not reported

Table S7. Risk of bias for the included studies.

Article Nr.	Author	Patient Selection/Study design	Index measure	Criterion measure	Flow & Timing
1	Agogo et al., 2018	HIGH	UNCLEAR	LOW	LOW
2	Albright & Jerome, 2011	HIGH	HIGH	HIGH	HIGH
3	Alharbi et al., 2016	LOW	HIGH	HIGH	LOW
4	Ameen et al., 2019	LOW	HIGH	LOW	LOW
5	An et al., 2017	HIGH	HIGH	HIGH	HIGH
6	Ancoli- Israel et al., 1997	HIGH	HIGH	LOW	HIGH
7	Annegarn et al., 2011	HIGH	HIGH	LOW	HIGH
8	Assah et al., 2011	HIGH	LOW	LOW	LOW
9	Au-Yeung et al., 2020	HIGH	HIGH	HIGH	HIGH
10	Baandrup & Jenum, 2015	HIGH	HIGH	LOW	HIGH
11	Bai et al., 2021	HIGH	HIGH	HIGH	LOW
12	Barkley et al., 2019	HIGH	HIGH	HIGH	HIGH
13	Barone-Gibbs et al., 2020	LOW	HIGH	HIGH	HIGH
14	Barreira et al., 2015	HIGH	HIGH	HIGH	LOW
15	Bartholdy et al., 2018	HIGH	UNCLEAR	HIGH	LOW
16	Beattie et al., 2017	HIGH	HIGH	LOW	LOW
17	Berendsen et al., 2014	HIGH	HIGH	HIGH	LOW
18	Berninger et al., 2018	HIGH	HIGH	HIGH	LOW
19	Berryhill et al., 2020	HIGH	HIGH	LOW	LOW
20	Blackwell et al., 2008	LOW	LOW	LOW	HIGH
21	Blackwell et al., 2011	LOW	LOW	LOW	HIGH
22	Block et al., 2019	LOW	HIGH	HIGH	LOW
23	Blondeel et al., 2020	LOW	HIGH	HIGH	HIGH
24	Boeselt et al., 2016	LOW	HIGH	HIGH	HIGH
25	Bonomi et al., 2009	HIGH	LOW	LOW	LOW
26	Bonomi et al., 2010	HIGH	HIGH	LOW	LOW
27	Bourke et al., 2016	HIGH	UNCLEAR	LOW	UNCLEAR
28[a]	Bourke et al., 2019	HIGH	HIGH	LOW	UNCLEAR
28[b]	Bourke et al., 2019	HIGH	HIGH	LOW	UNCLEAR
29	Brage et al., 2015	HIGH	LOW	LOW	LOW
30	Brazeau et al., 2016	LOW	HIGH	LOW	LOW
31	Breteler et al., 2019	LOW	HIGH	HIGH	HIGH
32	Brewer et al., 2017	HIGH	HIGH	HIGH	HIGH
33[a]	Brooke et al., 2017	HIGH	HIGH	HIGH	LOW
33[c]	Brooke et al., 2017	HIGH	HIGH	HIGH	LOW
34	Bruignaux et al., 2010	HIGH	HIGH	LOW	HIGH
35	Busse et al., 2009	LOW	HIGH	HIGH	LOW
36	Bussmann et al., 1998	HIGH	HIGH	LOW	LOW
37	Cabanas-Sánchez et al., 2018	HIGH	LOW	HIGH	HIGH
38	Calabro et al., 2015	LOW	HIGH	LOW	LOW
39	Campos et al., 2018	HIGH	HIGH	HIGH	HIGH
40	Carpenter et al., 2021	HIGH	HIGH	HIGH	HIGH
41	Carter et al., 2008	HIGH	LOW	LOW	LOW
42	Castner et al., 2019	LOW	HIGH	HIGH	HIGH
43[a]	Cellini et al., 2016	LOW	HIGH	HIGH	LOW
43[c]	Cellini et al., 2016	LOW	HIGH	HIGH	LOW
44	Chakravarthy & Resnick, 2017	HIGH	HIGH	HIGH	LOW
45	Chocquette et al., 2009	HIGH	HIGH	LOW	LOW
46	Chowdhury et al., 2017	HIGH	HIGH	HIGH	HIGH
47	Chu et al., 2017	LOW	HIGH	HIGH	HIGH
48	Clemes et al., 2010	HIGH	HIGH	HIGH	HIGH
49	Colbert et al., 2011	LOW	LOW	LOW	LOW
50	Collins et al., 2019	HIGH	LOW	HIGH	HIGH
51	Connolly et al., 2020	LOW	HIGH	HIGH	HIGH
52	Correa et al., 2016	LOW	UNCLEAR	LOW	LOW
53	Cuberek et al., 2010	HIGH	HIGH	HIGH	HIGH
54	Culhane et al., 2004	HIGH	HIGH	HIGH	LOW
55	Davidson et al., 1997	HIGH	UNCLEAR	LOW	LOW
56	De Cocker et al., 2012	HIGH	HIGH	HIGH	HIGH
57	Degroote et al., 2018	LOW	HIGH	HIGH	HIGH

58[a]	Degroote et al., 2020	HIGH	HIGH	HIGH	HIGH
58[c]	Degroote et al., 2020	HIGH	HIGH	HIGH	HIGH
59	DeShaw et al., 2018	LOW	HIGH	HIGH	LOW
60	Dickinson et al., 2016	HIGH	HIGH	HIGH	HIGH
61	Dieu et al., 2016	HIGH	HIGH	HIGH	HIGH
62	Dominick et al., 2016	HIGH	LOW	HIGH	LOW
63	Donahoe et al., 2018	LOW	HIGH	HIGH	LOW
64	Dondzila et al., 2012	HIGH	HIGH	HIGH	HIGH
65	Dondzila et al., 2018	HIGH	HIGH	HIGH	HIGH
66	Durkalec-Michalski et al., 2013	HIGH	UNCLEAR	HIGH	HIGH
67	Edwardson et al., 2018	LOW	HIGH	HIGH	HIGH
68	Ekelund et al., 2000	HIGH	HIGH	LOW	LOW
69	Farina & Lowry, 2017	LOW	HIGH	HIGH	HIGH
70	Feito et al., 2015	LOW	HIGH	HIGH	LOW
71	Feito et al., 2012	LOW	HIGH	HIGH	LOW
72[a]	Ferguson et al., 2015	LOW	UNCLEAR	HIGH	HIGH
72[c]	Ferguson et al., 2015	LOW	LOW	HIGH	HIGH
73[a]	Fokkerood et al., 2014	HIGH	HIGH	LOW	LOW
73[b]	Fokkerood et al., 2014	HIGH	HIGH	LOW	LOW
74	Full et al., 2018	HIGH	LOW	LOW	LOW
75	Fuller et al., 2017	LOW	LOW	LOW	LOW
76	Fullerton et al., 2017	HIGH	LOW	LOW	LOW
77	Gardner & Poehlman, 1998	LOW	HIGH	LOW	LOW
78	Garnotel et al., 2020	LOW	LOW	LOW	LOW
79[a]	Gill et al., 2018	LOW	LOW	HIGH	LOW
79[b]	Gill et al., 2018	LOW	LOW	HIGH	LOW
80	Godfrey et al., 2007	HIGH	HIGH	HIGH	LOW
81	Gomersall et al., 2016	LOW	HIGH	HIGH	HIGH
82[a]	Gruwez et al., 2017	HIGH	HIGH	HIGH	HIGH
82[c]	Gruwez et al., 2017	HIGH	HIGH	LOW	HIGH
83	Haghayegh et al., 2019	HIGH	HIGH	HIGH	LOW
84	Hamill et al., 2019	LOW	HIGH	HIGH	HIGH
85	Hargens et al., 2017	LOW	HIGH	HIGH	HIGH
86	Härtel et al., 2011	HIGH	HIGH	LOW	LOW
87	Henriksen et al., 2019	HIGH	LOW	HIGH	HIGH
88	Hernandez-Vicente et al., 2016	HIGH	HIGH	HIGH	HIGH
89	Hermann et al., 2011	HIGH	HIGH	HIGH	HIGH
90	Hickey et al., 2017	HIGH	HIGH	LOW	LOW
91	Hickey et al., 2016	HIGH	HIGH	HIGH	HIGH
92	Höchsmann et al., 2021	LOW	HIGH	HIGH	HIGH
93	Hollewand et al., 2016	HIGH	HIGH	HIGH	LOW
94	Hui et al., 2018	HIGH	HIGH	HIGH	HIGH
95	Jean-Louis et al., 2001	HIGH	LOW	LOW	LOW
96	Jenkins et al., 2021	LOW	LOW	HIGH	LOW
97	Johannsen et al., 2010	LOW	LOW	LOW	LOW
98	Johannson et al., 2006	HIGH	HIGH	LOW	LOW
99	Judice et al., 2015	HIGH	HIGH	HIGH	LOW
100	Judice et al., 2019	HIGH	HIGH	HIGH	LOW
101	Kanda et al., 2012	HIGH	HIGH	HIGH	LOW
102	Kang et al., 2017	HIGH	HIGH	LOW	LOW
103	Kawada, 2008	HIGH	LOW	HIGH	LOW
104	Keating et al., 2012	HIGH	HIGH	HIGH	LOW
105	Kerr et al., 2013	LOW	HIGH	LOW	LOW
106	Kerr et al., 2018	LOW	LOW	HIGH	HIGH
107	Kim et al., 2015	HIGH	HIGH	LOW	LOW
108	Kinnunen et al., 2012	HIGH	LOW	LOW	LOW
109	Kinnunen et al., 2019	HIGH	LOW	LOW	LOW
110	Koehler et al., 2011	HIGH	HIGH	LOW	LOW
111	Koenders et al., 2018	HIGH	HIGH	LOW	LOW
112	Kogure et al., 2011	HIGH	LOW	LOW	HIGH
113	Kooiman et al., 2015	HIGH	HIGH	HIGH	HIGH
114	Kozey-Keadle et al., 2011	HIGH	HIGH	HIGH	LOW
115	Kubala et al., 2020	LOW	HIGH	HIGH	LOW
116	Kumahara et al., 2015	HIGH	HIGH	HIGH	HIGH

117	Kurita et al., 2017	LOW	LOW	HIGH	HIGH
118	Kwon et al., 2021	LOW	HIGH	HIGH	HIGH
119	Latshang et al., 2016	LOW	HIGH	LOW	LOW
120	Le Masurier et al., 2004	HIGH	HIGH	HIGH	UNCLEAR
121[a]	Lebleau et al., 2020	HIGH	HIGH	HIGH	HIGH
121[c]	Lebleau et al., 2020	HIGH	HIGH	HIGH	HIGH
122	Lee & Laurson, 2015	LOW	HIGH	HIGH	HIGH
123	Lee & Suen, 2017	LOW	LOW	HIGH	HIGH
124	Lee et al., 2018	LOW	LOW	HIGH	LOW
125	Lee et al., 2018	LOW	LOW	HIGH	HIGH
126	Lee et al., 2015	HIGH	UNCLEAR	HIGH	HIGH
127	Leenders et al., 2006	HIGH	LOW	LOW	LOW
128	Li et al., 2020	HIGH	LOW	LOW	HIGH
129	Liu et al., 2015	HIGH	HIGH	HIGH	HIGH
130	Löf et al., 2013	HIGH	UNCLEAR	LOW	UNCLEAR
131	Lyden et al., 2012	HIGH	HIGH	HIGH	LOW
132	Lyden et al., 2017	HIGH	LOW	HIGH	HIGH
133	Mackey et al., 2011	HIGH	LOW	LOW	LOW
134	Madrid-Navarro et al., 2019	HIGH	LOW	LOW	LOW
135	Manuta et al., 2016	HIGH	HIGH	LOW	LOW
136	Marcotte et al., 2020	HIGH	HIGH	LOW	LOW
137[a]	Matthews et al., 2018	LOW	LOW	LOW	HIGH
137[b]	Matthews et al., 2018	LOW	LOW	HIGH	LOW
138[a]	McDevitt et al., 2021	HIGH	UNCLEAR	HIGH	LOW
138[c]	McDevitt et al., 2021	HIGH	LOW	HIGH	LOW
139	McGinley et al., 2015	LOW	HIGH	HIGH	LOW
140	McVeigh et al., 2021	LOW	LOW	HIGH	HIGH
141	Middelweerd et al., 2021	LOW	HIGH	HIGH	HIGH
142	Mikkelsen et al., 2020	LOW	LOW	HIGH	HIGH
143	Miyamoto et al., 2018	HIGH	HIGH	HIGH	HIGH
144	Moore et al., 2012	HIGH	HIGH	LOW	LOW
145	Mouritzen et al., 2020	HIGH	LOW	LOW	LOW
146	Murakami et al., 2019	HIGH	HIGH	LOW	LOW
147	Myers et al., 2014	HIGH	UNCLEAR	HIGH	UNCLEAR
148	Narayanan et al., 2020	HIGH	HIGH	LOW	LOW
149	Nguyen et al., 2013	HIGH	HIGH	LOW	LOW
150	O´Brien et al., 2018	HIGH	HIGH	HIGH	HIGH
151	O´Brien et al., 2020	LOW	LOW	HIGH	HIGH
152	O´Neill et al., 2017	LOW	HIGH	HIGH	HIGH
153	Paul et al., 2015	LOW	HIGH	HIGH	HIGH
154	Pavey et al., 2016	HIGH	LOW	HIGH	HIGH
155	Pomeroy et al., 2011	LOW	HIGH	LOW	LOW
156	Quante et al., 2018	LOW	LOW	LOW	LOW
157	Rabinovich et al., 2013	LOW	HIGH	LOW	LOW
158	Rafamantanantsoa et al., 2002	LOW	UNCLEAR	LOW	LOW
159	Redenius et al., 2019	LOW	HIGH	HIGH	LOW
160	Regalia et al., 2020	HIGH	LOW	LOW	LOW
161	Reid et al., 2017	HIGH	HIGH	HIGH	HIGH
162[a]	Rosenberger et al., 2016	HIGH	UNCLEAR	HIGH	HIGH
162[b]	Rosenberger et al., 2016	HIGH	UNCLEAR	HIGH	HIGH
162[c]	Rosenberger et al., 2016	HIGH	LOW	HIGH	HIGH
163	Rothney et al., 2010	HIGH	LOW	LOW	LOW
164	Rousset et al., 2014	HIGH	HIGH	LOW	LOW
165	Rozanski et al., 2018	HIGH	HIGH	HIGH	HIGH
166	Rutgers et al., 1997	HIGH	HIGH	HIGH	LOW
167	Sánchez-Ortuño et al., 2010	HIGH	HIGH	LOW	LOW
168	Sargent et al., 2016	HIGH	LOW	LOW	LOW
169	Sasaki et al., 2018	LOW	LOW	HIGH	HIGH
170	Schmal et al., 2018	LOW	UNCLEAR	LOW	HIGH
171	Schneider et al., 2004	HIGH	HIGH	HIGH	HIGH
172[a]	Scott et al., 2019	HIGH	UNCLEAR	HIGH	LOW
172[c]	Scott et al., 2019	HIGH	HIGH	HIGH	LOW
173	Semanik et al., 2020	LOW	LOW	HIGH	HIGH
174	Siddall et al., 2019	HIGH	LOW	LOW	LOW
175	Silcott et al., 2011	HIGH	HIGH	HIGH	HIGH

176	Silva et al., 2015	HIGH	UNCLEAR	LOW	LOW
177	Silva et al., 2019	HIGH	HIGH	HIGH	HIGH
178	Simunek et al., 2016	LOW	HIGH	HIGH	HIGH
179	Sjöberg et al., 2021	LOW	HIGH	HIGH	HIGH
180	Skipworth et al., 2011	HIGH	UNCLEAR	LOW	LOW
181	Skotte et al., 2014	HIGH	UNCLEAR	HIGH	HIGH
182	Slinde et al., 2013	HIGH	LOW	LOW	LOW
183	Smits et al., 2018	LOW	LOW	HIGH	LOW
184	St-Laurent et al., 2018	HIGH	HIGH	HIGH	HIGH
185	Stein et al., 2003	HIGH	LOW	HIGH	HIGH
186	St-Onge et al., 2018	LOW	HIGH	LOW	LOW
187	Strath et al., 2005	HIGH	HIGH	LOW	LOW
188	Sugino et al., 2011	HIGH	HIGH	HIGH	HIGH
189	Sushames et al., 2016	HIGH	HIGH	HIGH	HIGH
190	Svensson et al., 2019	LOW	HIGH	HIGH	LOW
191	Te Lindert et al., 2013	HIGH	LOW	HIGH	LOW
192[a]	Tedesco et al., 2019	HIGH	HIGH	HIGH	LOW
192[c]	Tedesco et al., 2019	HIGH	HIGH	HIGH	LOW
193	Thorup et al., 2017	HIGH	HIGH	HIGH	HIGH
194	Toth et al., 2018	HIGH	HIGH	LOW	LOW
195	Tudor-Locke et al., 2006	HIGH	HIGH	HIGH	UNCLEAR
196	Tully et al., 2014	LOW	HIGH	HIGH	HIGH
197	Uiterwaal et al., 1998	HIGH	HIGH	LOW	LOW
198	Vähä-Ypyä et al., 2017	HIGH	LOW	HIGH	HIGH
199	Valenti et al., 2014	HIGH	LOW	LOW	LOW
200	Van Alphen et al., 2020	HIGH	HIGH	LOW	HIGH
201	Van Blarigan et al., 2017	LOW	HIGH	HIGH	LOW
202	Van de Wouw et al., 2013	HIGH	HIGH	LOW	LOW
203	Van den Berg-Emons et al., 2000	HIGH	HIGH	LOW	LOW
204	Van der Weegen et al., 2015	LOW	LOW	HIGH	LOW
205	Van Hees et al., 2011	LOW	LOW	LOW	LOW
206	Van Hees et al., 2018	LOW	LOW	HIGH	LOW
207	Van Nassau et al., 2015	HIGH	HIGH	HIGH	HIGH
208	Vanhelst et al., 2012	HIGH	HIGH	HIGH	HIGH
209	Varelo Mato et al., 2017	LOW	HIGH	HIGH	HIGH
210	Vetrovsky et al., 2019	LOW	HIGH	HIGH	LOW
211	Villars et al., 2012	LOW	LOW	LOW	LOW
212	Washburn et al., 1990	HIGH	HIGH	HIGH	LOW
213	Webber & St. John, 2016	HIGH	HIGH	HIGH	HIGH
214	Welk et al., 2007	HIGH	UNCLEAR	HIGH	LOW
215	Welk et al., 2017	HIGH	HIGH	HIGH	HIGH
216	White et al., 2019	LOW	HIGH	LOW	LOW
217	Whybrow et al., 2013	HIGH	HIGH	LOW	LOW
218	Williams et al., 2020	HIGH	HIGH	LOW	HIGH
219	Winkler et al., 2016	HIGH	LOW	HIGH	LOW
220	Wu et al., 2021	HIGH	UNCLEAR	HIGH	HIGH
221	Yamada et al., 2018	LOW	UNCLEAR	LOW	LOW
222	Yoshida et al., 2019	HIGH	HIGH	LOW	LOW

[a] Intensity outcome; [b] Posture/Activity type outcome; [c] Biological state outcome
